# ChatGPT: Literate or intelligent about UN sustainable development goals?

**DOI:** 10.1371/journal.pone.0297521

**Published:** 2024-04-24

**Authors:** Raghu Raman, Hiran H. Lathabai, Santanu Mandal, Payel Das, Tavleen Kaur, Prema Nedungadi

**Affiliations:** 1 Amrita School of Business, Amrita Vishwa Vidyapeetham, Amritapuri, Kerala, India; 2 Amrita Vishwa Vidyapeetham, Amritapuri, Kerala, India; 3 Amrita School of Business, Amaravati, Andhra Pradesh, India; 4 Fortune Institute of International Business, New Delhi, India; Babes-Bolyai University, Cluj-Napoca, ROMANIA

## Abstract

Generative AI tools, such as ChatGPT, are progressively transforming numerous sectors, demonstrating a capacity to impact human life dramatically. This research seeks to evaluate the UN Sustainable Development Goals (SDGs) literacy of ChatGPT, which is crucial for diverse stakeholders involved in SDG-related policies. Experimental outcomes from two widely used Sustainability Assessment tests–the UN SDG Fitness Test and Sustainability Literacy Test (SULITEST) ‐ suggest that ChatGPT exhibits high SDG literacy, yet its comprehensive SDG intelligence needs further exploration. The Fitness Test gauges eight vital competencies across introductory, intermediate, and advanced levels. Accurate mapping of these to the test questions is essential for partial evaluation of SDG intelligence. To assess SDG intelligence, the questions from both tests were mapped to 17 SDGs and eight cross-cutting SDG core competencies, but both test questionnaires were found to be insufficient. SULITEST could satisfactorily map only 5 out of 8 competencies, whereas the Fitness Test managed to map 6 out of 8. Regarding the coverage of the Fitness Test and SULITEST, their mapping to the 17 SDGs, both tests fell short. Most SDGs were underrepresented in both instruments, with certain SDGs not represented at all. Consequently, both tools proved ineffective in assessing SDG intelligence through SDG coverage. The study recommends future versions of ChatGPT to enhance competencies such as collaboration, critical thinking, systems thinking, and others to achieve the SDGs. It concludes that while AI models like ChatGPT hold considerable potential in sustainable development, their usage must be approached carefully, considering current limitations and ethical implications.

## 1. Introduction

In recent years, the proliferation of Artificial Intelligence (AI) in daily activities has been notable, with its applications expanding rapidly [1]. AI has the potential to revolutionize and enhance numerous tasks across sectors, leveraging machine learning algorithms and autonomous decision-making to foster innovation [2]. One significant branch of AI is Natural Language Processing (NLP) [3]. Large Language Models (LLMs), a subtype of NLP, can generate human-like responses to prompts and execute a broad range of language-processing tasks on enormous volumes of text data [4]. The influence of AI and LLMs is extensive in various domains. For instance, Di Vaio et al. [5] researched AI’s effect on the production and consumption of resources, striving for sustainable resource management aligned with the United Nations’ 2030 Sustainable Development Goals (SDGs). Given AI’s wide-ranging applications across sectors, including healthcare [6–8], education [9–11], manufacturing [12], marketing [13,14], finance [15], and supply chain and logistics [16], it is crucial to explore the optimal uses of AI-driven LLMs for text-generation capabilities.

ChatGPT, or Chat Generative Pre-Trained Transformer (GPT), an innovative addition to language models, was developed in November 2022 [17]. Some researchers regard it as a game-changer [18]. Since its inception, ChatGPT, a freemium model [2], has attracted significant media attention due to its remarkable performance and ability to generate human-like responses to user queries. This has led to its coverage in esteemed publications such as The New York Times [19], The Washington Post [20], Nature [21], and Wired [22], among others.

The introduction of the GPT in 2018 represented a substantial leap forward in LLMs. GPT, trained on a 40GB text dataset using a modified transformer architecture, had 1.5 billion parameters. GPT-3, released by OpenAI in 2020, was particularly impressive, with a model size of 175 billion parameters trained on a vast 570GB text dataset [23]. Apart from ChatGPT, OpenAI has also developed GPT-3 and GPT-4 as large language models, while Google has popular models such as LAMDA, BERT, and T5 [2,4]. ChatGPT, or GPT-3.5, is the latest GPT-3 iteration designed for conversational user interactions [24]. GPT-4, in contrast, has the added capability of analyzing and commenting on images and graphics [25]. This implies that GPT-4 can describe the content of an image, identify trends in a chart, or even generate image captions, which solidifies its standing as a valuable tool for education and content generation [26].

The evolution of AI, specifically LLMs like ChatGPT, is a significant step forward in technology’s ability to engage in and understand human-like conversation. These advancements allow more nuanced and effective interactions across many fields and applications. For instance, in healthcare, AI could potentially assist in diagnosing conditions, or in education, it could aid in developing personalized learning plans. However, despite the many beneficial applications of AI and LLMs, it is crucial to acknowledge their associated challenges. For example, biases embedded in the training data can lead to skewed or discriminatory outputs. Furthermore, the ethical considerations surrounding AI usage are complex and require careful navigation. These issues highlight the need for continued research and careful implementation of these technologies, ensuring they are used responsibly and to benefit everyone.

As we continue to integrate AI and LLMs into various industries and aspects of life, it is crucial to focus on sustainable development. The 2030 agenda for sustainable development adopted by United Nations member states in 2015 outlines peace and prosperity for the people and planet now and in the future. The agenda encompasses 17 Sustainable Development Goals (SDGs) bifurcated into 169 targets addressing economic, social, and environmental aspects. These SDGs recognize that alleviating poverty and other deficiencies must go together with strategies that improve health and education, diminish inequality, and stimulate economic growth. The important aspect of the agenda is that all countries are obliged to enforce SDGs and attempt to balance economic, social, and environmental factors, leading to a sustainable planet (UN SDG, 2015). The SDGs chartered by the United Nations also focus on eight cross-cutting competencies: self-awareness, collaboration, critical thinking, anticipatory, integrated problem-solving, normative, strategic, and systems thinking (UNSDG, 2015). These competencies are correlated with each other, transversal, multifunctional, content dependent, and crucial for achieving SDGs. AI has the potential to contribute significantly to SDGs by optimizing resource management, improving efficiency, and providing innovative solutions to complex problems. However, it is equally essential to ensure that AI’s development and usage align with SDGs, promoting sustainability at every level.

ChatGPT has established itself as a versatile tool in diverse fields, including poetry, essay writing, business communication, research, software development, and testing, as highlighted by [2]. Its applications have spread across numerous sectors, such as medicine and healthcare [6,8,23], scriptwriting for films [27], digital marketing [13], content creation [14], and higher education [17,19]. Scholars have found it beneficial for academic and scientific research writing, hypothesis generation, and resource searches [15,28,29]. It can also assist in translating educational materials into various languages, providing advantages to professionals, students, and academics in media and journalism [30,31].

Nevertheless, recognizing ChatGPT as a co-author has sparked debates [32,33]. Consequently, Kung et al. [34] analyzed ChatGPT’s competence on the United States Medical Licensing Exam (USMLE) in comparison to human clinical experts. The study established that LLMs, like ChatGPT, can be beneficial in medical and clinical assistance. In contrast, Choi et al. [35] evaluated ChatGPT’s ability to grade exams autonomously and found its performance mediocre, akin to a C+ student. Therefore, Katz et al. [24] further assessed GPT-4’s performance against ChatGPT and previous GPT models on the entire Uniform Bar Examination (UBE). The study revealed that GPT-4 is considerably more advanced than LLMs like ChatGPT and can contribute significantly to legal aid.

However, the ethical and credibility aspects of ChatGPT have come under scrutiny [36]. Greengard [37] argued that students’ usage of ChatGPT could lead to reduced creativity, negative impacts on learning culture, erosion of originality, and an over-reliance on technology. Furthermore, Else [38] the challenge of distinguishing between AI-generated and original abstracts, stirring fears of plagiarism and academic integrity issues in higher education [39]. Islam & Islam [40] pointed out the potential benefits and negative impacts of ChatGPT with respect to education, research, personal skill development and society. Therefore, the ethical and practical implications of AI and ChatGPT in education continue to generate debate, and research on their adoption is still in its nascent stages [41,42].

Given the potential of AI in general and specifically ChatGPT in aiding human beings working in different spheres, it will be interesting to explore its applicability in a vital global agenda of humanity. National and individual levels. We are witnessing a rising commitment among nations, organizations, and individuals towards the cause of the pursuit of the United Nations’ Sustainable Development Goals (SDGs) 2030. The transition towards a sharing economy can address the pursuit of SDGs to an extent. Huang [43] discussed the importance of bursting the barriers for the transition towards a sharing economy for achieving SDGs in ASEAN nations. A similar discussion using empirical evidence from developing countries can be found in the work by Tu et al. [44]. Sharing economy benefits for SDGs was also discussed by Sadiq et al. [45] in the context of the transportation industry in Vietnam. The importance and mediating role of energy efficiency on sharing economy benefits and SDGs were investigated by Chien [46]. Apart from the transition towards a sharing economy, knowledge and technology-based innovative solutions are vital for SDG pursuit, and these innovations can even act as an enabler of the sharing economy. The importance of emerging green knowledge management in addressing green innovation and SDGs was discussed by Wang et al. [47]. A recent exploration by Li et al. [48] found a positive impact of knowledge-based dynamic capabilities in national innovation ecosystems on the achievement of SDGs. A study by Chopra et al. [49] attempted to develop a forecasting approach based on big data and attempted to predict the scores of some nations with respect to SDG 9. All these highlight the need for exploration of benefits and assessment of the ability of predictive and decision support systems that can harness large datasets and provide knowledge, insights, and actionable solutions towards the pursuit of SDGs. In this context, it will be vital to be informed of the potential of AI tools in this regard. ChatGPT, being a recent sensation with many promising capabilities, deserves investigation for its potential to contribute to the ongoing pursuit towards SDGs. As Vinuesa et al. [50] emphasized, AI’s expanding influence across various sectors highlights the need to understand its implications for progress toward the SDGs. Singh et al. [51] explored the bibliometric patterns in different SDGs related to the contribution of AI and identified useful and potential AI techniques in each SDG. However, to the best of our knowledge, exploration of the potential of ChatGPT for addressing SDGs has not been attempted till now. This gap is attempted to be addressed in this work. Such an exploration will benefit not only the ones working voluntarily towards the pursuit of SDGs but the entire humanity.

To evaluate AI and ChatGPT’s understanding and literacy about SDGs, this study has chosen two widely used sustainability assessment instruments ‐ the SULITEST (Sustainability Literacy Test) and the UN SDG Fitness Test. These tools will help evaluate ChatGPT’s comprehension of the SDGs at different levels, providing valuable insights into its potential role in promoting sustainable development. As assessed by these tests, ChatGPT’s knowledge and understanding of the SDGs will help determine its potential to aid decision-making and provide necessary information for sustainable practices. While the primary decision-making responsibilities should remain with human stakeholders, organizations can leverage ChatGPT’s knowledge to make informed decisions about their sustainability strategies, such as identifying areas of their operations that most impact the SDGs. For instance, ChatGPT can provide insights into designing environmentally friendly products and services or promoting social equality in hiring and promotion practices. Other stakeholders, like UN bodies, including UNESCO, can utilize ChatGPT to develop content for various short-term and long-term training programs, transforming interested individuals from different fields into sustainability advocates.

In light of recent events like the COVID-19 pandemic, AI LLM bots’ abilities can be utilized for preparedness and early mitigation of potential future pandemic incidences. Such applications can immensely contribute towards SDG 3 (Good Health and Well-being) and other disaster aversion planning and mitigation, impacting a multitude of SDGs and targets.

In addition to SULITEST and SDG Fitness Test, other instruments are available for assessing SDG knowledge and literacy. These include:

- The Global Schools Program’s SDG Test: This test, developed by the UN Sustainable Development Solutions Network’s Youth Initiative, is designed for schools worldwide to assess the understanding of SDGs among students.
- The SDG Academy’s Quizzes: The SDG Academy offers a variety of online courses on the SDGs, each of which includes quizzes and assessments to test learners’ understanding of the specific goals and targets.
- The Bertelsmann Stiftung and Sustainable Development Solutions Network (SDSN) SDG Index: Although this is not a test per se, the SDG Index assesses countries’ progress towards the SDGs and provides a detailed analysis of each goal.

The reasons for choosing SULITEST and SDG Fitness Test over these other tools are -

For SULITEST, it was the first internationally recognized tool to measure knowledge about sustainable development and the SDGs. It has been administered to over 100,000 individuals in over 60 countries, showcasing its global acceptance and credibility. Furthermore, it covers a comprehensive range of topics related to sustainability and the SDGs, making it a holistic tool for this research. On the other hand, the SDG Fitness Test is directly linked to the United Nations, giving it inherent credibility. It is designed to test knowledge of the SDGs and their targets, aligning perfectly with the objectives of this study. The tool’s comprehensive nature, covering all 17 SDGs, ensures a thorough evaluation of AI’s literacy and understanding of the goals.

Therefore, while other tests exist, these two tools’ comprehensive scope and high credibility make them particularly suited for this research. Until now, no research has utilized the SULITEST and SDG Fitness Test to evaluate the SDG core competencies of AI chatbots like ChatGPT. Therefore, the research aims to explore the potential of a Large Language Model like ChatGPT in the United Nations’ Sustainable Development Goals domain that could demonstrate their sustainability literacy, knowledge, and awareness. The study aims to answer specifically the following research questions.

RQ 1: How literate is ChatGPT regarding SDGs?
RQ2: Does SDG literacy indicate that ChatGPT is SDG intelligent?
 2.1) How much is ChatGPT aware of each core competency of SDGs?
 2.2) How much is ChatGPT aware of each of the 17 SDGs?

## 2. Literature review

### 2.1 ChatGPT

Table 1 highlights the proliferation of ChatGPT in research in recent times and the contexts in which studies have been conducted. ChatGPT, a creation of OpenAI, leverages artificial intelligence (AI) technology to function as a natural language processing tool. The AI-driven tool enables users to engage in human-like conversations and assists them in finding information across various topics, thus assisting in composing emails, essays, and code. Developed based on AI and web-sourced data, ChatGPT showcases various capabilities, including composing essays, creating poetry, solving coding issues, and explaining complex concepts. OpenAI introduced ChatGPT to the public in November 2022.

**Table 1:** Studies investigating the application of ChatGPT in various contexts.

Author	Study Context	Observed Components
Katz, D. M., Bommarito, M. J., Gao, S. & Arredondo, P., [24]	Performance of GPT-4 against prior generations of GPT on the entire Uniform Bar Examination	Performance of GPT-4 for Bar Exam, natural language processing, machine learning, artificial intelligence
Wang, L. et al. [52]	Effectiveness of Large language models such as ChatGPT & GPT-4 in understanding and evaluating human discourse and document-level translation	Performance of ChatGPT & GPT-4
Ali, R. et al. [53]	Performance of ChatGPT and GPT-4 on Neurosurgery Written Board Examinations	Assessed the performance of ChatGPT and GPT-4 on a 500-question mock neurosurgical written boards exam of the American Board of Neurological Surgery.
Teebagy, S. [54]	Performance of ChatGPT vs. ChatGPT-3.5 on the Ophthalmology Knowledge Assessment Program (OKAP)	AI and the performance of ChatGPT and GPT-4 on tested on 180 OKAP practice questions covering various categories of ophthalmology
Kung, T. et al. [34]	Performance of ChatGPT on the United States Medical Licensing Exam (USMLE)	Measured AI medical knowledge through the Performance of ChatGPT in USMLE compared to expert human clinicians. The result showed that ChatGPT performed at or near the passing threshold of 60% accuracy.
Blanco-Gonzalez, A. et al. [55]	Role AI in reforming drug discovery	The study examined the challenges, efficiency, and limitations of AI in reforming drug exploration.
Biswas, S.S. [8]	Role of ChatGPT in public health in individual and community health decision	Possibility of ChatGPT in supporting communities to take informed health decisions. The study also examined the challenges and limitations of ChatGPT in public health.
Graham, F. [32]	How AI LLMs can be a threat to scientific research practices and values	It delved into the convolutions in research in terms of credit, attribution, and misinformation
Else, H. [38]	The ChatGPT written abstracts are very tough to be identified by the research scientists.	The study evaluated the ethical aspects of identifying AI-written vs. human research articles.
Stokel-Walker, C. & Van Noorden, R. [56]	The researchers are alarmed about the usage of AI in scientific research	The study evaluated the effectiveness of AI, ML, and publishing.
Cascella, M. et al. [23]	The practicability of ChatGPT in healthcare	Possible Benefits and ethical Practicability of ChatGPT in the clinical research situation
Dowling, M. & Lucey, B. [15]	ChatGPT for financial research.	Finance research experts analyzed chatGPT-generated output and found that ChatGPT lags in research synthesis.
Dwivedi, Y. K. et al. [2]	Examined the possibilities of ChatGPT in various sectors	Application of ChatGPT for marketing, banking, hospitality supply chain, tourism, and ethics.
Tlili, A. et al. [57]	Usage of ChatGPT for education	Analyzing the impact of the usage of ChatGPT for education among the early adopters of technology.
Gupta, R. et al. [58]	Enhancing research in cosmetic plastic surgery using ChatGPT.	Accuracy of ChatGPT in doing a systematic review for cosmetic surgery
Carvalho, I. & Ivanov, S. [59]	Evaluating the risks, benefits and disruption AI and LLMs can bring to the tourism industry	Impact of ChatGPT on the Functioning of Tourism and Customer Services
Dubin, J. A. et al. [60]	Comparison of Google web search results of queries vs. ChatGPT for Joint Arthroplasty	10 FAQs were asked to both Google and ChatGPT, and their answers were compared. Google brings more ads, whereas ChatGPT responds by citing government websites and PubMed.
Choi, J. H., Hickman, K. E., Monahan, A. & Schwarcz, D. [35]	Accuracy and performance of ChatGPT for law exams	ChatGPT performs average in the real exams of law school.
Holzinger, A. et al. [61]	Usage of AI in biotechnology to achieve SDGs.	Possible challenges AI can solve in removing hurdles and attaining SDGs.

Since its launch, ChatGPT has made significant strides, garnering attention from various industries and application areas, as cited in Table 1. It is capable of providing users with information on a broad array of subjects, resulting in applications across sectors such as healthcare [6–9,23,29], education [10,11,31,57,62], banking as smart service technologies [2,15,41], tourism [59], and sales and marketing [2,13].

In healthcare and clinical research, Cascella et al. [23] found ChatGPT useful, where it can support clinical practice, medication production, misuse, and reasoning about public health topics. Users are increasingly responsible for raising awareness about the capabilities of ChatGPT and ethical usage. Sallam [9] identified several benefits of ChatGPT, including enhancing scientific writing, utility in healthcare research, streamlining workflow, saving costs, aiding documentation, personalizing medicine, and improving personalized learning. However, ethical, copyright, transparency, and legal issues were raised as potential concerns. For education, ChatGPT represents a significant advancement in AI technologies, aiding in the creation of educational materials, motivating students to tackle complex topics, and developing immersive learning experiences [10]. Despite its potential, Kasneci et al. [10] emphasized that instructors are still required to guide learners to use ChatGPT appropriately, given its status as an emerging technology that lacks critical analysis.

ChatGPT can also be useful in medical education, helping students find relevant information on complex infections, diseases, or living organisms, thereby assisting them in composing literature and initial reports [11]. A limitation of ChatGPT, however, is its inability to provide in-depth and highly relevant information; it also risks encouraging cheating among students without proper supervision. Moreover, ethical concerns, medico-legal and copyright issues, lack of creative thinking, methodological biases, and inaccuracies are also associated with ChatGPT [2,11]. Despite these limitations, ChatGPT can revolutionize sectors such as tourism education and research by generating research papers, suggesting the potential to replace human researchers [63].

In the educational sphere, Rospigliosi [62] suggested that ChatGPT should embody the three essential characteristics of an integrated learning environment: appropriability, evocativeness, and integration. By facilitating experiential learning, ChatGPT can help students explore various methods and techniques for problem-solving and goal attainment [64]. Students who prefer immersive, hands-on learning will likely benefit from using ChatGPT as a learning tool [41]. Universities are encouraged to incorporate generative language models into their teaching pedagogies [10,63]. However, educators and policymakers must continue assessing the benefits and limitations of ChatGPT as the technology evolves. The journey towards fully realizing the potential of AI in various sectors is ongoing, and ChatGPT represents a significant milestone in this journey.

In a study by Raman et al. [65] examining the early attention to ChatGPT research using the Altmetric Attention Score (AAS), findings indicate that the United States, Japan, and the United Kingdom are the leading countries in publishing high-impact ChatGPT research, predominantly in journals such as Nature and Science. The primary fields of research (FoR) associated with ChatGPT publications are ’information and computing sciences’ and ’biomedical and clinical sciences,’ with key thematic clusters involving ChatGPT’s role in medical writing and scientific publishing, and scientists emerging as the major user category interested in ChatGPT research.

Finally, ChatGPT can be a valuable tool for content developers in sales and marketing due to its impressive knowledge across various topics and ability to produce high-quality, factually accurate writing [31]. However, its limitations in critical and creative thinking are also apparent. In the banking sector, as a part of smart service technologies, ChatGPT can assist with customer inquiries and automate routine tasks, which leads to improved efficiency and customer satisfaction [2,15,41]. ChatGPT can play a significant role in tourism by providing in-depth information about locations, local customs, attractions, and travel advice, thereby enriching the user’s travel experience [59].

Despite its wide range of applications, there is an ongoing need for careful monitoring and management of ChatGPT’s use. Concerns around honesty, privacy, misleading information, and manipulation have been raised, necessitating caution and further investigation into user experiences [57], as mentioned in Table 1.

### 2.2 SULITEST ‐ Sustainability Literacy Test

The SULITEST ‐ Sustainability Literacy Test aims to enhance sustainability literacy. It offers a globally recognized and locally relevant tool for higher education institutions, businesses, and other organizations globally. According to Decamps et al. [66], The SULITEST test is a freely accessible online evaluation tool developed by Education for Sustainable Development (ESD) and the Principles for Responsible Management Education (PRME). Higher education institutions primarily use it to measure sustainability literacy and awareness [67].

While the SULITEST has been widely administered (approximately 160,000 individuals across 63 different countries have taken the test), a systematic examination of the test’s results or data is lacking. Kuehl et al. [68] conducted a confirmatory and exploratory factor analysis on SULITEST, the standard method for identifying latent factors in observable data. Although SULITEST was designed to assess knowledge across four themes, the data did not support this framework, suggesting policymakers and educators should exercise caution when using it to evaluate sustainability understanding [68]. Melles and Paixao-Barradas [69] critiqued the test’s lack of a design module. Their study underscored the efficacy of including such a module in the SULITEST structure. Meanwhile, Nolan et al. [70] evaluated the effectiveness of SULITEST by administering it to over 300 students using the ’describe, interpret, evaluate, plan’ framework. They found that students were generally aware of topics like the circular economy, child labour, pollution, equality, and clean energy.

**Fig 1:**
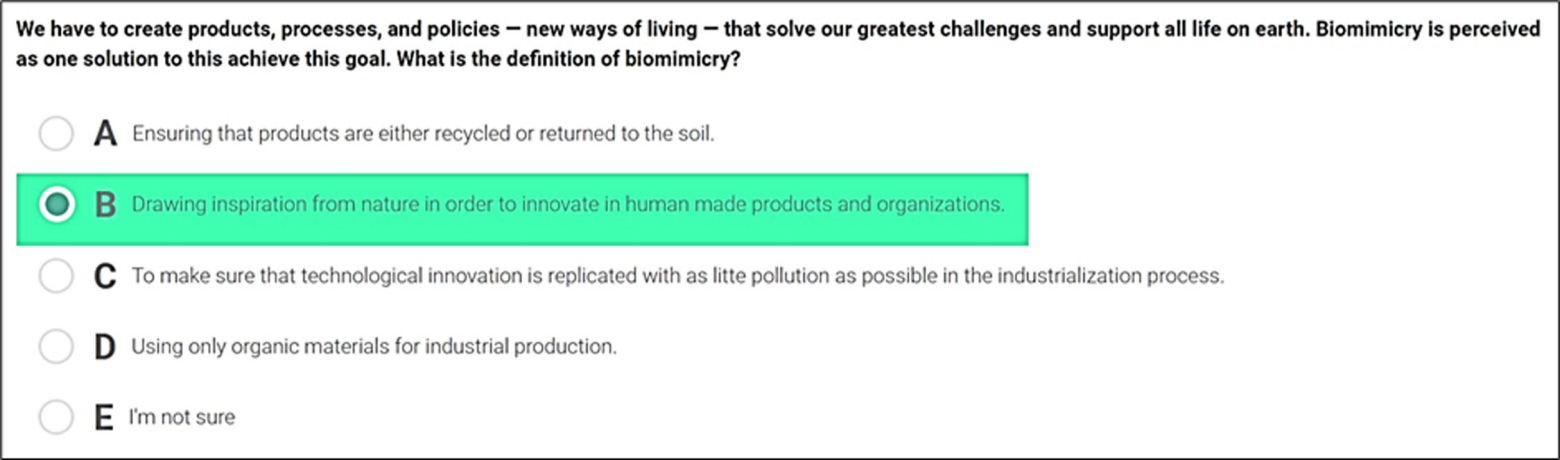
Sample question from SULITEST.

A sample question from SULITEST is shown in Fig 1. It utilizes a multiple-choice questionnaire format that can be accessed online. Each test features a minimum of 30 randomly selected questions from a comprehensive question bank divided into distinct modules. This format was chosen for its user-friendly, adaptable nature and universal accessibility. Each SULITEST session includes at least thirty questions from the Universal Core International Module, addressing many globally relevant issues. This allows organizations and individuals to measure their performance on an international scale. These 30 questions are often supplemented with 20 "local" questions from Specialized Local Modules, addressing issues and challenges specific to regional or country-specific contexts.

Following each session, respondents can take an anonymous survey regarding their socio-demographic characteristics, interest in sustainability issues, and experience with Education for Sustainable Development (ESD). The survey aims to gather research data on the respondent’s background and engagement with sustainable development.

### 2.3 UN SDG fitness test

UN SDG: Learn is a United Nations initiative to provide individuals and organizations with relevant, well-curated learning resources addressing sustainable development issues. The UN SDG Learn collaboratively offers the SDG Fitness Test, the United Nations Educational, Scientific, and Cultural Organization (UNESCO), and the United Nations Institute for Training and Research (UNITAR). It is a self-assessment test that presents a viewpoint on the user’s current knowledge and skills regarding eight key competencies pertinent to achieving SDGs [71]. Users must register on the site to use the SDG Fitness Test, providing their email and other pertinent information. Subsequently, they must select up to three ’mental models,’ representing the roles they perceive themselves playing in their journey to learn about the SDGs (e.g., businessperson, citizen, thinker, policymaker, and project manager, among others).

**Fig 2:**
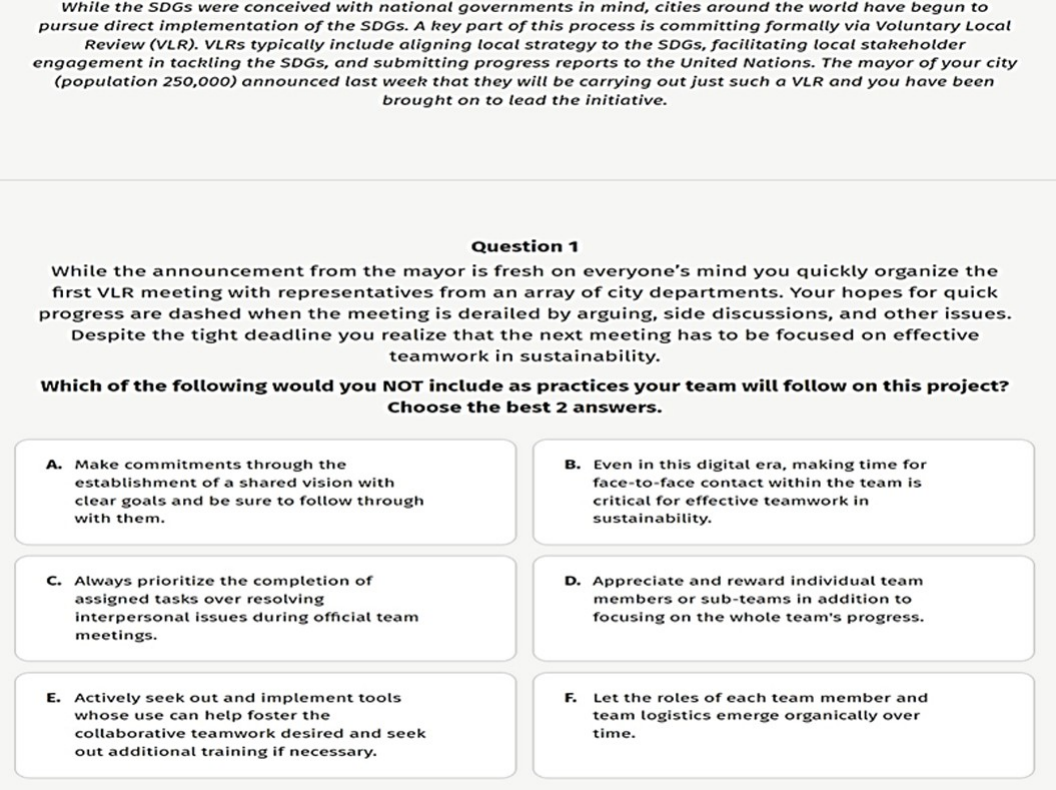
Sample question from SDG fitness test.

Further, users must choose up to five SDGs they are particularly interested in before completing the registration process. SDG Fitness Test helps an individual understand the level of awareness of SDG core competencies by immersing in real-life scenarios. SDG Fitness assessment consists of 24 questions formulated around four scenarios. Each scenario puts the learner in a different role in either government, non-profit, private sector, or as an individual citizen. The scenarios represent real-life situations that may happen worldwide on the path to a more sustainable and resilient planet. Each scenario was entered individually, followed by the question in a multiple-choice format. The test evaluates sustainability competencies in eight areas: Systems thinking, Anticipatory, Normative, Strategic, Collaboration, Critical thinking, Self-awareness, and Integrated problem-solving [72]. A sample question from the SDG Fitness Test is shown in Fig 2 below.

## 3. Methodology

**Fig 3:**
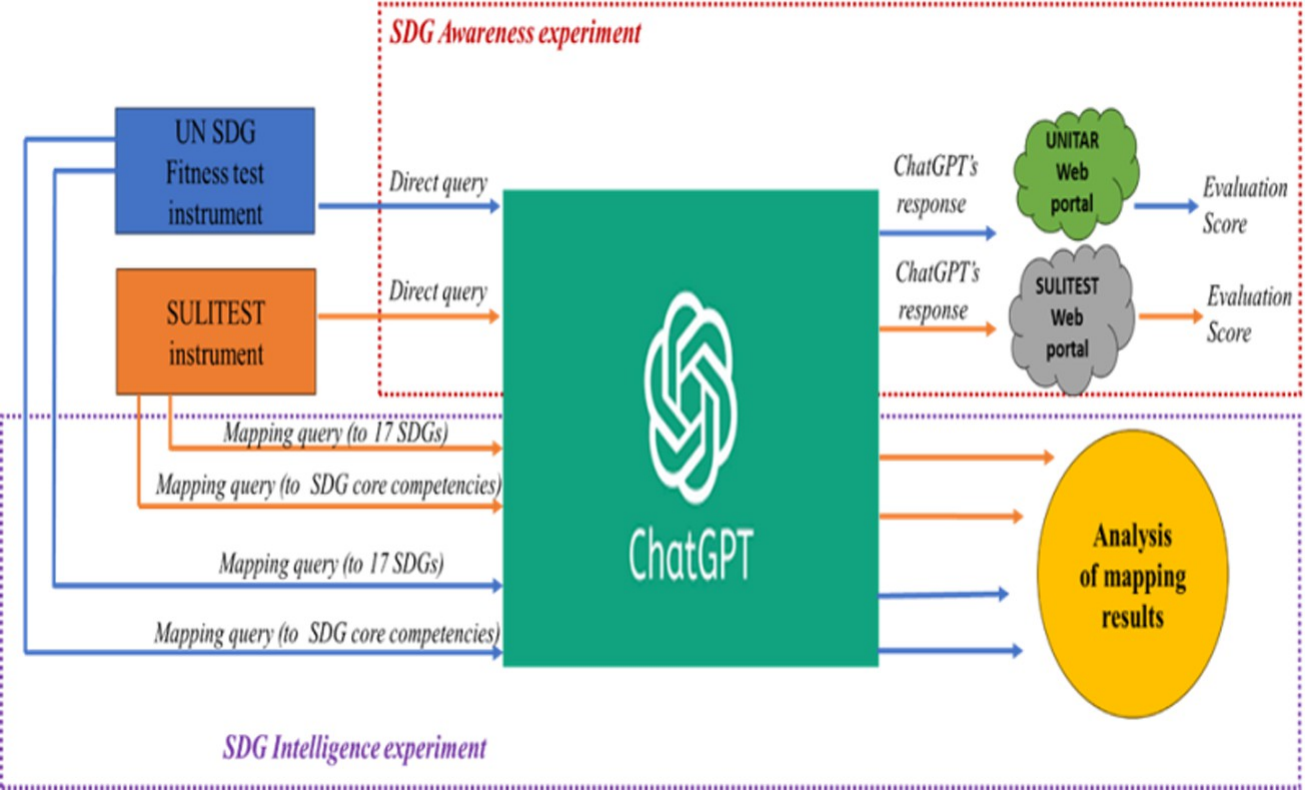
Schema of the methodology used for this study.

The procedure for this study is shown in Fig 3. In this study, we utilized the GPT-4 iteration of ChatGPT to measure ChatGPT’s Sustainable Development Goals (SDGs) awareness and intelligence. As the SDG Fitness test and SULITEST are designed to determine the SDG awareness of individuals, these instruments are used to address our first research question (RQ1), i.e., to determine the level of awareness of ChatGPT about SDGs. Test. Queries in both the instruments were fed directly to ChatGPT. Conversations were reset after each inquiry to avoid any residual effects. Each of the recorded responses was entered into the UNITAR web portal and SULITEST web portal (as it is required by humans taking respective tests). The evaluation results provided by the respective portals are recorded for analysis. If the scores are sufficiently high, a candidate (here ChatGPT) can be regarded as SDG literate. But to treat any candidate as SDG intelligent, deep examination is required.

Apart from providing overall scores related to cross-cutting competencies of SDGs based on the responses, the SDG Fitness test provides graded performance indicators (grades being introductory, intermediate and advanced) related to each competency (some of the competencies like self-awareness, critical thinking competency, etc., are directly related to intelligence). The definition and details of these competencies can be found in section 5. This clearly implies that a possible mapping exists between questions in the SDG Fitness test instruments and these competencies. However, this mapping is not available in the public domain. If the instruments (both the SDG Fitness instrument and the SULITEST instrument) are sufficiently mapped to all the SDG competencies, then only a candidate’s intelligence can be assessed flawlessly.

Also, as the subject matter of test instruments concerns SDGs, a possible mapping exists between test questions and different SDGs. Understanding of a candidate about multiple SDGs and their possible direct or profound interrelatedness as well as indirect or subtle interrelatedness, etc., that are often required to solve problems related to multiple SDGs are assessed via the test instruments. This can also be treated as an assessment of intelligence if all the SDGs are sufficiently mapped to different SDGs (possibly all the SDGs). Thus, our major observation is that the SDG Fitness test instrument and SULITEST instrument can be potential instruments for the assessment of SDG intelligence if the instrument questions are sufficiently mapped to (i) all or most of the cross-cutting SDG competencies and (ii) all or most of the different SDG types. Thus, the second research question (RQ2) is divided into two sub-questions.

The initial one examines SDG competency mapping of test instruments. This can be evaluated by correlating questions from the SDG Fitness Test and SULITEST to various SDG competencies. The objective is to identify whether these tools favour questions related to a particular set of competencies or if they cover a broad range. If the questionnaires are comprehensive, these can effectively highlight the candidates’ (in this case, ChatGPT’s) competencies. Otherwise, there might be a risk of bias or imbalance in SDG competency representation. The secondary sub-question explores instruments’ mapping to different SDG types. This can be assessed by correlating questions from both tests to different SDG categories. The aim is similar: to ascertain whether the tests are skewed towards particular SDGs or if they represent all SDGs adequately. If the latter, the tests can effectively evaluate the candidates’ (ChatGPT’s) SDG awareness. However, they might not accurately reflect SDG awareness if they lean towards certain SDGs. Thus, for RQ2, it is necessary to link different questions to SDG competencies and types.

However, we currently lack such a mapping. Gathering expert opinions and reaching a consensus on this matter can be time-consuming. As a workaround, we conducted another experiment using ChatGPT. We input a query to map the SDG Fitness Test and SULITEST questions to various competencies. We asked ChatGPT: *"Which of the eight overarching sustainability skills—Self-Awareness*, *Collaboration*, *Critical Thinking*, *Anticipatory*, *Integrated Problem Solving*, *Normative*, *Strategic*, *and Systems Thinking—does this question address*?*"*

In a subsequent experiment, we requested ChatGPT to map the questions from both tests to the 17 SDGs. We posed this question to ChatGPT: *"Which SDGs does the above question align with*?*"*

## 4. Results

### 4.1 ChatGPT performance on SDG fitness test

**Fig 4:**
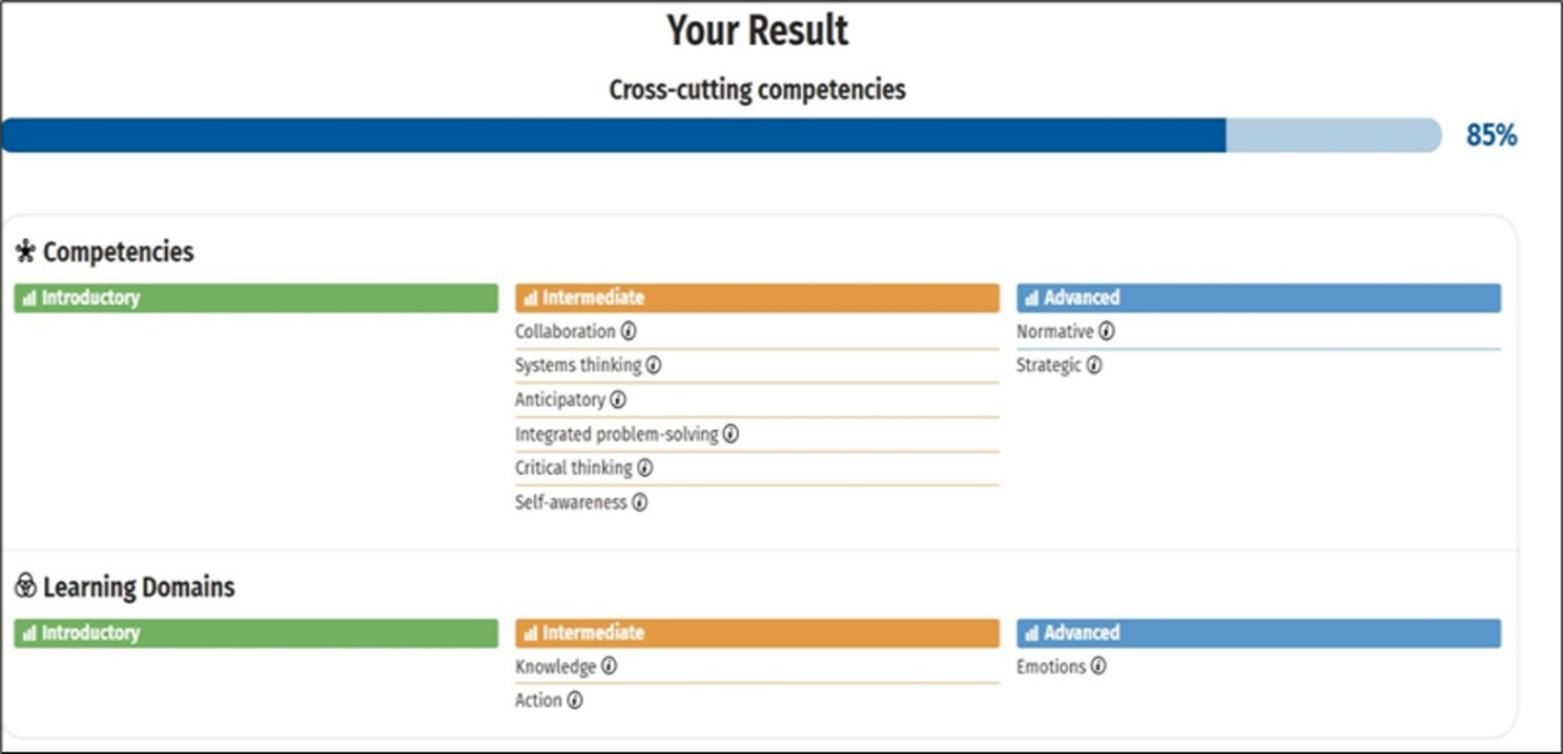
Summary results of ChatGPT performance on SDG fitness test.

Firstly, upon evaluating ChatGPT’s performance on the SDG Fitness Test, ChatGPT scored an overall 85%. Considering this high score, ChatGPT can be regarded as SDG literate. The overall assessment score of ChatGPT on the SDG Fitness Test can be found in Fig 4.

Regarding scores at the competency level, for two competencies, namely Normative and Strategic competencies, ChatGPT scored at the ’advanced’ level. For six competencies like ’Collaboration,’ ’Systems thinking,’ ’Anticipatory,’ ’Integrated problem-solving,’ ’Critical thinking,’ and ’Self-awareness,’ ChatGPT scored at the intermediate level only. Regarding learning domains, ChatGPT scores at an advanced level for the ’Emotions’ domain and only at the intermediate level for the ’Knowledge’ and ’Action’ domains.

### 4.2 ChatGPT performance on SULITEST

**Fig 5:**
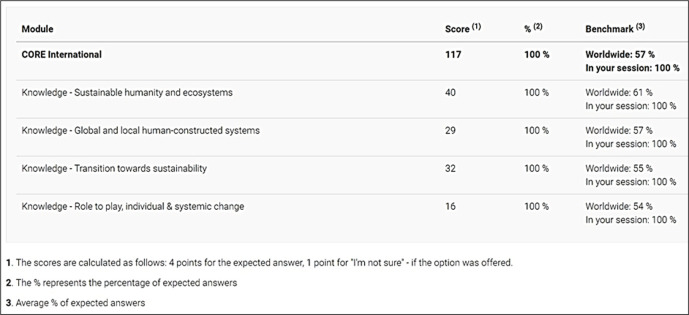
Summary results of ChatGPT performance on SULITEST.

ChatGPT scored 117 out of 120 (97.5%) on SULITEST (Fig 5). Such a high score again underlines the high SDG literacy of ChatGPT. SULITEST questions do not have provisions for evaluating SDG competencies. Instead, it maps questions to the domain ’Knowledge’ only. Various sub-domains within the knowledge domain are ’Sustainable humanity and ecosystems’, ’Global and local human-constructed systems,’ ’Transition towards sustainability,’ and ’Role to play, individual & systemic change.’ Whether ChatGPT is SDG intelligent can only be known if the mappings of test questions to SDG competencies and mappings are known. These are explored in sections 4.3.2 and 4.3.4.

### 4.3 Analysis of SDG intelligence

While the previous questions only determined whether ChatGPT is literate in SDGs (and our findings confirm that it is), to discern if ChatGPT possesses SDG intelligence, some competencies that can be regarded as traits of intelligence should be suitably attributed to ChatGPT. As already mentioned, the SDG fitness test provides graded performance indicators related to cross-cutting competencies. These competencies qualify as traits of intelligence. Can SDG fitness and SULI test instruments determine whether ChatGPT or LLMs are SDG intelligent? These tests can determine SDG intelligence if and only if all the competencies are sufficiently and equitably covered in the test instrument. Apart from this, to reinforce SDG intelligence, the test instruments require a sufficient and equitable representation of questions. Thus, to determine the SDG intelligence, we must examine if the questionnaire adequately represents various competencies and covers all the SDGs equitably. This can be achieved by evaluating the SDG competency of each question and categorizing each question under different types of SDGs. How can this be done? Expert opinion-based mapping of all the questions in both test instruments to different cross-cutting competencies and different SDG types is an option. However, it is a very time-consuming, tedious and expensive option. Is there any pragmatic alternative for such a mapping exercise? LLMs, being powered with NLP techniques for text tagging or classification, offer such a possibility (though not without limitations). Thus, we conducted this analysis on ChatGPT and elaborated on it in the upcoming subsections.

#### 4.3.1 Mapping of SDG fitness test questions to SDG competencies

UNESCO has identified eight key competencies: self-awareness, collaboration, critical thinking, anticipatory, integrated problem-solving, normative, strategic, and systems thinking [73]. Competencies represent the attributes individuals need to self-organize and act in various situations and contexts [74]. As competencies encompass cognitive, affective, volitional, and motivational elements, they mix knowledge, capacities, skills, motives, and attitudes [74]. The eight competencies in the 2030 Agenda for Sustainable Development are crucial to advancing sustainable development [75,76]. These key sustainable competencies equip individuals to tackle today’s complex challenges [72]. All 17 SDGs are relevant to these eight competencies.

The results (Table 2) demonstrate that ChatGPT mapped all eight key competencies across 24 questions. The competency of collaboration received the most coverage, with ChatGPT associating it with all 24 SDG Learn Fitness test questions. The competency of collaboration encompasses the capacity to learn from others, respect and appreciate others’ needs, perspectives, and actions (empathy), understand and relate to others (empathetic leadership), manage group conflicts, and facilitate collaborative and participative problem-solving [75,76]. The least coverage was given to self-awareness, associated with four questions. Self-awareness involves the ability to reflect on one’s role in the local and global community, continually assess and motivate one’s actions, and manage one’s emotions and desires [75,76]. Although ChatGPT broadly mapped the eight key competencies across all 24 questions, it remains unclear which part of the question refers to a specific competency.

**Table 2:** Mapping of SDG fitness test questions to SDG core competencies.

Q. No.	Self -Awareness	Collaboration	Critical Thinking	Anticipatory	Integrated Problem Solving	Normative	Strategic	Systems Thinking
1		x	x		x		x	x
2		x		x	x		x	x
3		x		x	x		x	x
4		x	x		x		x	x
5		x	x	x		x		x
6	x	x	x	x	x	x	x	x
7	x	x	x		x	x	x	
8		x		x	x		x	x
9		x	x	x	x		x	x
10	x	x	x				x	x
11		x			x	x		x
12		x	x	x	x		x	x
13		x	x	x	x	x		x
14		x		x	x	x		
15		x	x	x	x	x	x	x
16		x		x	x		x	x
17		x		x		x		x
18		x	x	x	x		x	x
19		x	x			x	x	x
20	x	x	x	x	x	x	x	x
21		x	x		x	x	x	x
22		x						x
23		x		x	x		x	x
24		x	x	x	x		x	x
**Total**	**4**	**24**	**15**	**16**	**19**	**11**	**18**	**22**

Furthermore, issues were reported with repeated iterations. ChatGPT assigned different SDGs to the same question simultaneously for the same user profile and mental models. This inconsistency is concerning, as test-retest reliability is crucial to ascertain the dependability of a measurement tool [77].

#### 4.3.2 Mapping of SULITEST questions to SDG competencies

Upon feeding the query to map SDG competencies to the SULITEST questionnaire, it is found that competencies like critical thinking competency, anticipatory competency, normative competency, systems thinking competency, and integrated problem-solving competency are mapped to 28, 26, 23, 19, and 18 questions respectively, whereas competencies like self-awareness competency, collaboration competency, and strategic competency are mapped to 6, 4 and 13 questions (Table 3).

**Table 3:** Mapping of SULITEST questions to SDG competencies.

Q. No.	Self -Awareness	Collaboration	Critical Thinking	Anticipatory	Integrated Problem Solving	Normative	Strategic	Systems Thinking
1			x	x	x		x	x
2	x	x	x			x	x	
3				x	x	x	x	x
4			x	x	x	x		x
5	x	x	x	x	x	x	x	x
6			x	x	x	x		x
7	x		x			x	x	x
8			x	x	x	x	x	x
9			x	x	x		x	x
10	x		x	x	x	x		x
11			x	x	x			x
12			x	x	x			x
13	x		x	x				x
14			x	x	x	x		x
15			x	x	x	x		x
16			x	x	x	x		x
17			x			x	x	x
18			x	x	x	x		x
19			x	x	x	x		x
20			x	x	x	x		x
21			x	x	x	x		x
22	x	x	x	x				x
23		x		x			x	x
24			x	x	x	x	x	x
25			x	x		x		x
26			x	x		x		x
27			x	x		x		x
28			x			x	x	
29			x	x		x		x
30			x	x		x	x	
**Total**	**6**	**4**	**28**	**26**	**18**	**23**	**13**	**19**

While questions 2, 5, 7, 10, 13, and 22 are mapped to Self-awareness competency, questions 2, 5, 22, and 23 are mapped to Collaboration competency.

#### 4.3.3 Mapping of SDG fitness test questions to 17 SDGs

ChatGPT’s overall score on the SDG Fitness Test was 85%, indicating substantial SDG literacy. However, concerns arise regarding the mapping and coverage of SDGs across the 24 questions by ChatGPT. While ChatGPT does map each question to SDGs (Table 4), it is unclear which aspect of the question corresponds to a specific SDG. Also, SDGs 6, 12, and 15 received inadequate coverage from ChatGPT.

**Table 4:** Mapping of SDG fitness test questions to 17 SDGs.

SDG/Q.NO.	1	2	3	4	5	6	7	8	9	10	11	12	13	14	15	16	17	18	19	20	21	22	23	24
**SDG 1:** No Poverty							x		x		x			x							x			x
**SDG 2:** Zero Hunger							x	x	x		x													
**SDG 3:** Good Health & Well being	x						x	x			x						x		x		x			x
**SDG 4:** Quality Education	x						x							x			x		x	x				x
**SDG 5:** Gender Equality	x								x	x			x					x						
**SDG 6:** Clean Water & Sanitation																x								
**SDG 7:** Affordable & Clean Energy													x	x	x	x	x	x						
**SDG 8:** Decent work & economic growth							x		x		x		x	x										x
**SDG 9:** Industry Innovations & Infrastructure								x				x												
**SDG 10:** Reduced Inequalities										x		x				x			x	x				x
**SDG 11:** Sustainable Cities & Innovation	x		x	x	x	x									x				x	x	x	x	x	x
**SDG 12:** Responsible Consumption & Production						x																		
**SDG 13:** Climate Action		x			x	x							x	x										
**SDG 14:** Life Below Water																								
**SDG 15:** Life on Earth								x																
**SDG 16:** Peace, Justice & Strong Institutions	x		x	x						x								x	x	x		x	x	x
**SDG 17:** Partnerships for the goals	x	x	x	x	x				x	x	x	x			x	x	x		x	x	x	x	x	x

SDG-6, which emphasizes clean water and sanitation for all, was only associated with question 16 of the SDG Fitness Test. This is worrying, especially considering the SDG Report 2022’s call for immediate action on misuse, poor management, over-extraction, and groundwater contamination. The pandemic has underscored the importance of safely managed drinking water, sanitation, and hygiene services for public health [78].

Similarly, SDG-12, which promotes responsible consumption and production, was linked to question 6. Given that ignorance about sustainable production and consumption contributes to climate change, biodiversity loss, and pollution, the limited coverage of SDG-12 is concerning. SDG-15, focusing on life on land, is another critical area that was inadequately covered, associated with only one question. This is disconcerting, considering the essential role of healthy ecosystems and biological diversity in providing food, water, medicine, shelter, and other material goods [78]. ChatGPT did not associate SDG-14, which stresses life below water, with any question in the SDG Fitness assessment. With the planet’s largest ecosystems—oceans and seas—under threat from human activity, this oversight is a significant gap that needs addressing.

On a brighter note, the SDGs that received the most coverage in the SDG Fitness Test administered to ChatGPT were SDG-17, SDG-11, and SDG-16. SDG-17, focusing on partnerships for the goals, was associated with 18 questions. SDG-11, which centers on sustainable cities and communities, was linked to 12 questions. SDG-16, which advocates for peace, justice, and strong institutions, was associated with ten questions. These findings indicate a high level of awareness by ChatGPT about SDGs 17, 11, and 16.

#### 4.3.4 Mapping of SULITEST questions to 17 SDGs

ChatGPT was able to map each of the 30 questions to different SDGs with a degree of success (Table 5). However, all but three SDGs (SDG 11, 12, and 13) appear underrepresented, being tied to only a handful of questions. SDG 11 pertains to ’sustainable cities and communities’, SDG 12 to ’responsible production and consumption,’ and SDG 13 to ’climate action.’ These SDGs are closely interlinked. SDG 12 is vital for achieving both SDG 11 and SDG 13, while SDGs 12 and 13 are necessary for realizing SDG 11. Although other SDGs are essential for achieving SDGs 13, 12, and 11 and sometimes the other way around, these other SDGs seem underrepresented in the questionnaire. For instance, SDG 3 is crucial for the attainment of SDG 11. SDGs like 1, 2, 4, 6, 7, 8, 9, and 16 are also important for realizing SDG 11. The efficacy of the questionnaire is questionable, given the underrepresentation of these many SDGs. A more detailed discussion on the underrepresentation and its impact on the questionnaire’s ability to assess SDG intelligence can be found in section 5.

**Table 5:** Mapping of SULITEST questions to 17 SDGs.

SDG/Q.NO.	1	2	3	4	5	6	7	8	9	10	11	12	13	14	15	16	17	18	19	20	21	22	23	24	25	26	27	28	29	30
**SDG 1:** No Poverty											x																			
**SDG 2:** Zero Hunger						x					x				x												x			
**SDG 3:** Good Health & Well being			x	x							x					x						x								
**SDG 4:** Quality Education		x			x		x															x							1	
**SDG 5:** Gender Equality																						x								
**SDG 6:** Clean Water & Sanitation				x							x								x		x						x			
**SDG 7:** Affordable & Clean Energy															x										x					
**SDG 8:** Decent work & economic growth																				x										
**SDG 9:** Industry Innovations & Infrastructure	x		x					x													x				x	x				
**SDG 10:** Reduced Inequalities																x				x		x								
**SDG 11:** Sustainable Cities & Innovation	x	x	x			x	x			x	x								x	x					x					
**SDG 12:** Responsible Consumption & Production	x	x		x	x	x		x		x					x		x		x	x	x		x	x			x			
**SDG 13:** Climate Action	x		x			x		x			x	x	x	x					x	x			x		x	x				1
**SDG 14:** Life Below Water	x			x									x	x					x					x						
**SDG 15:** Life on Earth	x			x	x														x	x							x			
**SDG 16:** Peace, Justice & Strong Institutions		x															x					x								
**SDG 17:** Partnerships for the goals		x														x	x						x	x		x				1

## 5. Discussions

The Results section reported that ChatGPT’s overall score on the SDG Fitness Test was 85%, indicating a significant level of SDG literacy. However, it should be considered to gain insights into ChatGPT’s SDG intelligence, its performance, and the mapping of questions in the SDG Fitness Test & SULITEST towards SDG competencies and SDG types. The degree of ChatGPT’s intelligence can largely be attributed to competencies such as critical thinking, systems thinking, and self-awareness. However, ChatGPT only scored at an intermediate level in these areas, implying that it cannot be considered fully SDG intelligent. Another factor to consider is the strength of mapping the test questions to these SDG competencies. If the questions were not properly aligned with these competencies, the instrument might not be sufficient to assess these competencies in a candidate (in this case, ChatGPT).

### 5.1 Mapping of SDG fitness test to SDG competencies

As already mentioned, the six competencies in which ChatGPT scored at the intermediate level and their representation through mapping with 24 questions in the SDG Fitness Test are given in Table 6.

**Table 6:** Mapping of SDG fitness test to SDG competencies.

Competency	No. of questions mapped (X)	% of mapping (X/24 *100%)
Self-awareness	4	16.6%
Collaboration	24	100%
Critical thinking	15	62.5%
Anticipatory	16	66.6%
Integrated-problem solving	19	79.2%
Systems thinking	22	91.6%

In assessing ChatGPT’s ability to map various questions to SDG competencies, we find that all competencies, apart from self-awareness, are well represented in the SDG Fitness instrument. We’ve set 50% as the threshold score, below which competency is considered underrepresented or under-mapped. Therefore, aside from the ’self-awareness’ competency, the SDG Fitness instrument effectively evaluates all other competencies. Although the questionnaire may not perfectly reflect all competencies, the sufficient mapping of competencies corroborates the hypothesis that ChatGPT’s SDG intelligence is not fully up to par. We’ll discuss the intermediate-level competencies of ChatGPT in more detail.

**Critical Thinking:** Lai [79] defines critical thinking as the ability to analyze arguments, make inferences using inductive or deductive reasoning, judge or evaluate, and make decisions or solve problems. Despite having access to general and domain-specific SDG-related text, ChatGPT’s intermediate performance might be due to its lack of capability to make inductive or deductive reasoning inferences. OpenAI might consider integrating critical thinking capabilities into ChatGPT’s inference mechanism.

**Self-awareness:** Self-awareness is the capacity to become the object of one’s attention [80]. It is unclear how self-aware ChatGPT is about its abilities and other details. Although it may know its capabilities in different areas, it might not be able to disclose certain details due to manufacturer-imposed restrictions. It is also uncertain whether it can consider itself as an object of its attention. These considerations require a deeper examination and may provide useful insights for OpenAI.

**Collaborative Competency:** According to Lai [81], collaboration is the mutual engagement of participants in a coordinated effort to solve a problem. However, it is debatable whether ChatGPT requires collaboration like humans. If required, with whom should it collaborate? With other AI models, or should it generate queries to gather information from search engines as a form of self-training or self-updating? These questions may be worth considering for OpenAI.

**Anticipatory Competency:** Rhodes and Ross [82] defined anticipatory competency as the capacity to continuously develop and apply knowledge through a structured approach to anticipate changing scenarios over time. While ChatGPT might be useful in developing plans or solving strategic problems when clearly stated, it doesn’t seem to be able to anticipate changing scenarios unless explicitly stated in the text. Its intermediate score in anticipatory competency might be due to the textual clarity of potential changes in the SDG Fitness questionnaire.

**Integrated-Problem Solving:** UNESCO (2017) describes this competency as applying different problem-solving frameworks to complex sustainability problems and developing feasible, inclusive, and equitable solution options promoting sustainable development. This capability seems more related to the subject domain ’Sustainability’ but requires the application of multidisciplinary knowledge. ChatGPT might have information from various disciplines but may lack the overarching skill of integrating and applying knowledge. Also, OpenAI has indicated that ChatGPT is prone to hallucinations, which might lead to misinterpretations and the application of incorrect problem-solving approaches.

**Systems Thinking Competency:** This refers to the ability to recognize and understand relationships, analyze complex systems, consider how systems are embedded within different domains and scales, and deal with uncertainty [72]. While ChatGPT might understand different systems from its training data, its ability to identify new systems and understand their interactions and relationships is unclear. This might contribute to its intermediate score in the SDG Fitness Test. Given this, SDG researchers and policymakers might want to exercise caution in taking advice from ChatGPT for SDG-related decisions, as its competency in systems thinking, among other mentioned competencies, is only at an intermediate level.

**Normative competency:** According to UNESCO [72], normative competency involves "the ability to understand and reflect on the norms and values that underlie one’s actions and to negotiate sustainability values, principles, goals, and targets, in a context of conflicts of interests and different cultural contexts." It is unclear how well ChatGPT can comprehend and reflect upon norms and values that guide its actions. Given potential conflicts of interest and diverse cultural contexts, it is also uncertain whether ChatGPT can negotiate sustainability values, principles, goals, and targets. These uncertainties might be of interest to OpenAI and other stakeholders in the context of improving ChatGPT’s performance regarding SDGs.

**Strategic competency:** This is defined as "the ability to collectively design and implement interventions, transitions, and transformative governance strategies towards sustainability" [72]. While ChatGPT might be able to design and suggest interventions or strategies when given a specific problem or goal, it is uncertain whether it can design and implement such strategies collectively. This is a competency that typically relies on human intervention and collaboration. It would be interesting to consider how AI models like ChatGPT could be further developed to support strategic sustainability competency.

In summary, while ChatGPT has demonstrated a certain level of SDG literacy, its overall competency in SDG-related matters is intermediate at best. The SDG Fitness instrument’s effectiveness in assessing these competencies is somewhat limited, mainly due to the underrepresentation of some competencies. Critical thinking, self-awareness, anticipatory competency, integrated problem solving, systems thinking, normative competency, and strategic competency must be improved for ChatGPT to be considered truly SDG intelligent. These findings may provide useful insights for OpenAI and other stakeholders interested in developing AI models for sustainability solutions.

### 5.2 Mapping of SULITEST to SDG competencies

Contrary to the SDG Fitness Test [83], the SULITEST doesn’t provide detailed performance levels regarding SDG core competencies, such as intermediate or advanced. However, with a total score near 100%, it can be inferred that ChatGPT’s performance on the SULITEST about each competency might be deemed ’advanced.’ Let’s now examine the mapping of the SULITEST instrument to various SDG competencies (Table 7).

**Table 7:** Mapping of SULITEST to SDG competencies.

Competency	No. of questions mapped (X)	% of mapping (X/24 *100%)
Self-awareness	6	20%
Collaboration	4	13.3%
Critical thinking	28	93.3%
Anticipatory	26	86.7%
Integrated-problem solving	18	60%
Normative	23	76.7%
Strategic	13	43. 3%
Systems thinking	19	63. 3%

The questionnaire underrepresents three out of eight competencies (those with less than 50% mapping). These competencies include self-awareness, collaboration, and strategic competencies. Therefore, the SULITEST instrument appears to be less effective than the SDG Fitness Test at evaluating SDG intelligence. Even though a score of 117 (out of 120) might suggest that a high level of SDG literacy equates to high intelligence, the SULITEST instrument doesn’t effectively evaluate SDG intelligence concerning the competency component. This underscores the need to adjust the SULITEST instrument to ensure that underrepresented SDG competencies are represented by 50% or more. This adjustment can be accomplished in two ways:

1. Some questions could be modified to include the underrepresented SDG competencies, thereby reaching or exceeding 50% representation, without increasing the total number of questions from 30.
2. Alternatively, some questions could be modified and additional questions added to ensure that underrepresented SDG competencies are represented 50% or more, even if this means increasing the total number of questions from 30.

### 5.3 Mapping SDG fitness test to different SDGs

Determining an adequate representation of SDG types within the SDG Fitness Test questionnaire can be challenging. Since there are four sections, each containing six questions, an SDG appearing in at least six questions (or 25% of the total) could be considered a good representation. We observe that nine SDGs are mapped to 25% or more of the questions (Table 8). However, eight SDGs are underrepresented. SDGs 6, 9, and 12 are each mapped to just one question, while SDG 14 is not mapped to any questions. Hence, the SDG Fitness Test falls short of effectively assessing whether a candidate possesses SDG intelligence. This is because SDG intelligence demands a sophisticated set of competencies that span almost all the SDGs equally, implying a level of intelligence that transcends the boundaries of SDG types.

**Table 8:** Mapping of SDG fitness test to different SDGs.

SDG	No: of questions mapped (X)	% of mapping (X/24 *100%)
**SDG 17:** Partnerships for the goals	18	75.0%
**SDG 11:** Sustainable Cities & Innovation	12	50.0%
**SDG 16:** Peace, Justice & Strong Institutions	10	41.6%
**SDG 3:** Good Health & Well being	8	33.3%
**SDG 4:** Quality Education	7	29.2%
**SDG 1:** No Poverty	6	25.0%
**SDG 7:** Affordable & Clean Energy	6	25.0%
**SDG 8:** Decent work & economic growth	6	25.0%
**SDG 10:** Reduced Inequalities	6	25.0%
**SDG 5:** Gender Equality	5	20.8%
**SDG 13:** Climate Action	5	20.8%
**SDG2:** Zero Hunger	4	16.7%
SDG 6: Clean Water & Sanitation	1	4.2%
**SDG 9:** Industry Innovations & Infrastructure	1	4.2%
**SDG 12:** Responsible Consumption & Production	1	4.2%
**SDG 15:** Life on Earth	1	4.2%
**SDG 14:** Life Below Water	0	0.0%

This finding suggests revising the SDG Fitness questionnaire to ensure all the SDGs are represented by 25% or more. This revision could be approached in two ways:

1. Alter some of the existing questions in the current questionnaire to cover all the SDGs without increasing the total number of questions from 24.
2. Amend some of the current questions and add new ones to ensure the representation of all the SDGs to 25% or more, even if it increases the total number of questions from 24.

### 5.4 Mapping SULITEST to different SDGs

In the case of SULITEST, it is challenging to pinpoint an exact level of adequate representation or mapping of questions to SDG types. However, using the 25% cut-off established for the SDG Fitness Test, each SDG type should be mapped to at least eight questions to ensure proper representation. Only three SDGs ‐ SDG 11, SDG 12, and SDG 13 ‐ meet this threshold (Table 9). The remaining SDGs are underrepresented in the questionnaire, indicating that the SULITEST is ineffective in determining a candidate’s SDG intelligence regarding SDG types.

**Table 9:** Mapping of SULITEST to different SDGs.

SDG	No. of questions mapped (X)	% of mapping (X/24 *100%)
**SDG 13:** Climate Action	16	53.3%
**SDG 12:** Responsible Consumption & Production	15	50.0%
**SDG 11:** Sustainable Cities & Innovation	10	33.3%
**SDG 17:** Partnerships for the goals	7	23.3%
**SDG 9:** Industry Innovations & Infrastructure	6	20.0%
**SDG 14:** Life Below Water	6	20.0%
**SDG 15:** Life on Earth	6	20.0%
**SDG 3:** Good Health & Well being	5	16.7%
**SDG 4:** Quality Education	5	16.7%
**SDG 6:** Clean Water & Sanitation	5	16.7%
**SDG2:** Zero Hunger	4	13.3%
**SDG 10:** Reduced Inequalities	3	10.0%
**SDG 16:** Peace, Justice & Strong Institutions	3	10.0%
**SDG 7:** Affordable & Clean Energy	2	6.7%
**SDG1:** No Poverty	1	3.3%
**SDG 5:** Gender Equality	1	3.3%
**SDG 8:** Decent work & economic growth	1	3.3%

Consequently, the SULITEST instrument also requires modifications to ensure a balanced representation of the underrepresented SDG types. This could be achieved in two ways:

1. Amend some of the questions in the current questionnaire to include all the SDGs without changing the total number of questions from 30.
2. Revise some of the existing questions and introduce new ones to ensure all the SDGs are represented by 25% or more, even if this increases the total number of questions from 30.

While the SDG Fitness Test and SULITEST have been useful in addressing RQ 1 and establishing that ChatGPT can be considered SDG literate, they are not fully effective in assessing SDG intelligence. Both instruments fall short of adequately representing SDG competencies and SDG types. Of the two, the SDG Fitness Test performs better in representing SDG core competencies. Hence, ChatGPT might not be as adept in SDG intelligence considering this. These tests only give a weak indication of a lack of SDG intelligence. To better assess the SDG intelligence of ChatGPT or even human candidates, these instruments need to be refined to include the underrepresented competencies and SDG types.

This study can assist organizations in engaging their stakeholders in SDG-related discussions, thereby improving stakeholder relationships and enhancing their chances of achieving the SDGs. Furthermore, understanding the sustainability knowledge of advanced AI models like ChatGPT can help governments, international organizations, and policymakers identify the most urgent sustainability issues. This can guide them to prioritize actions promoting sustainable development, aiding content development processes, and ensuring quality.

## 6. Conclusions

Artificial Intelligence (AI), particularly large language models (LLMs) like ChatGPT, is progressively infiltrating various domains with the potential to revolutionize human life. The significant impact caused by ChatGPT, as discussed in the introduction and related literature sections, is profound. Achieving the 17 Sustainable Development Goals (SDGs) is a primary agenda integrated into many national policies. SDG literacy, a crucial criterion, aids various stakeholders in SDG-related policies or programs to attain these goals. This study attempts to evaluate the SDG literacy of ChatGPT. Experimental results using the UNESCO-provided SDG Fitness Test and SULITEST suggest that ChatGPT exhibits a high level of SDG literacy. However, it is necessary to ascertain its SDG intelligence to ensure robust reliance on ChatGPT for practical, action-oriented pursuits.

The SDG Fitness Test assesses eight cross-cutting core competencies an SDG aspirant requires at introductory, intermediate, and advanced levels. These competencies can partially evaluate SDG intelligence if adequately represented or mapped to the test questions. Mapping both questionnaires to different SDG types or covering both questionnaires concerning the 17 SDGs will assist in assessing the other part of the potential SDG intelligence of ChatGPT or a human aspirant. Upon mapping the SDG Fitness Test and SULITEST to different SDG core competencies using ChatGPT, both test questionnaires proved inadequate. While SULITEST successfully mapped only 5 out of 8 competencies to a satisfactory level, the SDG Fitness Test effectively mapped 6 out of 8 competencies. Hence, neither test is fully effective in assessing SDG intelligence, though the SDG Fitness Test performs marginally better. ChatGPT, with intermediate-level scores for six competencies, appears to exhibit limited SDG intelligence if the SDG Fitness Test is deemed fit for assessing SDG intelligence.

Regarding coverage of the SDG Fitness Test and SULITEST, i.e., mapping to 17 SDGs, both tests prove inadequate as most SDGs are underrepresented in both instruments, with some not represented at all. Therefore, both instruments are ineffective in assessing SDG intelligence via SDG coverage.

In conclusion, while ChatGPT demonstrates SDG literacy, its SDG intelligence cannot be accurately verified through existing instruments like the SDG Fitness Test and SULITEST. However, as the SDG Fitness Test has shown better performance than SULITEST in mapping SDG competencies if deemed adequate for assessing SDG intelligence via core competencies, ChatGPT may not be considered SDG intelligent. Both instruments need modifications for proper validation to ensure adequate representation of core competencies and SDG types.

As is examined above, the SDG Fitness Test’s mapping to core competencies weakly indicates that ChatGPT lacks SDG intelligence; future versions of ChatGPT could be developed to enhance several competencies such as collaboration, critical thinking, systems thinking, anticipatory, integrated problem-solving, and self-awareness. This is a recommendation to OpenAI if they aim to contribute to achieving the SDGs. This study also suggests refining existing theoretical frameworks regarding AI’s role in sustainable development and identifying new research questions and directions. For instance, researchers could extend studies to examine the effect of different training approaches on AI models’ sustainability awareness or the potential trade-offs between sustainability performance and other performance metrics.

National and international policymakers striving towards achieving the SDGs should limit the use of ChatGPT to information gathering and possibly action suggestions rather than decision-making, particularly related to action plans. This is due to the current limitations of ChatGPT (GPT-4), such as the potential for misinformation or "hallucinations" [25]. There are ongoing ethical considerations regarding the application of ChatGPT across diverse fields such as education, medical writing and diagnosis, and legal practice.

Before ChatGPT’s deployment in SDG-related tasks and interventions can be seriously contemplated, two conditions must be met: ethical clearance from experts in various research fields or sectors and the attainment of the aforementioned core competencies by ChatGPT. Once these conditions are satisfied, ChatGPT could be a potent tool in various policy-related tasks and interventions concerning the SDGs. However, until then, its usage should be considered with care, emphasizing its role as an aid for gathering information and suggesting actions rather than as a decision-making tool.

In conclusion, AI models like ChatGPT hold great potential for contributing to sustainable development and achieving the SDGs. Yet, we must tread carefully, considering their current limitations and ethical implications, while continuing to refine and enhance these models for better representation of SDGs and improved competency.

## 7. How SDG literacy of ChatGPT can be leveraged

As previously noted, while ChatGPT is SDG-literate and can assist with information provision and possibly offer insights, it should not be entrusted with decision-making responsibilities. Organizations can harness ChatGPT’s understanding to improve their sustainability strategies, pinpointing areas of their operations that significantly influence the SDGs. This could aid in the implementation of efficient sustainability practices. For instance, ChatGPT could offer guidance on designing environmentally conscious products and services or promoting social equality in recruitment and promotional practices. Other stakeholders can utilize ChatGPT according to their specific needs. For instance, UN bodies like UNESCO could leverage ChatGPT to create content for various short and long-term training programs, thus cultivating a workforce dedicated to sustainability.

In light of the recent COVID-19 pandemic, the potential of AI LLM bots could be harnessed to plan for and mitigate future pandemics, contributing significantly to SDG 3. Similar applications could be explored for disaster prevention planning and mitigation, impacting various SDGs and their targets.

## 8. Limitations of this study and possible future explorations

This study is not without its limitations. It became apparent that ChatGPT requires a comprehensive understanding of the SDGs, including the goals, targets, and indicators, as well as the socio-economic, cultural, and political factors that influence their implementation. This could lead to biases, inaccuracies, or incomplete responses when generating SDG knowledge. However, mapping SDG competencies and SDG types was conducted by experimenting with ChatGPT. Validating the accuracy of these mappings would require gathering and analyzing responses from human experts, a task beyond the scope of this study but worthy of future investigation. Further research could also explore the knowledge or intelligence of ChatGPT and similar AI models regarding individual SDGs, necessitating the identification or design of suitable instruments. This presents another potential avenue for future research.

As already mentioned, the examined instruments (SDG Fitness Test and SULITEST) are found to be inadequate to assess the SDG intelligence of ChatGPT or human candidates due to the underrepresentation of cross-cutting competencies and SDG types. Diligent modification of these instruments by modifying the instrument without the addition of more questions is a challenging endeavour that can be attempted in collaboration with the administrators of both instruments. Research directed on strengthening the instrument by the addition of more but limited number of questions, possibly by incorporating the missing elements in the instrument that might be present in other available instruments (some of them are mentioned in the introduction), is also worthy of pursuit. In both cases, each cross-cutting competency and each SDG type should be adequately represented, and we suggested 25% coverage as a tentative benchmark that can be targeted by the developers/administrators of both instruments, viz. UNITAR and SULI test administrators. LLM developers, including OpenAI, can target to improve the cross-cutting competencies and knowledge related to all SDG types by (i) searching for more comprehensive test instruments than these instruments or (ii) by developing more comprehensive instruments either in-house or in consultation with UNITAR, SULI test administrators or any other, and provide rigorous training to LLMs to gain these competencies and improve knowledge related to different SDGs by expanding their training corpus to cover more SDG related materials. Another possible way to improve the SDG intelligence of ChatGPT or LLMs is by training those with as many case studies (of success as well as failure) as possible related to each SDG from different parts of the world. Case studies related to each cross-cutting competency from the SDG background and the general managerial stream can also be used for training purposes. Apart from assessing SDG intelligence, the general leadership/managerial ability of ChatGPT and AI LLM bots can be evaluated using standard psychometric instruments. The development of LLMs for managerial applications exclusively like its counterpart in medicine and healthcare (like Med-PaLM by Google) is also a great avenue of opportunity. Another possible research opportunity lies in the development of LLMs exclusively for SDG applications.

## Supporting information

S1 File SDG literacy dataset. This has details about the SDG literacy test.
(DOCX)
