# Supplementary material for: ChatGPT: Literate or intelligent about UN sustainable development goals?
Source: PLoS One. 2024 Apr 24;19(4):e0297521. doi: 10.1371/journal.pone.0297521 (PMC11042716; doi:10.1371/journal.pone.0297521)
Supplement: S1 File — This has details about the SDG literacy test. (DOCX) [file pone.0297521.s001.docx]

**Dataset – SDGLearn**

Question 1 While the SDGs were conceived with national governments in mind, cities around the world have begun to pursue direct implementation of the SDGs. A key part of this process is committing formally via Voluntary Local Review (VLR). VLRs typically include aligning local strategy to the SDGs, facilitating local stakeholder engagement in tackling the SDGs, and submitting progress reports to the United Nations. The mayor of your city (population 250,000) announced last week that they will be carrying out just such a VLR and you have been brought on to lead the initiative. Question 1 While the announcement from the mayor is fresh on everyone’s mind you quickly organize the first VLR meeting with representatives from an array of city departments. Your hopes for quick progress are dashed when the meeting is derailed by arguing, side discussions, and other issues. Despite the tight deadline you realize that the next meeting has to be focused on effective teamwork in sustainability. Which of the following would you NOT include as practices your team will follow on this project? Choose the best 2 answers. Make commitments through the establishment of a shared vision with clear goals and be sure to follow through with them. Even in this digital era, making time for face-to-face contact within the team is critical for effective teamwork in sustainability. Always prioritize the completion of assigned tasks over resolving interpersonal issues during official team meetings. Appreciate and reward individual team members or sub-teams in addition to focusing on the whole team's progress. Actively seek out and implement tools whose use can help foster the collaborative teamwork desired and seek out additional training if necessary. Let the roles of each team member and team logistics emerge organically over time.

The best 2 answers to NOT include as practices your team will follow on this project are:

1. Always prioritize the completion of assigned tasks over resolving interpersonal issues during official team meetings.
2. Let the roles of each team member and team logistics emerge organically over time.

To which all SDGs and specific targets within those SDGs, the above question possibly aligns with? Please justify your selection of SDGs

The question above aligns with the following SDGs and specific targets:

1. SDG 11: Sustainable Cities and Communities
   - Target 11.3: By 2030, enhance inclusive and sustainable urbanization and capacity for participatory, integrated, and sustainable human settlement planning and management in all countries. The question discusses the implementation of the SDGs at the city level, which directly relates to promoting sustainable urban development.
2. SDG 16: Peace, Justice, and Strong Institutions
   - Target 16.7: Ensure responsive, inclusive, participatory, and representative decision-making at all levels. The question highlights the importance of effective teamwork, collaboration, and inclusiveness in local government for the successful implementation of the SDGs, which contributes to building strong institutions.
3. SDG 17: Partnerships for the Goals
   - Target 17.17: Encourage and promote effective public, public-private, and civil society partnerships, building on the experience and resourcing strategies of partnerships. The question implies the involvement of various city departments and stakeholders in the Voluntary Local Review process, which supports partnership development for achieving the SDGs.

These SDGs and their specific targets are relevant because they emphasize the importance of inclusive, participatory, and collaborative approaches to sustainable development at the local level, and the need for effective teamwork and partnerships to achieve the global goals.

Question 2 For the first VLR, the decision was made to give a particular focus to SDG 13: Climate Action. Therefore, the environmental department is leading a comprehensive analysis of the current state of the city in terms of climate change. The objective of this analysis is to understand the most important ways in which the city contributes to climate change. This will include investigating what the structures, policies, values, and so on that influence the situation right now. How would you guide the team’s efforts? Choose the best 2 answers. Only include elements that can be measured and formally modeled (e.g., energy use). Hold-off until a later point on specifying solutions and strategies for achieving SDG 13. Avoid discussing anything related to other SDGs. Consult with other city departments such as housing and economic development. Focus only on how local climate policies affect the city and not national or regional levels. Identify and quantify every possible source of greenhouse gas emissions connected to the city.

The best 2 answers for guiding the team's efforts are:

1. Hold-off until a later point on specifying solutions and strategies for achieving SDG 13.
2. Consult with other city departments such as housing and economic development.

To which all SDGs and specific targets within those SDGs, the above question possibly aligns with? Please justify your selection of SDGs

The question above aligns with the following SDGs and specific targets:

1. SDG 13: Climate Action
   - Target 13.2: Integrate climate change measures into national policies, strategies, and planning. The question focuses on analyzing the current state of the city in terms of climate change and its contributions, which is a crucial step in integrating climate action into local policies and strategies.
   - Target 13.3: Improve education, awareness-raising, and human and institutional capacity on climate change mitigation, adaptation, impact reduction, and early warning. The comprehensive analysis mentioned in the question can be a means to increase awareness and capacity building among city departments and local stakeholders.
2. SDG 17: Partnerships for the Goals
   - Target 17.14: Enhance policy coherence for sustainable development. By consulting with other city departments such as housing and economic development, the team can ensure policy coherence and create synergies between climate action and other aspects of sustainable development.
   - Target 17.16: Enhance the global partnership for sustainable development, complemented by multi-stakeholder partnerships that mobilize and share knowledge, expertise, technology, and financial resources. Collaborating with various city departments and stakeholders during the analysis will encourage partnerships and sharing of expertise to address climate change.

The selected SDGs and their specific targets are relevant because they emphasize the need for a comprehensive understanding of the city's climate contributions, integrating climate action into local policies, and fostering partnerships across various sectors for sustainable development.

Question 3 Q3 - A key part of the report that you are developing will include a VISION of what the city will look like in 2030 after making significant progress towards the SDGs. Which of the following are correct statements about the sustainability vision you will develop? Choose the best 2 answers. It should focus on sustainability solutions. It should be able to be enacted in whole immediately. It should be extrapolated directly from current trends. It should include values such as human rights. It should be inspirational and does not have to be grounded in reality. It should focus on current problems and the tactics for solving them.

The best 2 answers for correct statements about the sustainability vision you will develop are:

1. It should focus on sustainability solutions.
2. It should include values such as human rights.

To which all SDGs and specific targets within those SDGs, the above question possibly aligns with? Please justify your selection of SDGs

The question above aligns with the following SDGs and specific targets:

1. SDG 11: Sustainable Cities and Communities
   - Target 11.3: By 2030, enhance inclusive and sustainable urbanization and capacity for participatory, integrated, and sustainable human settlement planning and management in all countries. Creating a sustainability vision for the city that focuses on solutions and incorporates values such as human rights aligns with this target, as it involves envisioning an inclusive and sustainable urban future.
2. SDG 16: Peace, Justice, and Strong Institutions
   - Target 16.7: Ensure responsive, inclusive, participatory, and representative decision-making at all levels. Developing a sustainability vision that includes human rights and focuses on solutions contributes to promoting responsive and inclusive decision-making processes for a more sustainable and equitable city.
3. SDG 17: Partnerships for the Goals
   - Target 17.14: Enhance policy coherence for sustainable development. A sustainability vision that integrates various aspects of sustainability and human rights can guide the city in creating coherent policies that address multiple dimensions of sustainable development.

The selected SDGs and their specific targets are relevant because they emphasize the importance of developing a comprehensive, inclusive, and solution-focused vision for sustainable urban development that incorporates human rights and promotes policy coherence.

Question 4 Q4- After several months of work everything seems to be falling apart around you. Several departments have stopped sending representatives to meetings, commercial interests are complaining loudly about how this whole thing is anti-growth, and a stakeholder engagement session was only attended by local homeowners furious that low-income housing might be proposed in their neighborhoods, among other issues. At the same time the mayor’s office is breathing down your neck to get this done yesterday. A colleague of yours suggests that you should give up the stakeholder input and visioning you have been working on; instead, you should work directly on the policy recommendation to the mayor. Imagine yourself in this situation. Which 2 statements best describe your EMOTIONS? Frustrated because you could do a better VLR if you didn’t have to engage so many different groups. Angry at all the people who seem to just want to stand in the way of progress. Empathetic to the concerns of the homeowners and interested in getting past their anger. Resigned and accepting of your colleague’s suggestion to just move on to the policy recommendation. Motivated to continue with the problem-solving process despite the challenges. Discouraged about the whole enterprise and ready to give up.

As an AI language model, I don't have emotions. However, I can suggest 2 statements that may describe the emotions someone in this situation could potentially feel:

1. Frustrated because you could do a better VLR if you didn’t have to engage so many different groups.
2. Motivated to continue with the problem-solving process despite the challenges.

To which all SDGs and specific targets within those SDGs, the above question possibly aligns with? Please justify your selection of SDGs

The question above aligns with the following SDGs and specific targets:

1. SDG 11: Sustainable Cities and Communities
   - Target 11.3: By 2030, enhance inclusive and sustainable urbanization and capacity for participatory, integrated, and sustainable human settlement planning and management in all countries. The situation described in the question involves challenges in stakeholder engagement and collaboration among various city departments, which are crucial aspects of achieving inclusive and sustainable urban development.
2. SDG 16: Peace, Justice, and Strong Institutions
   - Target 16.6: Develop effective, accountable, and transparent institutions at all levels. Addressing conflicts and disagreements among stakeholders and city departments, as well as ensuring transparency and accountability in the process, are important for building strong institutions and promoting sustainable development.
3. SDG 17: Partnerships for the Goals
   - Target 17.16: Enhance the global partnership for sustainable development, complemented by multi-stakeholder partnerships that mobilize and share knowledge, expertise, technology, and financial resources. The question highlights the challenges in building effective partnerships and engaging stakeholders in the process of working toward the SDGs.

The selected SDGs and their specific targets are relevant because they emphasize the importance of collaboration, stakeholder engagement, and building strong institutions to achieve sustainable urban development and promote effective partnerships for the Goals.

Question 5 One day late in the evening you get a call from the mayor, who assures you that she personally believes in the process that you have set-up and will provide additional funding and take some heat if that’s what is needed to do the Voluntary Local Review correctly. With fresh wind in your sails, you focus on developing strategies for one of the biggest impact areas of your cities—the majority of residents who drive their private vehicles. Your team, with the input of an array of stakeholders, has developed a series of solutions which would make significant progress towards the sustainability vision articulated in the report. Besides infrastructure (e.g., bike lanes, more bus routes, etc.) what else needs to be part of your sustainability solution strategy? Choose the best 2 answers. Identify potential barriers to successful implementation and how they might be overcome. Describing the infrastructure solutions in detail is sufficient for a sustainability solution strategy. An extensive systematic analysis of the ways in which private vehicle travel exacerbates inequality and greenhouse gas emissions in general. Try to be exhaustively comprehensive with your strategy that it never needs to be revisited or modified regardless of obstacles implementation may face. Avoid focusing on power or politics which might hamper or support implementation to not “awaken sleeping dogs”. Articulate a theory of change to explain how the solutions proposed lead to achieving the SDGs.

The best 2 answers for what else needs to be part of your sustainability solution strategy are:

1. Identify potential barriers to successful implementation and how they might be overcome.
2. Articulate a theory of change to explain how the solutions proposed lead to achieving the SDGs.

To which all SDGs and specific targets within those SDGs, the above question possibly aligns with? Please justify your selection of SDGs

The question above aligns with the following SDGs and specific targets:

1. SDG 11: Sustainable Cities and Communities
   - Target 11.2: By 2030, provide access to safe, affordable, accessible, and sustainable transport systems for all, improving road safety, notably by expanding public transport, with special attention to the needs of those in vulnerable situations, women, children, persons with disabilities, and older persons. The question's focus on developing strategies to reduce private vehicle usage and promote sustainable transportation alternatives directly aligns with this target.
2. SDG 13: Climate Action
   - Target 13.2: Integrate climate change measures into national policies, strategies, and planning. By addressing private vehicle usage, which is a significant source of greenhouse gas emissions, the proposed sustainability solution strategy contributes to integrating climate change measures into the city's policies and strategies.
3. SDG 17: Partnerships for the Goals
   - Target 17.14: Enhance policy coherence for sustainable development. Identifying potential barriers to implementation and articulating a theory of change for the proposed solutions can help ensure that the city's policies are coherent and aligned with the SDGs.

The selected SDGs and their specific targets are relevant because they emphasize the importance of sustainable transportation, integrating climate action into local policies, and ensuring policy coherence in achieving sustainable urban development and the global goals.

Question 6 You get home after a long drive and collapse into bed. The press conference announcing the release of your VLR was a resounding success with positive feedback pouring in from community groups, media, local politicians, and many others. You are ready to forget about it all, but something keeps nagging at the back of your mind. The city’s largest impact on the climate is emissions from private cars, one of which you drive up and back for well over an hour each day. Imagine yourself in this situation, which actions would you be most likely to take? Choose the best 2 answers. Look up the public transit options from your house to work but if they are more time consuming than your current commute not pursue it further Reflect on your feelings for several days, taking the opportunity to critically evaluate what has led you to personally have such big impact due to commuting. Not worry too much about it, your job is to help the city be more sustainable not change your own practices. That there is nothing you can do because as a parent raising a family you have to have a big enough house and you can only afford it in the suburbs distant from your work. Cancel meetings the next day and take time to apply the same calculations of impact from transportation to your own lifestyle that you just did to the whole city. Discuss your feelings with a colleague the next day and realize that your role as a change agent is through your job and your personal decisions are irrelevant.

The best 2 answers for the actions you would be most likely to take in this situation are:

1. Look up the public transit options from your house to work but if they are more time-consuming than your current commute not pursue it further.
2. Reflect on your feelings for several days, taking the opportunity to critically evaluate what has led you to personally have such a big impact due to commuting.

To which all SDGs and specific targets within those SDGs, the above question possibly aligns with? Please justify your selection of SDGs

The question above aligns with the following SDGs and specific targets:

1. SDG 11: Sustainable Cities and Communities
   - Target 11.2: By 2030, provide access to safe, affordable, accessible, and sustainable transport systems for all, improving road safety, notably by expanding public transport, with special attention to the needs of those in vulnerable situations, women, children, persons with disabilities, and older persons. The question highlights the personal aspect of sustainable transportation choices and how individual actions can contribute to sustainable urban development.
2. SDG 12: Responsible Consumption and Production
   - Target 12.8: By 2030, ensure that people everywhere have the relevant information and awareness for sustainable development and lifestyles in harmony with nature. Reflecting on personal commuting choices and considering more sustainable alternatives aligns with this target, as it encourages individuals to become more aware of their consumption patterns and make informed decisions to reduce their environmental impact.
3. SDG 13: Climate Action
   - Target 13.3: Improve education, awareness-raising, and human and institutional capacity on climate change mitigation, adaptation, impact reduction, and early warning. By considering the personal impact of commuting choices on climate change, the question touches upon the importance of awareness-raising and personal responsibility in addressing climate change.

The selected SDGs and their specific targets are relevant because they emphasize the importance of sustainable transportation choices, responsible consumption patterns, and raising awareness about individual actions that contribute to climate change and sustainable development.

You are working for an NGO which has won a grant to help improve animal husbandry in a struggling rural region. The winning proposal was based on the concept of Community-Based Animal Health Workers (CAHW) a program deployed successfully in a neighbouring country. This program trained local community members in basic veterinary tasks, improved husbandry practices, and provided necessary supplies and equipment. You have been tasked with implementing this project.

Question 7 After the initial excitement has worn off you are now feeling a lot of self-doubt. You are new to the targeted region and have only limited experience with animal husbandry in general. Then you remember that in your past work and studies, you have successfully designed and implemented other similarly structured projects such as one that trained community health workers to administer basic care. What are some initial actions which you would plan to take in light of your experiences with problem-solving that will help you gain initial perspective? Choose the best 2 answers. Begin implementation immediately, planning to learn by doing, and adjusting the project on the fly. Banish the self-doubt you are feeling reminding yourself that such reflections get in the way of the confidence you will need to manage this complex project. Identify key stakeholders and complementary experts who you set-up meetings and interviews with and who you hope to integrate into the entire planning process. It being your job, you take personal responsibility and get to work in the office carefully designing an implementation plan based only on your relevant expertise. For your planning process, you work with veterinarians in the capital city to first develop a strategic implementation plan. Reflect on your own strengths and explicitly identify deficits where you will need significant support (e.g., local expertise).

Identify key stakeholders and complementary experts who you set-up meetings and interviews with and who you hope to integrate into the entire planning process.

Reflect on your own strengths and explicitly identify deficits where you will need significant support (e.g., local expertise).

To which all SDGs and specific targets within those SDGs, the above question possibly aligns with? Please justify your selection of SDGs

The question described above aligns with the following Sustainable Development Goals (SDGs) and specific targets:

1. SDG 1: No Poverty
   - Target 1.4: Ensuring that all men and women, particularly the poor and vulnerable, have equal rights to economic resources and access to basic services. The project aims to improve animal husbandry practices in a struggling rural region, which can lead to increased income and better living conditions for the local community.
2. SDG 2: Zero Hunger
   - Target 2.3: Doubling the agricultural productivity and incomes of small-scale food producers, particularly through improved livestock management. The Community-Based Animal Health Workers program is designed to enhance animal husbandry, leading to better productivity and income generation for rural farmers.
3. SDG 3: Good Health and Well-being
   - Target 3.8: Achieving universal health coverage, including financial risk protection and access to quality essential healthcare services. By training community members in basic veterinary tasks and improving animal health, the project contributes to better public health in the region, as healthy livestock are crucial for food security and nutrition.
4. SDG 4: Quality Education
   - Target 4.4: Increasing the number of youth and adults who have relevant skills for employment, decent jobs, and entrepreneurship. The CAHW program trains community members in veterinary and animal husbandry practices, equipping them with valuable skills for employment and entrepreneurship in the agricultural sector.
5. SDG 8: Decent Work and Economic Growth
   - Target 8.3: Promoting development-oriented policies that support productive activities, decent job creation, and entrepreneurship. The project promotes decent work and sustainable economic growth by enhancing local capacity for animal husbandry and veterinary care, potentially creating new job opportunities and improving overall community well-being.

These selected SDGs are justified because the question emphasizes community development, poverty alleviation, and skill-building in the context of animal husbandry and veterinary care. By implementing the project, the NGO contributes to achieving these SDGs and their specific targets, ultimately promoting sustainable development in the targeted region.

Question 8 One of your first tasks will be determining which animal husbandry tasks should be promoted by the project. You discover through your research and consultation with experts that there has recently been a big outbreak of anthrax, and that no one is sure whether it was a one-off issue or the beginning of an ongoing problem for the region. Given this, how could you make informed decisions about selecting husbandry tasks to be promoted? Choose the best 2 answers. Run a collaborative brainstorming session to create an inspiring vision as your possible future scenario for analysis. Develop at least two scenarios of the future if anthrax returns on a regular basis and if it doesn’t. Pick a single trend (e.g., changing future temperatures) with which to evaluate how the future of anthrax outbreaks might play out. Analyze different scenarios to see which tasks would be best for CAHWs to promote for each. Rely primarily on the quantitative epidemiological models to forecast exactly what level of anthrax infections are to be expected. Study what has worked best in the past as the future is too uncertain to be of any use for making decisions.

Develop at least two scenarios of the future if anthrax returns on a regular basis and if it doesn’t.

Analyze different scenarios to see which tasks would be best for CAHWs to promote for each.

To which all SDGs and specific targets within those SDGs, the above question possibly aligns with? Please justify your selection of SDGs

The question described above aligns with the following Sustainable Development Goals (SDGs) and specific targets:

1. SDG 2: Zero Hunger
   - Target 2.3: Doubling the agricultural productivity and incomes of small-scale food producers, particularly through improved livestock management. By selecting appropriate husbandry tasks to tackle issues like anthrax outbreaks, the project aims to improve agricultural productivity and income for rural farmers.
2. SDG 3: Good Health and Well-being
   - Target 3.3: Ending the epidemics of communicable diseases, such as anthrax, by addressing potential outbreaks through proper animal husbandry practices. By focusing on anthrax prevention, the project will contribute to better public health in the region.
   - Target 3.d: Strengthening the capacity of countries for early warning, risk reduction, and management of national and global health risks. Developing scenarios for possible anthrax outbreaks and preparing accordingly helps build resilience against potential health risks.
3. SDG 9: Industry, Innovation, and Infrastructure
   - Target 9.5: Enhancing scientific research, upgrading technological capabilities of industrial sectors, and encouraging innovation. By analyzing different scenarios and incorporating innovative husbandry practices, the project contributes to the development of more effective strategies for managing livestock health and addressing potential disease outbreaks.
4. SDG 15: Life on Land
   - Target 15.3: Combating desertification, restoring degraded land and soil, and promoting sustainable land management. By improving animal husbandry practices, the project can contribute to sustainable land management and the overall health of ecosystems in the region.

These selected SDGs are justified because the question focuses on informed decision-making in the context of potential anthrax outbreaks and their impact on animal husbandry practices. The project's efforts to analyze different scenarios and select the best tasks for Community-Based Animal Health Workers to promote align with these SDGs and their specific targets, ultimately contributing to sustainable development in the targeted region.

Question 9 In the funded proposal the project was described as “a cost-efficient model to improve the quality of animal health services and contribute to improved livelihood assets in rural populations.” You are now facing the task to operationalize these principles of cost-efficiency, quality of animal health services, and livelihood assets into concrete targets and indicators. Which of the following are important to the development of indicators for this project? Choose the best 2 answers. Include indicators focused on livestock health exclusively as that is the project’s purpose. Distinguish between success (e.g., improvement in access for women to veterinary services) and targets (e.g., gender parity in access) for each indicator. Organize focus groups or other approaches to gather input from diverse stakeholders regarding the development of indicators. Solicit only the input of experts with sufficient technical knowledge to dictate the development of the indicators. Develop indicators that can be easily measured on a cost benefit analysis with the threshold for success being “net benefits exceeding net costs”. Eliminate any indicators around human rights as these are “values” based and indicators should be value-free and “scientific”.

Distinguish between success (e.g., improvement in access for women to veterinary services) and targets (e.g., gender parity in access) for each indicator.

Organize focus groups or other approaches to gather input from diverse stakeholders regarding the development of indicators.

To which all SDGs and specific targets within those SDGs, the above question possibly aligns with? Please justify your selection of SDGs

The question described above aligns with the following Sustainable Development Goals (SDGs) and specific targets:

1. SDG 1: No Poverty
   - Target 1.4: Ensuring that all men and women, particularly the poor and vulnerable, have equal rights to economic resources and access to basic services. By developing indicators that measure improvements in rural populations' livelihood assets, the project aims to address poverty and promote economic empowerment.
2. SDG 2: Zero Hunger
   - Target 2.3: Doubling the agricultural productivity and incomes of small-scale food producers, particularly through improved livestock management. Establishing indicators to measure the quality of animal health services and their impact on agricultural productivity can contribute to achieving this target.
3. SDG 5: Gender Equality
   - Target 5.a: Undertaking reforms to give women equal rights to economic resources, access to ownership, and control over land and other forms of property, financial services, and natural resources. By considering gender parity in access to veterinary services as a target, the project addresses gender equality in the context of animal husbandry and rural livelihoods.
4. SDG 8: Decent Work and Economic Growth
   - Target 8.3: Promoting development-oriented policies that support productive activities, decent job creation, and entrepreneurship. By focusing on improving livelihood assets and the quality of animal health services, the project contributes to decent work and sustainable economic growth in the targeted region.
5. SDG 17: Partnerships for the Goals
   - Target 17.18: Enhancing the availability of high-quality, timely, and reliable data disaggregated by income, gender, age, and other characteristics relevant to the project. Developing concrete targets and indicators for cost-efficiency, quality of animal health services, and livelihood assets can contribute to better data collection and monitoring of the project's impact.

These selected SDGs are justified because the question emphasizes the need to operationalize the principles of cost-efficiency, quality of animal health services, and livelihood assets into concrete targets and indicators. By doing so, the project contributes to achieving these SDGs and their specific targets, ultimately promoting sustainable development in the targeted region.

Question 10 After weeks of work and coordination, you have set up a full-day workshop with a diverse set of stakeholders—local politicians, livestock farmers, religious leaders, and more. While not completely balanced, there was a reasonable proportion of women participants as well. The discussions, activities, and interactive sessions were going well. You are collecting lots of very valuable information and perspectives that you would not have been able to obtain otherwise, such as challenges to consistently delivering supplies in certain districts. During the lunch break, you and your team take a chance to review the workshop and make any adjustments. One of your female colleagues points out that not a single of the woman participants has spoken in any of the public sessions. Another colleague responds that we did our job getting them there and we can’t force the women to speak up. Imagine yourself in this situation. Which 2 statements best describe your EMOTIONS? Glad there is a woman on your team as that is their job to catch such omissions. Disappointed not to have heard from the women participants but satisfied that the workshop has enabled their physical presence. Disconcerted that among the whole team (including yourself) only one member noticed the issue and feeling a need to reflect on the implications of this. Unconcerned about who specifically has not spoken up at the workshop as they probably just didn’t have anything they felt was important enough to say. In strong agreement with your colleague that you need to change things up so as to hear the voices of the women participants. Frustrated with the women participants for not speaking up and putting the team in this bind.

Disconcerted that among the whole team (including yourself) only one member noticed the issue and feeling a need to reflect on the implications of this.

In strong agreement with your colleague that you need to change things up so as to hear the voices of the women participants.

To which all SDGs and specific targets within those SDGs, the above question possibly aligns with? Please justify your selection of SDGs

The question described above aligns with the following Sustainable Development Goals (SDGs) and specific targets:

1. SDG 5: Gender Equality
   - Target 5.1: Ending all forms of discrimination against all women and girls everywhere. By recognizing the lack of women's voices in the workshop and aiming to address it, the project is working towards promoting gender equality and ensuring that women's perspectives are considered in decision-making.
   - Target 5.5: Ensuring women's full and effective participation and equal opportunities for leadership at all levels of decision-making in political, economic, and public life. By adjusting the workshop to encourage women's participation, the project supports the inclusion of women in the discussions and decision-making processes related to animal husbandry and rural development.
2. SDG 10: Reduced Inequalities
   - Target 10.2: Empowering and promoting the social, economic, and political inclusion of all, irrespective of age, sex, disability, race, ethnicity, origin, religion, or economic or other status. By attempting to include women's voices in the workshop, the project is working towards reducing inequalities and ensuring that diverse perspectives are considered.
3. SDG 16: Peace, Justice, and Strong Institutions
   - Target 16.7: Ensuring responsive, inclusive, participatory, and representative decision-making at all levels. By identifying the need to include women's voices in the workshop and taking action to encourage their participation, the project is contributing to more inclusive and representative decision-making processes related to the project's objectives.
4. SDG 17: Partnerships for the Goals
   - Target 17.9: Enhancing international support for implementing effective and targeted capacity-building in developing countries to support national plans to implement all the SDGs. By organizing a diverse workshop with various stakeholders and striving to include women's perspectives, the project is building capacity and fostering partnerships that can contribute to the achievement of the SDGs.

These selected SDGs are justified because the question emphasizes the need to address gender equality and ensure women's participation in the workshop. By recognizing and attempting to rectify the lack of women's voices in the discussions, the project contributes to achieving these SDGs and their specific targets, ultimately promoting sustainable development in the targeted region.

Question 11 You and your team have developed three alternative implementation strategies based on the integrated problem-solving process you have gone through. You will need to decide which strategy the project will take in establishing a network of CAHWs. In choosing among these strategies which of the following actions would you take to assure eventual success? Choose the best 2 answers. Solicit the support of local organizations such as the national veterinary services for one or more of the strategies. Await the results of a third academic study on the original CAHW project to be absolutely sure of the science before moving forward with implementing any strategy. Assess which strategy is likely to have the greatest impact on poverty as this is the only metric in which a quantitative analysis can be conducted. Analyze how each of the strategies would play out if applied to your original understanding of the current, problematic situation. Determine which strategy would improve animal health the fastest even though that would mean less training on other improved husbandry practices.

Solicit the support of local organizations such as the national veterinary services for one or more of the strategies.

Analyze how each of the strategies would play out if applied to your original understanding of the current, problematic situation.

To which all SDGs and specific targets within those SDGs, the above question possibly aligns with? Please justify your selection of SDGs

The question described above aligns with the following Sustainable Development Goals (SDGs) and specific targets:

1. SDG 1: No Poverty
   - Target 1.4: Ensuring that all men and women, particularly the poor and vulnerable, have equal rights to economic resources and access to basic services. By selecting an implementation strategy that addresses the challenges faced by the rural population and supports their livelihoods, the project aims to reduce poverty and enhance economic well-being.
2. SDG 2: Zero Hunger
   - Target 2.3: Doubling the agricultural productivity and incomes of small-scale food producers, particularly through improved livestock management. Choosing a strategy that improves animal health and husbandry practices will contribute to increased agricultural productivity and food security.
3. SDG 3: Good Health and Well-being
   - Target 3.3: Ending the epidemics of communicable diseases, such as those associated with poor animal husbandry. By selecting a strategy that improves animal health, the project can contribute to better public health in the region.
4. SDG 8: Decent Work and Economic Growth
   - Target 8.3: Promoting development-oriented policies that support productive activities, decent job creation, and entrepreneurship. By choosing a strategy that focuses on establishing a network of CAHWs, the project contributes to job creation and sustainable economic growth in the targeted region.
5. SDG 17: Partnerships for the Goals
   - Target 17.16: Enhancing the global partnership for sustainable development, complemented by multi-stakeholder partnerships that mobilize and share knowledge, expertise, technology, and financial resources. By soliciting the support of local organizations, the project fosters collaboration and strengthens partnerships to achieve the SDGs.

These selected SDGs are justified because the question focuses on choosing an implementation strategy for establishing a network of Community-Based Animal Health Workers. The actions taken to assure the eventual success of the project contribute to these SDGs and their specific targets, ultimately promoting sustainable development in the targeted region.

Question 12 It has been several months since your implementation plan was rolled out and things seem to be going very well. Your local implementing partners have sent back enthusiastic reports of success. On several phone calls, they have told you that they have trained more CAHWs than was anticipated and that local reception for them has been very positive. This is great news because you have a video conference with the funders early the next morning (due to differing time zones). Just then your email pings with the quarterly monitoring reports. Which of the following would you do? Choose the best 2 answers. Conduct a quick, descriptive analysis of the monitoring report data even though it gets in the way of working on your presentation. Continue to develop the presentation for the funders as the priority is to get it highly polished. Reflect on the reliability of the phone-based conversations as the exclusive basis for analysis. Base your opinion about the success of the project on the verbal reports you have as the callers are in the field and you are not. Rely on what you heard over the phone even if the monitoring reports conflict with what you were told. Work to convey your excitement about the positive reports from your implementing partners into the presentation.

Conduct a quick, descriptive analysis of the monitoring report data even though it gets in the way of working on your presentation.

Reflect on the reliability of the phone-based conversations as the exclusive basis for analysis.

To which all SDGs and specific targets within those SDGs, the above question possibly aligns with? Please justify your selection of SDGs

The question described above aligns with the following Sustainable Development Goals (SDGs) and specific targets:

1. SDG 9: Industry, Innovation, and Infrastructure
   - Target 9.5: Enhancing scientific research, upgrading the technological capabilities of industrial sectors in all countries. By conducting an analysis of the monitoring report data, the project can contribute to evidence-based decision-making and continuous improvement, which aligns with the goal of promoting innovation.
2. SDG 17: Partnerships for the Goals
   - Target 17.18: Enhancing capacity-building support to developing countries, including for data collection and monitoring of the SDGs. By analyzing the monitoring report data and reflecting on the reliability of information sources, the project ensures that data-driven decision-making is used to assess progress and adapt as needed. This aligns with the goal of strengthening partnerships and fostering sustainable development.

These selected SDGs are justified because the question focuses on the importance of evidence-based decision-making, especially when evaluating the success of a project. By conducting a quick analysis of the monitoring report data and reflecting on the reliability of phone-based conversations, the project's actions contribute to these SDGs and their specific targets, promoting sustainable development in the targeted region.

Scenario 3 Several years ago, you founded a solar company that provides solar systems to businesses and households in the capital city. Recently you read about an award-winning Enhanced Rural Resilience (ERR) project that was implemented abroad and whose primary objective was to establish decentralized solar energy systems. But the program did more than just seek to make solar energy accessible and affordable, it also focused on supporting women, improving employability skills, and more as part of an integrated program. You feel that your company could spark such an initiative in your own country. Question 13 The best opportunity to fund this project is a recently launched funding mechanism for a public-private partnership that seeks to stimulate rural economic development. Your proposed ERR-inspired project is a finalist, and you are invited to give a presentation to the award panel. During your presentation, you are getting skeptical looks and finally one of the panelists says, “I can see how micro-solar power is a good thing, but I don’t see how it would stimulate economic development.” You respond by explaining the feedback loop that connects micro-solar and economic development using which of the following statements? Choose the best 2 answers. Consistency of solar electricity price as compared to the current diesel generated means that households and businesses can make long term financial plans, including the purchase of financed micro-solar which increases the ratio of electricity coming from stably priced solar in the future. The micro solar and economic development loop is an example of a balancing type of feedback loop; in other words when one increases, the other decreases, balancing out. Money in the project will go directly to economic development in the form of wages and the purchase of local supplies, thus supporting economic development. Having a project that enhances women’s livelihoods, bolsters carbon-free energy, and has shown sustained success elsewhere sufficiently demonstrates the positive feedbacks of the project. Increasing high prices of diesel fuel will force rural residents to cut back on their electricity use but solar micro-grid can help provide some of that electricity and fill the gap thanks to outside funding. Increased electricity from solar means businesses, schools, clinics and more can stay open longer hours, this means they can employ more people, therefore more money is available locally to purchase more solar systems.

The best 2 answers to explain the feedback loop that connects micro-solar and economic development are:

1. Money in the project will go directly to economic development in the form of wages and the purchase of local supplies, thus supporting economic development.
2. Increased electricity from solar means businesses, schools, clinics, and more can stay open longer hours, this means they can employ more people, therefore more money is available locally to purchase more solar systems.

To which all SDGs and specific targets within those SDGs, the above question possibly aligns with? Please justify your selection of SDGs

The above question, which involves the implementation of an Enhanced Rural Resilience (ERR) project that establishes decentralized solar energy systems, aligns with several Sustainable Development Goals (SDGs). The specific targets within these SDGs are justified below:

1. SDG 7 - Affordable and Clean Energy:
   - Target 7.1: By 2030, ensure universal access to affordable, reliable, and modern energy services. Justification: The ERR project aims to make solar energy accessible and affordable to rural communities.
   - Target 7.2: By 2030, increase substantially the share of renewable energy in the global energy mix. Justification: The project promotes the use of solar energy, a renewable energy source, contributing to a higher share of renewables in the energy mix.
2. SDG 5 - Gender Equality:
   - Target 5.5: Ensure women's full and effective participation and equal opportunities for leadership at all levels of decision-making in political, economic, and public life. Justification: The ERR project focuses on supporting women, empowering them through access to energy, and improving their livelihoods.
3. SDG 8 - Decent Work and Economic Growth:
   - Target 8.3: Promote development-oriented policies that support productive activities, decent job creation, entrepreneurship, creativity and innovation, and encourage the formalization and growth of micro-, small- and medium-sized enterprises, including through access to financial services. Justification: The project aims to stimulate rural economic development, create jobs, and support local businesses through the implementation of micro-solar energy systems.
4. SDG 13 - Climate Action:
   - Target 13.1: Strengthen resilience and adaptive capacity to climate-related hazards and natural disasters in all countries. Justification: By promoting clean, renewable energy, the project contributes to reducing greenhouse gas emissions, which in turn helps to mitigate climate change.

These are the primary SDGs and targets that align with the mentioned ERR-inspired project. The project's focus on providing clean energy, supporting women, and stimulating economic development in rural areas contributes to the overall global effort to achieve these SDGs.

Question 14 One complaint that you are accustomed to hearing about solar is that it costs too much upfront to install. In addition, this ERR project will be more expensive upfront between paying for trainings and employing previously marginalized beneficiaries on top of financing solar systems, and so on. This of course uses money that could otherwise be spent on projects with immediate benefits. Over decades of work, you have saved a substantial sum of money that you intended to use for charitable purposes when you retire. Your spouse suggests that you should instead spend the majority of it to get the ERR project off the ground. Imagine yourself in this situation. Which 2 statements best describe your EMOTIONS? Scared to put your money into a project that won’t yield immediate benefit given the uncertainty of the future. Confident that it is worth spending your savings on only if the financial return on investment of the solar systems is calculated to be high enough Apprehensive about using a sustainability assessment to decide where to put your own money as it includes values. Uncomfortable with the distributed nature of micro-solar which means you personally wouldn’t be able to as easily control and support its future development. Satisfied about the large if distant future benefits of this project (on multiple dimensions). Excited that you are able to make an investment that will improve the well-being of future generations.

The 2 statements that best describe your emotions in this situation are:

1. Satisfied about the large if distant future benefits of this project (on multiple dimensions).
2. Excited that you are able to make an investment that will improve the well-being of future generations.

To which all SDGs and specific targets within those SDGs, the above question possibly aligns with? Please justify your selection of SDGs

The above question focuses on the emotions related to investing personal funds in an Enhanced Rural Resilience (ERR) project. The question doesn't directly address specific Sustainable Development Goals (SDGs) or targets but can be indirectly linked to the following SDGs, as the underlying project aims to address them:

1. SDG 1 - No Poverty:
   - Target 1.4: By 2030, ensure that all men and women, in particular the poor and the vulnerable, have equal rights to economic resources, as well as access to basic services, ownership and control over land and other forms of property, inheritance, natural resources, appropriate new technology, and financial services, including microfinance. Justification: The ERR project could provide economic opportunities and access to clean energy, which can improve living conditions for poor and vulnerable communities.
2. SDG 4 - Quality Education:
   - Target 4.7: By 2030, ensure that all learners acquire the knowledge and skills needed to promote sustainable development, including, among others, through education for sustainable development and sustainable lifestyles, human rights, gender equality, promotion of a culture of peace and non-violence, global citizenship and appreciation of cultural diversity and of culture’s contribution to sustainable development. Justification: The ERR project includes training and capacity-building components that promote sustainable development and empower beneficiaries.
3. SDG 7 - Affordable and Clean Energy:
   - Target 7.1: By 2030, ensure universal access to affordable, reliable, and modern energy services. Justification: The project aims to make solar energy accessible and affordable to rural communities.
4. SDG 8 - Decent Work and Economic Growth:
   - Target 8.3: Promote development-oriented policies that support productive activities, decent job creation, entrepreneurship, creativity and innovation, and encourage the formalization and growth of micro-, small- and medium-sized enterprises, including through access to financial services. Justification: The project aims to stimulate rural economic development, create jobs, and support local businesses through the implementation of micro-solar energy systems.
5. SDG 13 - Climate Action:
   - Target 13.1: Strengthen resilience and adaptive capacity to climate-related hazards and natural disasters in all countries. Justification: By promoting clean, renewable energy, the project contributes to reducing greenhouse gas emissions, which in turn helps to mitigate climate change.

The emotions mentioned in the question are related to the satisfaction and excitement of potentially contributing to these SDGs through personal investment in the ERR project.

Question 15 You and the team you have built have been on the road for over a week, going from village to village, hosting meetings and reaching out to local stakeholders to build support for the project. At the last meeting, one of the local leaders protested that they had just bought a diesel generator and needed to be able to sell the electricity in order to pay off the purchase loan, so solar would not be welcomed. You snap at them that this is their own fault and that they are destroying the earth and their community’s health. You immediately feel bad and apologize but they don’t seem mollified. What actions would you take? Choose the best 2 answers. Keep up the hard work. You have gotten a lot done, and sometimes you have to make personal sacrifices of time in order to make projects work. Propose that you get paid more for this position given all the hours and acrimony that you are facing in the field. Decide that this village is not a good target for the ERR project as there is too much opposition. Focus on working with allies in the community, acknowledging that some people like the diesel generator owner are just going to stand in the way of progress. Develop with your team a strategy to engage generator owners in the project in a constructive fashion for both them and the project. Pause your work and reflect on whether you have over-worked yourself. Identify what self-care practices might be best for you to do right now, even though it will be slowing your work schedule.

The best 2 answers for actions to take in this situation are:

1. Develop with your team a strategy to engage generator owners in the project in a constructive fashion for both them and the project.
2. Focus on working with allies in the community, acknowledging that some people like the diesel generator owner are just going to stand in the way of progress.

To which all SDGs and specific targets within those SDGs, the above question possibly aligns with? Please justify your selection of SDGs

The above question revolves around addressing challenges and opposition encountered while promoting the Enhanced Rural Resilience (ERR) project in rural communities. It aligns with the following Sustainable Development Goals (SDGs) and specific targets:

1. SDG 7 - Affordable and Clean Energy:
   - Target 7.1: By 2030, ensure universal access to affordable, reliable, and modern energy services. Justification: The question focuses on promoting solar energy as a clean and affordable alternative to diesel generators in rural communities.
   - Target 7.2: By 2030, increase substantially the share of renewable energy in the global energy mix. Justification: The ERR project aims to establish decentralized solar energy systems, thus increasing the share of renewable energy.
2. SDG 11 - Sustainable Cities and Communities:
   - Target 11.3: By 2030, enhance inclusive and sustainable urbanization and capacity for participatory, integrated, and sustainable human settlement planning and management in all countries. Justification: Engaging with the community and addressing their concerns is an essential part of building sustainable and inclusive human settlements, which is part of the ERR project's objectives.
3. SDG 17 - Partnerships for the Goals:
   - Target 17.17: Encourage and promote effective public, public-private, and civil society partnerships, building on the experience and resourcing strategies of partnerships. Justification: The question emphasizes the importance of working with local stakeholders, including generator owners and community allies, to build partnerships for implementing the ERR project.

These SDGs and targets align with the question as it explores the challenges of engaging local stakeholders and the need for a strategic approach to overcome opposition while promoting sustainable energy solutions.

Question 16 You know that in crafting the strategy for implementing the ERR project you will want to develop an evidence-supported set of instructions for how to achieve the project vision. What additional elements should also be included in the strategy you develop? Choose the best 2 answers. Issues that might form barriers to success (e.g., views on roles of women) and what might be enabling/supporting factors (e.g., health clinics desiring reliable electricity). Villages that your team did not have a chance to contact yet but seem on paper similar to those that you visited. A plan that demands the project be completely self-financed within a five-year timeline. Utilizing a monochromatically increasing price of diesel fuel for the ten year planning timelines. Explicit tactics for managing the power dynamics around incumbent power producers (e.g., owners of diesel generator). Step by step instructions. These are largely sufficient, considering the limited resources, a theory of change for the project is not needed.

The best 2 additional elements to include in the strategy you develop are:

1. Issues that might form barriers to success (e.g., views on roles of women) and what might be enabling/supporting factors (e.g., health clinics desiring reliable electricity).
2. Explicit tactics for managing the power dynamics around incumbent power producers (e.g., owners of diesel generators).

To which all SDGs and specific targets within those SDGs, the above question possibly aligns with? Please justify your selection of SDGs

The above question focuses on developing a comprehensive strategy for implementing the Enhanced Rural Resilience (ERR) project, which includes addressing barriers, enabling factors, and power dynamics. The question aligns with the following Sustainable Development Goals (SDGs) and specific targets:

1. SDG 5 - Gender Equality:
   - Target 5.5: Ensure women’s full and effective participation and equal opportunities for leadership at all levels of decision-making in political, economic, and public life. Justification: The question mentions addressing views on the roles of women as a potential barrier to the project's success, which implies the need to promote gender equality.
2. SDG 7 - Affordable and Clean Energy:
   - Target 7.1: By 2030, ensure universal access to affordable, reliable, and modern energy services.
   - Target 7.2: By 2030, increase substantially the share of renewable energy in the global energy mix. Justification: The ERR project aims to establish decentralized solar energy systems in rural areas, contributing to SDG 7 targets.
3. SDG 10 - Reduced Inequalities:
   - Target 10.2: By 2030, empower and promote the social, economic, and political inclusion of all, irrespective of age, sex, disability, race, ethnicity, origin, religion or economic or other status. Justification: A well-crafted strategy for the ERR project should take into consideration the diverse needs and inclusion of different social groups.
4. SDG 17 - Partnerships for the Goals:
   - Target 17.14: Enhance policy coherence for sustainable development.
   - Target 17.17: Encourage and promote effective public, public-private, and civil society partnerships, building on the experience and resourcing strategies of partnerships. Justification: Developing an evidence-supported strategy that addresses various aspects of the project implementation process, including potential barriers and power dynamics, helps to establish effective partnerships and policy coherence.

By addressing barriers, enabling factors, and power dynamics in the strategy, the question emphasizes the importance of a comprehensive approach to implementing the ERR project, aligning with these SDGs and targets.

Question 17 The project is up and running and the first solar micro-grids are being installed. As part of your strategy, you built-in rapid assessments of the project. Which of the following actions would you take as part of the first rapid assessment? Choose the best 2 answers. Collect the data for future analysis and since you can’t change a strategy once it is implemented you can wait to assess the project until the end. Collect the data for the assessment using only data that is objective such as the amount of electricity generated which will be a sufficient data set for an assessment. Clarify the subject of assessment and determine what indicators you will be using to monitor success for this and future evaluations. Ask the implementors to quickly report back on whether they feel like the project is going successfully or not. Collect data on indicators that are outside the project’s direct influence such as changes in opening hours of health clinics, or changes in local students’ grades. Collect data on how many micro-grids have been installed and comparing that to your original goal will be a sufficient data set for an assessment.

The best 2 actions to take as part of the first rapid assessment are:

1. Clarify the subject of assessment and determine what indicators you will be using to monitor success for this and future evaluations.
2. Collect data on indicators that are outside the project’s direct influence such as changes in opening hours of health clinics, or changes in local students' grades.

To which all SDGs and specific targets within those SDGs, the above question possibly aligns with? Please justify your selection of SDGs

The above question focuses on conducting a rapid assessment for the Enhanced Rural Resilience (ERR) project, which involves the installation of solar micro-grids. The question aligns with the following Sustainable Development Goals (SDGs) and specific targets:

1. SDG 7 - Affordable and Clean Energy:
   - Target 7.1: By 2030, ensure universal access to affordable, reliable, and modern energy services.
   - Target 7.2: By 2030, increase substantially the share of renewable energy in the global energy mix. Justification: The ERR project aims to establish decentralized solar energy systems in rural areas, contributing to SDG 7 targets.
2. SDG 3 - Good Health and Well-being:
   - Target 3.8: Achieve universal health coverage, including financial risk protection, access to quality essential health-care services, and access to safe, effective, quality, and affordable essential medicines and vaccines for all. Justification: The question mentions collecting data on changes in opening hours of health clinics as part of the rapid assessment, which implies that the project might have an impact on health care accessibility.
3. SDG 4 - Quality Education:
   - Target 4.1: By 2030, ensure that all girls and boys complete free, equitable, and quality primary and secondary education leading to relevant and effective learning outcomes. Justification: The question refers to collecting data on changes in local students' grades as part of the assessment, indicating that the project might influence educational outcomes.
4. SDG 17 - Partnerships for the Goals:
   - Target 17.18: By 2020, enhance capacity-building support to developing countries, including for least developed countries and small island developing States, to increase significantly the availability of high-quality, timely, and reliable data. Justification: Conducting a rapid assessment and monitoring success indicators contributes to effective project management and supports capacity-building efforts for data-driven decision-making.

The question aligns with these SDGs and targets as it emphasizes the importance of monitoring and evaluating the ERR project's progress and impact on various aspects of sustainable development, such as energy access, health care, and education.

Question 18 Your initial assessment of the project roll-out is a mixed bag. One, particularly concerning finding, was that in several villages the solar micro-grids were being sabotaged and in other cases installation was even being blocked by groups of angry young men. What would you do with this information and the rest of the results of the assessment? Choose the best 2 answers. Avoid making changes until you have collected more data and can make statistically robust assessments of the situation. Re-evaluate your community engagement strategies and ask the expected women beneficiaries in the impacted communities, to wait with rolling out their micro-grids and prioritize male led groups. Do nothing for the moment. You very carefully developed your implementation plan, and it is too late to change now. Re-evaluate your community engagement strategies and identify new approaches which would enhance community acceptance. Ask what worked in the communities without issues. Continue the project with its focus on women and other disadvantaged populations, despite the additional challenges. Re-evaluate your community engagement strategies and identify the communities where roll-out has struggled and no longer continue the project in those places.

The best 2 actions to take with the information and the rest of the results of the assessment are:

1. Re-evaluate your community engagement strategies and identify new approaches which would enhance community acceptance. Ask what worked in the communities without issues.
2. Continue the project with its focus on women and other disadvantaged populations, despite the additional challenges.

To which all SDGs and specific targets within those SDGs, the above question possibly aligns with? Please justify your selection of SDGs

The above question focuses on addressing challenges faced during the implementation of the Enhanced Rural Resilience (ERR) project, specifically the sabotage and opposition to solar micro-grids installation. The question aligns with the following Sustainable Development Goals (SDGs) and specific targets:

1. SDG 5 - Gender Equality:
   - Target 5.1: End all forms of discrimination against all women and girls everywhere.
   - Target 5.5: Ensure women’s full and effective participation and equal opportunities for leadership at all levels of decision-making in political, economic, and public life. Justification: The question emphasizes the continuation of the project's focus on women and other disadvantaged populations, despite facing additional challenges.
2. SDG 7 - Affordable and Clean Energy:
   - Target 7.1: By 2030, ensure universal access to affordable, reliable, and modern energy services.
   - Target 7.2: By 2030, increase substantially the share of renewable energy in the global energy mix. Justification: The ERR project aims to establish decentralized solar energy systems in rural areas, contributing to SDG 7 targets.
3. SDG 16 - Peace, Justice, and Strong Institutions:
   - Target 16.7: Ensure responsive, inclusive, participatory, and representative decision-making at all levels.
   - Target 16.a: Strengthen relevant national institutions, including through international cooperation, for building capacity at all levels, in particular in developing countries, to prevent violence and combat terrorism and crime. Justification: The question highlights the need to re-evaluate community engagement strategies and adopt approaches that enhance community acceptance and promote peace and inclusivity.

The question aligns with these SDGs and targets, as it emphasizes the importance of adapting the project's implementation approach to address challenges and promote gender equality, access to clean energy, and inclusive community engagement.

On a recent visit with your aunt, she told you about this new organization she had joined called an Inter-generational Self-help Club (ISHC). She described how this inter-generational organization is empowered to design, conduct, and manage community development activities in partnership with NGOs and government agencies. You are particularly excited by how they focus on being inclusive. She has put you in contact with the sponsoring NGO and their representative told you that they would be very interested in supporting the creation of an ISHC in your community if you would be willing to take the lead.

Question 19 When you tell your friend about this opportunity, they ask if this is really your job as a random community member to organize? Shouldn’t the formation of an ISHC be left to the professionals? Imagine yourself in this situation. Which 2 statements best describe your EMOTIONS? Comfortable leading this initiative only if the supporting NGO tells me how it should be implemented based on their experiences elsewhere. Confident that you have all the knowledge, skills, and abilities to design and implement an ISHC on your own if you are given financial support. Excited by the idea of bringing together a diverse and inclusive group of people to tackle our community’s problems. Confident that despite the challenges, you can lead this project by building a strong team. Intimidated by the prospect of leading a complex sustainability project such as an ISHC and by all its participants and stakeholders. Apprehensive because complex sustainability projects should be led by professionals, not me.

Excited by the idea of bringing together a diverse and inclusive group of people to tackle our community’s problems. Confident that despite the challenges, you can lead this project by building a strong team.

To which all SDGs and specific targets within those SDGs, the above question possibly aligns with? Please justify your selection of SDGs

The question and the concept of an Inter-generational Self-help Club (ISHC) align with multiple Sustainable Development Goals (SDGs) as the initiative focuses on community development activities, inclusivity, and partnership with NGOs and government agencies. Some of the relevant SDGs and their targets include:

1. SDG 3: Good Health and Well-being Target 3.8: Achieve universal health coverage, including financial risk protection, access to quality essential health-care services, and access to safe, effective, quality, and affordable essential medicines and vaccines for all. The ISHC could promote health and well-being in the community by addressing health-related concerns.
2. SDG 4: Quality Education Target 4.7: Ensure that all learners acquire the knowledge and skills needed to promote sustainable development, including, among others, through education for sustainable development and sustainable lifestyles. The ISHC could support educational initiatives and foster lifelong learning among community members across generations.
3. SDG 10: Reduced Inequalities Target 10.2: Empower and promote the social, economic, and political inclusion of all, irrespective of age, sex, disability, race, ethnicity, origin, religion, or economic or other status. The ISHC focuses on inclusivity, which helps reduce inequalities within the community.
4. SDG 11: Sustainable Cities and Communities Target 11.3: Enhance inclusive and sustainable urbanization and capacity for participatory, integrated, and sustainable human settlement planning and management in all countries. The ISHC fosters community development activities that contribute to sustainable cities and communities.
5. SDG 16: Peace, Justice, and Strong Institutions Target 16.7: Ensure responsive, inclusive, participatory, and representative decision-making at all levels. The ISHC collaborates with NGOs and government agencies, promoting inclusive decision-making processes within the community.
6. SDG 17: Partnerships for the Goals Target 17.17: Encourage and promote effective public, public-private, and civil society partnerships, building on the experience and resourcing strategies of partnerships. The ISHC embodies this target by working in partnership with NGOs and government agencies to address community challenges.

In summary, the formation of an ISHC aligns with multiple SDGs and their specific targets, as it aims to foster community development, inclusivity, and partnerships to tackle various challenges.

Question 20 You and the NGO representative have a series of discussions about how to go about the process of implementing an ISHC. They share with you some of the different ways in which past communities have implemented their ISHCs. You really want the ISHC to be both successful and maximally transformative for your community and thus are looking to design the process carefully. Given this which of the following advice should you follow? Choose the best 2 answers. The first step of the process should be to clearly articulate the strategy for setting up the ISHC based on the successes that others have had. Integrated problem-solving is just about planning and developing strategies, the implementation is a separate and independent process. There is only one correct order of steps that will assure a successful outcome, it’s outlined in the ISHC guidebook. Including diverse voices in the process must be done even when it will slow down and generate conflicts during the process. You need to be an expert at each step of the process in order to be able to integrate and facilitate the process. It is important to develop a vision and an understanding of the current state before designing strategies to bridge the gap between them.

The first step of the process should be to clearly articulate the strategy for setting up the ISHC based on the successes that others have had. Including diverse voices in the process must be done even when it will slow down and generate conflicts during the process.

To which all SDGs and specific targets within those SDGs, the above question possibly aligns with? Please justify your selection of SDGs

The question, focusing on the implementation process of an Inter-generational Self-help Club (ISHC) to ensure its success and transformative impact on the community, aligns with several Sustainable Development Goals (SDGs) and their targets:

1. SDG 4: Quality Education Target 4.7: Ensure that all learners acquire the knowledge and skills needed to promote sustainable development, including, among others, through education for sustainable development and sustainable lifestyles. Designing and implementing an effective ISHC can promote education and lifelong learning in the community.
2. SDG 10: Reduced Inequalities Target 10.2: Empower and promote the social, economic, and political inclusion of all, irrespective of age, sex, disability, race, ethnicity, origin, religion, or economic or other status. Including diverse voices in the process is crucial to address inequalities and ensure inclusivity.
3. SDG 11: Sustainable Cities and Communities Target 11.3: Enhance inclusive and sustainable urbanization and capacity for participatory, integrated, and sustainable human settlement planning and management in all countries. An ISHC can contribute to sustainable and inclusive urban development by involving various community members and stakeholders.
4. SDG 16: Peace, Justice, and Strong Institutions Target 16.7: Ensure responsive, inclusive, participatory, and representative decision-making at all levels. By involving diverse voices in the planning and implementation process of an ISHC, it encourages inclusive decision-making within the community.
5. SDG 17: Partnerships for the Goals Target 17.17: Encourage and promote effective public, public-private, and civil society partnerships, building on the experience and resourcing strategies of partnerships. Collaborating with NGOs and drawing upon their experience to establish the ISHC exemplifies this target.

In summary, designing and implementing an effective and transformative ISHC process aligns with multiple SDGs and their specific targets, as it promotes education, inclusivity, sustainable urban development, inclusive decision-making, and partnerships.

Q21. In one of your conversations with a local health care provider, you explain that the ISHC intends to address multiple, complex socio-economic and environmental problems concurrently. The healthcare provider representative wonders why a multifunctional organization would produce better outcomes than a specialty-focused one such as themselves. How would you respond? Choose the best 2 answers. You agree with them that multiple specializations in each domain (such as health care clinics) offer the best solution to addressing sustainability problems. You explain that making durable, and effective change requires finding root causes and specifying these intervention points, an analysis which can rarely be done within the confines of a single domain. You assure them that your analysis will not include input from non-experts. You explain that taking a systems approach to problems is best, but it requires sophisticated modeling techniques which is why it is not done much. You agree that a systems analysis is too challenging because you have to identify every possible element and connection between them within the community no matter how trivial. You explain that there are feedback loops between different domains such as health and poverty and they can be more effectively addressed concurrently.

You explain that making durable, and effective change requires finding root causes and specifying these intervention points, an analysis which can rarely be done within the confines of a single domain. You explain that there are feedback loops between different domains such as health and poverty and they can be more effectively addressed concurrently.

To which all SDGs and specific targets within those SDGs, the above question possibly aligns with? Please justify your selection of SDGs

The question emphasizes the importance of a multifunctional organization like an Inter-generational Self-help Club (ISHC) in addressing complex socio-economic and environmental problems concurrently. This approach aligns with several Sustainable Development Goals (SDGs) and their targets:

1. SDG 1: No Poverty Target 1.2: Reduce at least by half the proportion of men, women, and children of all ages living in poverty in all its dimensions according to national definitions. Addressing feedback loops between domains like health and poverty can contribute to reducing poverty levels.
2. SDG 3: Good Health and Well-being Target 3.8: Achieve universal health coverage, including financial risk protection, access to quality essential health-care services, and access to safe, effective, quality, and affordable essential medicines and vaccines for all. Addressing health and well-being issues concurrently with other challenges can lead to more effective solutions.
3. SDG 10: Reduced Inequalities Target 10.2: Empower and promote the social, economic, and political inclusion of all, irrespective of age, sex, disability, race, ethnicity, origin, religion, or economic or other status. A multifunctional organization can better address inequalities by tackling interconnected issues.
4. SDG 11: Sustainable Cities and Communities Target 11.3: Enhance inclusive and sustainable urbanization and capacity for participatory, integrated, and sustainable human settlement planning and management in all countries. Concurrently addressing socio-economic and environmental problems contributes to sustainable urban development.
5. SDG 17: Partnerships for the Goals Target 17.14: Enhance policy coherence for sustainable development. A multifunctional organization like an ISHC can help achieve better policy coherence by addressing interconnected problems and coordinating efforts across different domains.

In summary, recognizing the interdependence between different domains and addressing complex issues concurrently through a multifunctional organization like an ISHC aligns with multiple SDGs and their specific targets, promoting comprehensive solutions for sustainable development.

Question 22 You and your team have gathered for a planning session for an upcoming workshop. The workshop will be attended by local partners, government representatives, and community members. The primary objective of the workshop is to develop a vision for your community in 2030. With that in hand, you will be able to design the role that your ISCH can play in making it happen. What are some things that you and your team should keep in mind when planning the visioning workshop? Choose the best 2 answers. To build a vision, most of the time should be given for participants to discuss all the problems they see with the way things are right now. You must prepare to elicit participants’ values and work with them to prioritize and negotiate the values as a core part of the vision. Don’t restrain the participant’s ideas, visions are meant for inspiration and do not have to have any grounding in reality. The main purpose of such a session is to extract from participants what they want in the future so whether they leave inspired or not is immaterial. You should either rigorously structure the workshop ahead of time or be prepared for substantial work afterwards to create a vision that is coherent out of all the ideas from participants. Sustainability visions should be based exclusively on scientific facts (e.g., climate change) not the values of individuals or groups.

You must prepare to elicit participants’ values and work with them to prioritize and negotiate the values as a core part of the vision. You should either rigorously structure the workshop ahead of time or be prepared for substantial work afterwards to create a vision that is coherent out of all the ideas from participants.

To which all SDGs and specific targets within those SDGs, the above question possibly aligns with? Please justify your selection of SDGs

The question focuses on planning a visioning workshop for the community, involving local partners, government representatives, and community members to design the role of the Inter-generational Self-help Club (ISHC). This approach aligns with several Sustainable Development Goals (SDGs) and their targets:

1. SDG 11: Sustainable Cities and Communities Target 11.3: Enhance inclusive and sustainable urbanization and capacity for participatory, integrated, and sustainable human settlement planning and management in all countries. The visioning workshop fosters participatory planning and engages the community in designing a sustainable future.
2. SDG 16: Peace, Justice, and Strong Institutions Target 16.7: Ensure responsive, inclusive, participatory, and representative decision-making at all levels. The workshop aims to include diverse perspectives, ensuring that the decision-making process is inclusive and representative of the community's values and aspirations.
3. SDG 17: Partnerships for the Goals Target 17.16: Enhance the global partnership for sustainable development, complemented by multi-stakeholder partnerships that mobilize and share knowledge, expertise, technology, and financial resources. The workshop brings together local partners, government representatives, and community members, exemplifying the spirit of multi-stakeholder partnerships.
4. SDG 17: Partnerships for the Goals Target 17.17: Encourage and promote effective public, public-private, and civil society partnerships, building on the experience and resourcing strategies of partnerships. The visioning workshop encourages collaboration and partnerships among different stakeholders for community development.

In summary, planning a visioning workshop for the community, involving various stakeholders, aligns with multiple SDGs and their specific targets, promoting participatory planning, inclusive decision-making, and effective partnerships for sustainable development.

Question 23 Almost everyone came away from the visioning workshop inspired. After some work you have crafted a wonderful narrative that brings together themes of intergenerational solidarity, healthy families, and prospering communities. You have already had several local partners, who were on the fence about participating, contact you to say that after the workshop they are excited to be fully onboard. What would be the key next steps to take? Choose the best 2 answers. Develop and solidify the new local partners as well as those who had previously committed into a supportive network which is dedicated to success. Revisit the current state analysis your team crafted and develop some strategies which would enable the ISHC to get the community from the present to the articulated future vision. Work to develop a foolproof implementation plan that will definitely achieve the vision. Develop your strategy based exclusively on your understanding of the current state; the vision served its purpose in inspiring stakeholders and getting them on board. If possible, avoid any discussions of the local politics and power dynamics in the strategic plans as it might threaten some potential partners. Take advantage of the good momentum to announce the launch of the ISHC in the community and open it up as soon as possible.

Develop and solidify the new local partners as well as those who had previously committed into a supportive network which is dedicated to success. Revisit the current state analysis your team crafted and develop some strategies which would enable the ISHC to get the community from the present to the articulated future vision.

To which all SDGs and specific targets within those SDGs, the above question possibly aligns with? Please justify your selection of SDGs

The question focuses on the next steps after a successful visioning workshop, aiming to solidify local partnerships and develop strategies to bridge the gap between the current state and the envisioned future for the community. This approach aligns with several Sustainable Development Goals (SDGs) and their targets:

1. SDG 11: Sustainable Cities and Communities Target 11.3: Enhance inclusive and sustainable urbanization and capacity for participatory, integrated, and sustainable human settlement planning and management in all countries. By revisiting the current state analysis and developing strategies, the ISHC can contribute to sustainable and inclusive urban development.
2. SDG 16: Peace, Justice, and Strong Institutions Target 16.7: Ensure responsive, inclusive, participatory, and representative decision-making at all levels. Involving various stakeholders and local partners in the process promotes inclusive and participatory decision-making.
3. SDG 17: Partnerships for the Goals Target 17.16: Enhance the global partnership for sustainable development, complemented by multi-stakeholder partnerships that mobilize and share knowledge, expertise, technology, and financial resources. Solidifying local partnerships and working together towards the community's vision exemplifies the spirit of multi-stakeholder partnerships.
4. SDG 17: Partnerships for the Goals Target 17.17: Encourage and promote effective public, public-private, and civil society partnerships, building on the experience and resourcing strategies of partnerships. Developing a supportive network of local partners fosters effective collaboration for community development.

In summary, the key next steps after a successful visioning workshop align with multiple SDGs and their specific targets, emphasizing the importance of local partnerships, strategic planning, and inclusive decision-making to achieve sustainable development goals in the community.

Question 24 You and your team have gathered sufficient funding to launch your community’s ISHC and to your surprise, you were asked to be hired on as its interim director (and only full-time employee) until the formal hiring process of the director position starts (to which you could apply, too). You have a lot of confidence in the implementation plan which you have developed but its success depends on the collaborative coordination of numerous local partners, government agencies, and community members, thus on their full buy-in of the plan. What would be some actions or practices that you would put in place to ensure successful implementation? Choose the best 2 answers. Avoid wasting resources on monitoring progress and interim objectives; rather invest resources to support meeting the end goals as articulated in the project vision. Keep all the various entities working on implementation separate from each other as much as possible so as to avoid any disagreements and assure that everyone is following the original plan. Don’t invest your limited time in community events as you have already elicited input during planning and don’t need to include stakeholders in the implementation. Take the needed time to resolve conflicts and disputes among team members even if it slows progress in the short term. Work to assure effective and compassionate communication both among team members and continuously with relevant stakeholders. Remind yourself that as long as the project team stays true to the original sustainability vision, how the plan is implemented is not materially important for its success.

Take the needed time to resolve conflicts and disputes among team members even if it slows progress in the short term. Work to assure effective and compassionate communication both among team members and continuously with relevant stakeholders.

To which all SDGs and specific targets within those SDGs, the above question possibly aligns with? Please justify your selection of SDGs

**Dataset - SULITEST**

**Scenario**: While the SDGs were conceived with national governments in mind, cities around the world have begun to pursue direct implementation of the SDGs. A key part of this process is committing formally via Voluntary Local Review (VLR). VLRs typically include aligning local strategy to the SDGs, facilitating local stakeholder engagement in tackling the SDGs, and submitting progress reports to the United Nations. The mayor of your city (population 250,000) announced last week that they will be carrying out just such a VLR and you have been brought on to lead the initiative.

**Q1:** While the announcement from the mayor is fresh on everyone’s mind you quickly organize the first VLR meeting with representatives from an array of city departments. Your hopes for quick progress are dashed when the meeting is derailed by arguing, side discussions, and other issues. Despite the tight deadline you realize that the next meeting has to be focused on effective teamwork in sustainability. Which of the following would you NOT include as practices your team will follow on this project? Choose the best 2 answers. Make commitments through the establishment of a shared vision with clear goals and be sure to follow through with them. Even in this digital era, making time for face-to-face contact within the team is critical for effective teamwork in sustainability. Always prioritize the completion of assigned tasks over resolving interpersonal issues during official team meetings. Appreciate and reward individual team members or sub-teams in addition to focusing on the whole team's progress. Actively seek out and implement tools whose use can help foster the collaborative teamwork desired and seek out additional training if necessary. Let the roles of each team member and team logistics emerge organically over time.

1. Always prioritize the completion of assigned tasks over resolving interpersonal issues during official team meetings.
2. Let the roles of each team member and team logistics emerge organically over time.

1a. To which all SDGs and specific targets within those SDGs, the above question possibly aligns with?

The above question primarily aligns with the following Sustainable Development Goals (SDGs) and specific targets:

1. SDG 11: Sustainable Cities and Communities
   - Target 11.3: By 2030, enhance inclusive and sustainable urbanization and capacity for participatory, integrated and sustainable human settlement planning and management in all countries.
2. SDG 17: Partnerships for the Goals
   - Target 17.16: Enhance the global partnership for sustainable development, complemented by multi-stakeholder partnerships that mobilize and share knowledge, expertise, technology, and financial resources, to support the achievement of the sustainable development goals in all countries, in particular developing countries.
   - Target 17.17: Encourage and promote effective public, public-private and civil society partnerships, building on the experience and resourcing strategies of partnerships.

The question indirectly relates to all 17 SDGs, as the effective teamwork and collaborative efforts mentioned in the question are essential for the successful implementation of all the SDGs at a local level.

1b. Which of the eight cross-cutting sustainability competencies like: Self-Awareness Collaboration Critical Thinking Anticipatory Integrated Problem Solving Normative Strategic Systems Thinking are addressed by this question

This question addresses several cross-cutting sustainability competencies:

1. Collaboration: The question highlights the importance of teamwork and collaboration in the context of implementing the SDGs at the city level. By focusing on effective teamwork practices, the team can work cohesively towards common goals.
2. Critical Thinking: Selecting the best practices that should NOT be included in the team's approach requires the ability to analyze different strategies and their potential impact on the effectiveness of the team.
3. Integrated Problem Solving: The question encourages the integration of different perspectives and practices to address the complex challenge of implementing the SDGs at a local level. Identifying practices that hinder effective teamwork can contribute to improved problem-solving.
4. Strategic: The question also emphasizes the importance of having a clear vision, goals, and roles within the team. By focusing on strategic planning and organization, the team can work more efficiently and effectively towards achieving the SDGs.
5. Systems Thinking: Although not explicitly mentioned in the question, systems thinking is implicitly involved in the process of selecting practices that contribute to effective teamwork. Recognizing the interdependence of team members and the importance of collaboration and communication is essential for a successful and sustainable project outcome.

Q2 For the first VLR, the decision was made to give a particular focus to SDG 13: Climate Action. Therefore, the environmental department is leading a comprehensive analysis of the current state of the city in terms of climate change. The objective of this analysis is to understand the most important ways in which the city contributes to climate change. This will include investigating what the structures, policies, values, and so on that influence the situation right now. How would you guide the team’s efforts? Choose the best 2 answers. Only include elements that can be measured and formally modeled (e.g., energy use). Hold-off until a later point on specifying solutions and strategies for achieving SDG 13. Avoid discussing anything related to other SDGs. Consult with other city departments such as housing and economic development. Focus only on how local climate policies affect the city and not national or regional levels. Identify and quantify every possible source of greenhouse gas emissions connected to the city.

1. Consult with other city departments such as housing and economic development: Collaborating with various city departments will provide valuable insights into the diverse aspects of the city's contribution to climate change. It is crucial to understand the interconnections and dependencies between different sectors to effectively address SDG 13.
2. Identify and quantify every possible source of greenhouse gas emissions connected to the city: In order to effectively address climate change, it is essential to gain a comprehensive understanding of the city's greenhouse gas emissions. By identifying and quantifying all possible sources, the team can prioritize areas of focus for future actions and develop data-driven strategies to reduce emissions in line with SDG 13.

2a. To which all SDGs and specific targets within those SDGs, this question possibly aligns with?

This question primarily aligns with SDG 13: Climate Action, as it focuses on understanding the city's contribution to climate change and carrying out a Voluntary Local Review (VLR) with an emphasis on climate action. However, since the question also suggests consulting with other city departments, such as housing and economic development, there could be potential connections with other SDGs and specific targets within those. Some possible alignments include:

1. SDG 7: Affordable and Clean Energy
   - Target 7.2: Increase the share of renewable energy in the global energy mix.
   - Target 7.3: Improve energy efficiency.
2. SDG 9: Industry, Innovation, and Infrastructure
   - Target 9.4: Upgrade infrastructure and retrofit industries to make them sustainable.
3. SDG 11: Sustainable Cities and Communities
   - Target 11.2: Provide access to safe, affordable, accessible, and sustainable transport systems.
   - Target 11.6: Reduce the adverse environmental impact of cities.
4. SDG 12: Responsible Consumption and Production
   - Target 12.2: Achieve sustainable management and efficient use of natural resources.
   - Target 12.4: Achieve environmentally sound management of chemicals and waste.

These are just a few examples, and there could be more alignments depending on the specific actions and policies implemented as a result of the VLR.

2b. Which of the eight cross-cutting sustainability competencies like: Self-Awareness Collaboration Critical Thinking Anticipatory Integrated Problem Solving Normative Strategic Systems Thinking are addressed by this question

This question addresses several cross-cutting sustainability competencies:

1. Collaboration: The question suggests consulting with other city departments such as housing and economic development, which emphasizes the importance of working together and collaborating with diverse stakeholders to address complex sustainability issues.
2. Integrated Problem Solving: The question focuses on understanding the city's contributions to climate change by considering different structures, policies, and values. This demonstrates an integrated approach to problem-solving that combines multiple aspects of sustainability.
3. Systems Thinking: Investigating the city's contribution to climate change requires an understanding of the complex interdependencies between various sectors, such as energy, transportation, and waste management. This calls for a systems thinking approach to grasp the broader picture and identify leverage points for change.
4. Anticipatory: The question emphasizes identifying and quantifying greenhouse gas emissions connected to the city, which reflects an anticipatory mindset. Understanding the current state of emissions can help anticipate future trends and potential impacts, allowing for proactive planning and decision-making.
5. Strategic: Although the question does not explicitly mention developing strategies, it implies that the analysis would inform future strategic planning for the city to address SDG 13 effectively. By understanding the current state of the city's contribution to climate change, the team can develop targeted strategies to achieve meaningful progress towards SDG 13.

Q3. A key part of the report that you are developing will include a VISION of what the city will look like in 2030 after making significant progress towards the SDGs. Which of the following are correct statements about the sustainability vision you will develop? Choose the best 2 answers. It should focus on sustainability solutions. It should be able to be enacted in whole immediately. It should be extrapolated directly from current trends. It should include values such as human rights. It should be inspirational and does not have to be grounded in reality. It should focus on current problems and the tactics for solving them.

The correct statements about the sustainability vision to be developed for the city in 2030 are:

1. It should include values such as human rights: The sustainability vision should be aligned with the core values of human rights, equity, and justice, which are central to the SDGs.
2. It should be inspirational and does not have to be grounded in reality: The sustainability vision should be aspirational, inspiring, and ambitious, challenging the city to push beyond current limits and strive for excellence. However, it should also be grounded in reality, with specific targets and indicators that can be realistically achieved through effective planning, collaboration, and action.

Therefore, statements such as "It should be able to be enacted in whole immediately," "It should focus on sustainability solutions," "It should focus on current problems and the tactics for solving them," and "It should be extrapolated directly from current trends" are not necessarily correct, as the sustainability vision should be comprehensive, forward-looking, and context-specific, considering the unique challenges and opportunities of the city and its residents.

3a. To which all SDGs and specific targets within those SDGs, this question possibly aligns with?

The question is related to SDG 11 - Sustainable Cities and Communities, which aims to make cities and human settlements inclusive, safe, resilient, and sustainable. The specific target within SDG 11 that the question aligns with is Target 11.6, which calls for cities to "reduce the adverse per capita environmental impact of cities, including by paying special attention to air quality, municipal and other waste management."

The question also aligns with SDG 16 - Peace, Justice and Strong Institutions, particularly Target 16.7, which calls for "responsive, inclusive, participatory and representative decision-making at all levels."

In addition, the question touches upon the importance of values such as human rights, equity, and justice, which are cross-cutting themes that are fundamental to achieving all SDGs.

3b. Which of the eight cross-cutting sustainability competencies like: Self-Awareness Collaboration Critical Thinking Anticipatory Integrated Problem Solving Normative Strategic Systems Thinking are addressed by this question

The question pertains to several of the eight cross-cutting sustainability competencies:

1. Collaboration: The question highlights the importance of stakeholder engagement and collaboration in achieving the SDGs at the local level.
2. Anticipatory: The question focuses on developing a sustainability vision for the future, which requires anticipating the potential challenges and opportunities that may arise and planning accordingly.
3. Integrated problem-solving: The question acknowledges that achieving the SDGs requires addressing multiple interconnected social, economic, and environmental issues in an integrated manner.
4. Strategic: The question requires developing a long-term vision and strategy for sustainable development, taking into account the specific context and needs of the city.
5. Systems thinking: The question calls for a holistic and systemic approach to sustainable development, recognizing the complex interrelationships and feedback loops between different elements of the city's social, economic, and environmental systems.

Q4. After several months of work everything seems to be falling apart around you. Several departments have stopped sending representatives to meetings, commercial interests are complaining loudly about how this whole thing is anti-growth, and a stakeholder engagement session was only attended by local homeowners furious that low-income housing might be proposed in their neighborhoods, among other issues. At the same time the mayor’s office is breathing down your neck to get this done yesterday. A colleague of yours suggests that you should give up the stakeholder input and visioning you have been working on; instead, you should work directly on the policy recommendation to the mayor. Imagine yourself in this situation. Which 2 statements best describe your EMOTIONS? Frustrated because you could do a better VLR if you didn’t have to engage so many different groups. Angry at all the people who seem to just want to stand in the way of progress. Empathetic to the concerns of the homeowners and interested in getting past their anger. Resigned and accepting of your colleague’s suggestion to just move on to the policy recommendation. Motivated to continue with the problem-solving process despite the challenges. Discouraged about the whole enterprise and ready to give up.

The two statements that best describe my emotions in this situation are:

1. Empathetic to the concerns of the homeowners and interested in getting past their anger.
2. Motivated to continue with the problem-solving process despite the challenges.

While it can be frustrating to face obstacles and setbacks, it is important to remain empathetic and understanding towards stakeholders who may have concerns or reservations about the VLR process. It is also important to stay motivated and committed to finding solutions to the challenges that arise, rather than giving up or sacrificing important stakeholder input in favor of expediency.

4a. To which all SDGs and specific targets within those SDGs, this question possibly aligns with?

This question aligns with several of the Sustainable Development Goals (SDGs) and their specific targets, including:

1. SDG 11: Sustainable Cities and Communities - Target 11.1 calls for "adequate and affordable housing and basic services for all" and Target 11.3 calls for "enhancing inclusive and sustainable urbanization and capacity for participatory, integrated and sustainable human settlement planning and management."
2. SDG 16: Peace, Justice and Strong Institutions - Target 16.7 calls for "responsive, inclusive, participatory and representative decision-making at all levels."
3. SDG 17: Partnerships for the Goals - Target 17.16 calls for "enhancing the global partnership for sustainable development, complemented by multi-stakeholder partnerships that mobilize and share knowledge, expertise, technology and financial resources."

This question highlights the challenges and opportunities associated with implementing the SDGs at the local level, particularly in the context of engaging diverse stakeholders and fostering participatory decision-making processes. It underscores the importance of addressing social, economic, and environmental issues in an integrated manner and building partnerships across sectors and actors to achieve sustainable development.

4b. Which of the eight cross-cutting sustainability competencies like: Self-Awareness Collaboration Critical Thinking Anticipatory Integrated Problem Solving Normative Strategic Systems Thinking are addressed by this question

The following cross-cutting sustainability competencies are addressed by this question:

1. Collaboration: The question highlights the importance of engaging diverse stakeholders in the Voluntary Local Review (VLR) process, and the challenges that can arise when some groups are resistant or opposed to proposed actions. Collaboration is essential to addressing these challenges and finding mutually beneficial solutions that promote sustainable development.
2. Critical Thinking: The question raises several complex and interrelated issues related to sustainable urban development, including the need for affordable housing, the potential trade-offs between economic growth and environmental sustainability, and the importance of participatory decision-making. Critical thinking is necessary to analyze these issues and evaluate potential solutions in a thoughtful and rigorous manner.
3. Integrated Problem Solving: The question recognizes that sustainable urban development requires an integrated approach that balances social, economic, and environmental concerns. The VLR process is designed to align local strategies with the SDGs and foster collaboration across different sectors and actors to achieve sustainable development.
4. Strategic Thinking: The question highlights the importance of developing a strategic approach to the VLR process, taking into account the perspectives and concerns of diverse stakeholders and identifying potential challenges and opportunities. A strategic approach can help to anticipate and address potential obstacles and foster more effective collaboration and decision-making.
5. Systems Thinking: The question recognizes that sustainable urban development is a complex and dynamic system that involves multiple interrelated factors and actors. Systems thinking is necessary to understand these complex relationships and identify potential leverage points for intervention to achieve sustainable development.

Q5. One day late in the evening you get a call from the mayor, who assures you that she personally believes in the process that you have set-up and will provide additional funding and take some heat if that’s what is needed to do the Voluntary Local Review correctly. With fresh wind in your sails, you focus on developing strategies for one of the biggest impact areas of your cities—the majority of residents who drive their private vehicles. Your team, with the input of an array of stakeholders, has developed a series of solutions which would make significant progress towards the sustainability vision articulated in the report. Besides infrastructure (e.g., bike lanes, more bus routes, etc.) what else needs to be part of your sustainability solution strategy? Choose the best 2 answers. Identify potential barriers to successful implementation and how they might be overcome. Describing the infrastructure solutions in detail is sufficient for a sustainability solution strategy. An extensive systematic analysis of the ways in which private vehicle travel exacerbates inequality and greenhouse gas emissions in general. Try to be exhaustively comprehensive with your strategy that it never needs to be revisited or modified regardless of obstacles implementation may face. Avoid focusing on power or politics which might hamper or support implementation to not “awaken sleeping dogs”. Articulate a theory of change to explain how the solutions proposed lead to achieving the SDGs.

Besides infrastructure solutions, two key components that should be part of the sustainability solution strategy for private vehicle travel in the city are:

1. Education and Awareness Campaigns: Education and awareness campaigns can play a critical role in promoting sustainable transportation choices, especially among those who have limited access to information or are resistant to change. These campaigns can include public outreach through social media, billboards, posters, and workshops to raise awareness of the negative impacts of private vehicle travel on the environment and society. They can also provide information on sustainable transportation alternatives, such as public transportation, biking, and walking.

Barriers to successful implementation: The main challenge in implementing education and awareness campaigns is reaching people who are not interested or engaged in sustainable transportation. To overcome this barrier, the city can partner with community groups, schools, and local businesses to disseminate information and promote the benefits of sustainable transportation. The city can also use creative messaging and incentives to encourage behavior change and provide support for those who are transitioning to sustainable transportation options.

1. Incentives and Disincentives: Incentives and disincentives can be used to encourage sustainable transportation choices and discourage private vehicle use. Examples of incentives include discounted public transit fares, free or reduced-cost bike share programs, and parking discounts for electric vehicles. Disincentives can include congestion pricing, parking fees for private vehicles, and higher registration fees for gas-guzzling vehicles.

Barriers to successful implementation: The main challenge in implementing incentives and disincentives is the potential for pushback from residents who rely on private vehicles for transportation. To overcome this barrier, the city can engage in extensive outreach and education efforts to explain the rationale behind these policies and their benefits. The city can also gradually phase in these policies over time to allow residents to adjust to the changes.

A comprehensive approach to private vehicle travel should also include a systematic analysis of the ways in which it exacerbates inequality and greenhouse gas emissions. This analysis can help identify specific areas where sustainable transportation policies can be most effective and highlight the importance of equity considerations in the implementation of these policies.

The theory of change for these solutions is that by providing education and incentives to encourage sustainable transportation choices and disincentives to discourage private vehicle use, the city can reduce the number of vehicles on the road, decrease greenhouse gas emissions, and promote social and environmental sustainability. This, in turn, will contribute to the achievement of the SDGs, particularly SDG 11 (Sustainable Cities and Communities) and SDG 13 (Climate Action).

5a. To which all SDGs and specific targets within those SDGs, this question possibly aligns with?

This question aligns with several SDGs and their associated targets, including:

1. SDG 11: Sustainable Cities and Communities, particularly Target 11.2, which aims to provide access to safe, affordable, and sustainable transport systems for all.
2. SDG 13: Climate Action, particularly Target 13.1, which aims to strengthen resilience and adaptive capacity to climate-related hazards and natural disasters.
3. SDG 4: Quality Education, particularly Target 4.7, which aims to ensure that all learners acquire the knowledge and skills needed to promote sustainable development.
4. SDG 5: Gender Equality, particularly Target 5.5, which aims to ensure women’s full and effective participation and equal opportunities for leadership at all levels of decision-making.
5. SDG 8: Decent Work and Economic Growth, particularly Target 8.4, which aims to improve resource efficiency in consumption and production and to decouple economic growth from environmental degradation.
6. SDG 9: Industry, Innovation and Infrastructure, particularly Target 9.1, which aims to develop quality, reliable, sustainable, and resilient infrastructure to support economic development and human well-being.

By promoting sustainable transportation options and reducing greenhouse gas emissions, the implementation of the Voluntary Local Review and associated strategies can contribute to the achievement of these SDGs and their associated targets.

5b. Which of the eight cross-cutting sustainability competencies like: Self-Awareness Collaboration Critical Thinking Anticipatory Integrated Problem Solving Normative Strategic Systems Thinking are addressed by this question

This question addresses several of the eight cross-cutting sustainability competencies, including:

1. Collaboration: The question involves developing strategies for sustainability with input from an array of stakeholders, highlighting the importance of collaboration in achieving sustainable outcomes.
2. Critical Thinking: The question requires a systematic analysis of the ways in which private vehicle travel exacerbates inequality and greenhouse gas emissions, demonstrating the importance of critical thinking in understanding complex sustainability challenges.
3. Integrated Problem Solving: The question involves developing a comprehensive strategy for addressing the negative impacts of private vehicle travel that takes into account multiple dimensions of sustainability, including environmental, social, and economic factors.
4. Normative: The question involves aligning local strategy with the SDGs, highlighting the importance of normative frameworks for guiding sustainable decision-making.
5. Systems Thinking: The question requires consideration of the complex and interconnected systems that contribute to private vehicle travel and its negative impacts, emphasizing the importance of systems thinking in addressing sustainability challenges.

Overall, this question underscores the need for a range of sustainability competencies in developing and implementing effective sustainability strategies.

Q6. You get home after a long drive and collapse into bed. The press conference announcing the release of your VLR was a resounding success with positive feedback pouring in from community groups, media, local politicians, and many others. You are ready to forget about it all, but something keeps nagging at the back of your mind. The city’s largest impact on the climate is emissions from private cars, one of which you drive up and back for well over an hour each day. Imagine yourself in this situation, which actions would you be most likely to take? Choose the best 2 answers. Look up the public transit options from your house to work but if they are more time consuming than your current commute not pursue it further Reflect on your feelings for several days, taking the opportunity to critically evaluate what has led you to personally have such big impact due to commuting. Not worry too much about it, your job is to help the city be more sustainable not change your own practices. That there is nothing you can do because as a parent raising a family you have to have a big enough house and you can only afford it in the suburbs distant from your work. Cancel meetings the next day and take time to apply the same calculations of impact from transportation to your own lifestyle that you just did to the whole city. Discuss your feelings with a colleague the next day and realize that your role as a change agent is through your job and your personal decisions are irrelevant.

The two actions that would be most likely to take are:

1. Reflect on your feelings for several days, taking the opportunity to critically evaluate what has led you to personally have such a big impact due to commuting.

Reflecting on your feelings and critically evaluating your personal impact is an important first step in making changes. This will allow you to better understand your own behavior and identify potential areas for improvement. By doing so, you can be a better advocate for sustainable transportation options and lead by example.

1. Look up the public transit options from your house to work but if they are more time consuming than your current commute not pursue it further.

While it is important to consider more sustainable transportation options, it is also important to recognize that everyone's circumstances are different. If the public transit options are significantly more time-consuming than your current commute, it may not be feasible for you to pursue them. However, it is still important to keep this information in mind and look for opportunities to use public transit when feasible.

6a. To which all SDGs and specific targets within those SDGs, this question possibly aligns with?

The question relates to several SDGs and targets, including:

1. SDG 11: Sustainable Cities and Communities - Target 11.6 aims to "reduce the adverse per capita environmental impact of cities, including by paying special attention to air quality and municipal and other waste management."
2. SDG 13: Climate Action - Target 13.3 aims to "improve education, awareness-raising and human and institutional capacity on climate change mitigation, adaptation, impact reduction and early warning."
3. SDG 3: Good Health and Well-being - Target 3.9 aims to "substantially reduce the number of deaths and illnesses from hazardous chemicals and air, water, and soil pollution and contamination."
4. SDG 8: Decent Work and Economic Growth - Target 8.4 aims to "improve progressively, through 2030, global resource efficiency in consumption and production and endeavour to decouple economic growth from environmental degradation."
5. SDG 12: Responsible Consumption and Production - Target 12.2 aims to "achieve the sustainable management and efficient use of natural resources."

Overall, the question highlights the importance of sustainable transportation, reducing greenhouse gas emissions, improving air quality, and promoting responsible consumption and production.

6b. Which of the eight cross-cutting sustainability competencies like: Self-Awareness Collaboration Critical Thinking Anticipatory Integrated Problem Solving Normative Strategic Systems Thinking are addressed by this question

The question touches upon several cross-cutting sustainability competencies, including:

1. Self-Awareness: The action of reflecting on one's own behavior and critically evaluating personal impact demonstrates self-awareness.
2. Collaboration: The Voluntary Local Review (VLR) initiative and stakeholder engagement mentioned in the question require collaboration among different groups and individuals to achieve sustainability goals.
3. Critical Thinking: The action of reflecting on one's own behavior and critically evaluating personal impact demonstrates critical thinking skills.
4. Anticipatory: The question implies a future-oriented approach, where the VLR initiative aims to align local strategies with the SDGs to achieve long-term sustainability goals.
5. Integrated Problem Solving: The VLR initiative mentioned in the question involves addressing complex sustainability challenges that require an integrated problem-solving approach.
6. Normative: The question touches upon the normative aspects of sustainability by highlighting the importance of reducing the adverse impact of cities on the environment and promoting responsible consumption and production.
7. Strategic: The VLR initiative mentioned in the question requires a strategic approach to align local strategies with the SDGs and achieve long-term sustainability goals.
8. Systems Thinking: The question highlights the importance of considering the interconnections and interdependence between different sustainability goals, such as reducing greenhouse gas emissions, improving air quality, and promoting responsible consumption and production. This requires a systems thinking approach to understand the complex relationships and feedback loops between different components of the system.

You are working for an NGO which has won a grant to help improve animal husbandry in a struggling rural region. The winning proposal was based on the concept of Community-Based Animal Health Workers (CAHW) a program deployed successfully in a neighbouring country. This program trained local community members in basic veterinary tasks, improved husbandry practices, and provided necessary supplies and equipment. You have been tasked with implementing this project.

Q7. After the initial excitement has worn off you are now feeling a lot of self-doubt. You are new to the targeted region and have only limited experience with animal husbandry in general. Then you remember that in your past work and studies, you have successfully designed and implemented other similarly structured projects such as one that trained community health workers to administer basic care. What are some initial actions which you would plan to take in light of your experiences with problem-solving that will help you gain initial perspective? Choose the best 2 answers. Begin implementation immediately, planning to learn by doing, and adjusting the project on the fly. Banish the self-doubt you are feeling reminding yourself that such reflections get in the way of the confidence you will need to manage this complex project. Identify key stakeholders and complementary experts who you set-up meetings and interviews with and who you hope to integrate into the entire planning process. It being your job, you take personal responsibility and get to work in the office carefully designing an implementation plan based only on your relevant expertise. For your planning process, you work with veterinarians in the capital city to first develop a strategic implementation plan. Reflect on your own strengths and explicitly identify deficits where you will need significant support (e.g., local expertise).

The two best initial actions that can be taken in light of the experiences with problem-solving are:

1. Identify key stakeholders and complementary experts who you set-up meetings and interviews with and who you hope to integrate into the entire planning process. This approach recognizes the value of collaborative and participatory planning. By engaging with key stakeholders and complementary experts, you can gain valuable insights into the specific context of the targeted region, its people, and their cultural norms and values. This can help you develop a more comprehensive understanding of the challenges facing animal husbandry in the region and how best to design and implement the CAHW program. Furthermore, involving these stakeholders can also help you build trust and establish a sense of ownership of the program among the local communities.
2. Reflect on your own strengths and explicitly identify deficits where you will need significant support (e.g., local expertise). This approach recognizes the importance of self-awareness and the ability to identify one's limitations. By reflecting on your own strengths and weaknesses, you can identify areas where you may need additional support, such as local expertise in animal husbandry. This can help you prioritize your efforts and allocate resources more effectively, ensuring that you are leveraging your strengths while also addressing any gaps in your expertise. Additionally, being transparent about your limitations can help build trust with key stakeholders by demonstrating your willingness to seek out and incorporate their expertise into the project.

7a. To which all SDGs and specific targets within those SDGs, this question possibly aligns with?

This question aligns primarily with the following SDGs:

1. SDG 1 - No Poverty: Improving animal husbandry can help alleviate poverty by providing a source of income for rural communities.
2. SDG 2 - Zero Hunger: Better animal husbandry practices can increase food security and nutrition for communities by improving the quality and quantity of animal products.
3. SDG 3 - Good Health and Well-being: Improving animal health can have a direct impact on human health, as animals are a source of food and livelihoods for many people.
4. SDG 5 - Gender Equality: By involving local community members in the CAHW program, the project can help promote gender equality by empowering women to participate in traditionally male-dominated roles.
5. SDG 8 - Decent Work and Economic Growth: The project can help create decent work opportunities for rural communities by promoting animal husbandry as a sustainable livelihood option.
6. SDG 12 - Responsible Consumption and Production: The project can promote responsible consumption and production by improving animal welfare and reducing the environmental impact of animal husbandry practices.

Specific targets within these SDGs that the project may align with include:

- SDG 1.1: By 2030, eradicate extreme poverty for all people everywhere, currently measured as people living on less than $1.25 a day.
- SDG 2.3: By 2030, double the agricultural productivity and incomes of small-scale food producers, in particular women, indigenous peoples, family farmers, pastoralists, and fishers, including through secure and equal access to land, other productive resources and inputs, knowledge, financial services, markets, and opportunities for value addition and non-farm employment.
- SDG 3.3: By 2030, end the epidemics of AIDS, tuberculosis, malaria and neglected tropical diseases and combat hepatitis, water-borne diseases and other communicable diseases.
- SDG 5.5: Ensure women's full and effective participation and equal opportunities for leadership at all levels of decision-making in political, economic and public life.
- SDG 8.3: Promote development-oriented policies that support productive activities, decent job creation, entrepreneurship, creativity and innovation, and encourage the formalization and growth of micro-, small- and medium-sized enterprises, including through access to financial services.
- SDG 12.3: By 2030, halve per capita global food waste at the retail and consumer levels and reduce food losses along production and supply chains, including post-harvest losses.

7b. Which of the eight cross-cutting sustainability competencies like: Self-Awareness Collaboration Critical Thinking Anticipatory Integrated Problem Solving Normative Strategic Systems Thinking are addressed by this question

Several of the eight cross-cutting sustainability competencies are addressed by this question, including:

1. Self-awareness: The question highlights the importance of self-awareness, as one of the initial actions recommended is to reflect on one's own strengths and explicitly identify deficits where significant support is needed. This demonstrates the importance of self-awareness in understanding one's own limitations and seeking out additional support to address these gaps.
2. Collaboration: The question emphasizes the importance of collaboration, as one of the initial actions recommended is to identify key stakeholders and complementary experts and involve them in the planning process. This highlights the need for collaboration and participatory planning to ensure the success of the project.
3. Critical thinking: The question requires critical thinking as it presents a complex problem and challenges the reader to identify the best initial actions to take in light of their problem-solving experiences.
4. Integrated problem-solving: The question requires an integrated problem-solving approach, as it involves multiple components of animal husbandry, including basic veterinary tasks, husbandry practices, and the provision of necessary supplies and equipment. The Community-Based Animal Health Workers (CAHW) program also requires an integrated approach that addresses the various challenges of animal husbandry in the targeted region.
5. Normative: The question highlights the importance of ethical considerations and normative values, as improving animal welfare and reducing the environmental impact of animal husbandry practices are important components of the project.
6. Strategic: The question requires a strategic approach, as one of the initial actions recommended is to work with veterinarians in the capital city to develop a strategic implementation plan. This highlights the need for a strategic approach to ensure the success of the project.

Overall, this question requires a comprehensive and integrated sustainability approach that emphasizes the importance of self-awareness, collaboration, critical thinking, anticipatory, normative, strategic, and systems thinking competencies to achieve the project's goals.

Q8: One of your first tasks will be determining which animal husbandry tasks should be promoted by the project. You discover through your research and consultation with experts that there has recently been a big outbreak of anthrax, and that no one is sure whether it was a one-off issue or the beginning of an ongoing problem for the region. Given this, how could you make informed decisions about selecting husbandry tasks to be promoted? Choose the best 2 answers. Run a collaborative brainstorming session to create an inspiring vision as your possible future scenario for analysis. Develop at least two scenarios of the future if anthrax returns on a regular basis and if it doesn’t. Pick a single trend (e.g., changing future temperatures) with which to evaluate how the future of anthrax outbreaks might play out. Analyze different scenarios to see which tasks would be best for CAHWs to promote for each. Rely primarily on the quantitative epidemiological models to forecast exactly what level of anthrax infections are to be expected. Study what has worked best in the past as the future is too uncertain to be of any use for making decisions. SDG 2: Zero Hunger - Selecting appropriate husbandry tasks to promote based on potential future scenarios contributes to the goal of achieving food security and promoting sustainable agriculture, as informed decisions can help maintain healthy livestock and enhance agricultural productivity. SDG 3: Good Health and Well-being - Addressing potential disease outbreaks, such as anthrax, by incorporating scenario analysis into the project can contribute to improved public health by reducing the risks associated with zoonotic diseases. SDG 8: Decent Work and Economic Growth - Training Community-Based Animal Health Workers (CAHWs) in tasks relevant to potential future scenarios supports job creation and sustainable economic growth in the region. SDG 15: Life on Land - Making informed decisions about animal husbandry tasks can contribute to sustainable land management and help maintain ecosystems and biodiversity by reducing the negative impacts of livestock on the environment. SDG 17: Partnerships for the Goals - The emphasis on stakeholder engagement, collaboration, and scenario analysis aligns with the goal of fostering partnerships to achieve sustainable development.

To make informed decisions about selecting husbandry tasks to be promoted, there are several approaches that could be taken. The best two answers are:

1. Run a collaborative brainstorming session to create an inspiring vision as your possible future scenario for analysis: This approach involves bringing together a diverse group of stakeholders, such as farmers, veterinarians, community leaders, and researchers, to brainstorm and develop a shared vision of the future of animal husbandry in the region. By exploring different scenarios of the future, including the possibility of recurring anthrax outbreaks, the group can identify the husbandry tasks that are most likely to promote healthy livestock and mitigate disease risks. This approach can help build consensus and ownership of the project among the community, which can increase the likelihood of its success.
2. Develop at least two scenarios of the future if anthrax returns on a regular basis and if it doesn’t. Pick a single trend (e.g., changing future temperatures) with which to evaluate how the future of anthrax outbreaks might play out. Analyze different scenarios to see which tasks would be best for CAHWs to promote for each: This approach involves using quantitative epidemiological models to forecast the potential level of anthrax infections based on different scenarios of the future. By examining the impact of different trends, such as changing temperatures, on the likelihood of anthrax outbreaks, the project team can identify the husbandry tasks that are most likely to mitigate the disease risks. This approach can help ensure that the project is evidence-based and that the selected tasks are most likely to have a positive impact on animal health.

Overall, selecting appropriate husbandry tasks to promote based on potential future scenarios is critical for achieving the Sustainable Development Goals (SDGs). Specifically, it can contribute to SDG 2 (Zero Hunger) by maintaining healthy livestock and enhancing agricultural productivity, SDG 3 (Good Health and Well-being) by reducing the risks associated with zoonotic diseases, SDG 8 (Decent Work and Economic Growth) by supporting job creation and sustainable economic growth, SDG 15 (Life on Land) by reducing the negative impacts of livestock on the environment, and SDG 17 (Partnerships for the Goals) by fostering partnerships to achieve sustainable development.

8a. To which all SDGs and specific targets within those SDGs, this question possibly aligns with?

This question aligns with the following Sustainable Development Goals (SDGs) and specific targets within those SDGs:

1. SDG 2: Zero Hunger
   - Target 2.3: By 2030, double the agricultural productivity and incomes of small-scale food producers, in particular women, indigenous peoples, family farmers, pastoralists, and fishers, including through secure and equal access to land, other productive resources and inputs, knowledge, financial services, markets, and opportunities for value addition and non-farm employment.
2. SDG 3: Good Health and Well-being
   - Target 3.3: By 2030, end the epidemics of AIDS, tuberculosis, malaria and neglected tropical diseases and combat hepatitis, water-borne diseases and other communicable diseases.
   - Target 3.8: Achieve universal health coverage, including financial risk protection, access to quality essential health-care services, and access to safe, effective, quality and affordable essential medicines and vaccines for all.
3. SDG 8: Decent Work and Economic Growth
   - Target 8.3: Promote development-oriented policies that support productive activities, decent job creation, entrepreneurship, creativity, and innovation, and encourage the formalization and growth of micro-, small- and medium-sized enterprises, including through access to financial services.
4. SDG 15: Life on Land
   - Target 15.1: By 2020, ensure the conservation, restoration, and sustainable use of terrestrial and inland freshwater ecosystems and their services, in particular forests, wetlands, mountains, and drylands, in line with obligations under international agreements.
5. SDG 17: Partnerships for the Goals
   - Target 17.16: Enhance the global partnership for sustainable development, complemented by multi-stakeholder partnerships that mobilize and share knowledge, expertise, technology and financial resources, to support the achievement of the sustainable development goals in all countries, in particular developing countries.

8b. Which of the eight cross-cutting sustainability competencies like: Self-Awareness Collaboration Critical Thinking Anticipatory Integrated Problem Solving Normative Strategic Systems Thinking are addressed by this question?

This question addresses the following cross-cutting sustainability competencies:

1. Anticipatory: The question involves anticipating potential future scenarios related to animal husbandry in the region, including the possibility of recurring anthrax outbreaks, and making informed decisions based on those scenarios.
2. Integrated Problem Solving: The question requires a holistic approach to solving the problem of improving animal husbandry in the region, by considering the interrelated social, economic, and environmental factors that influence the health of livestock and the sustainability of agriculture.
3. Strategic: The question involves developing a long-term strategy for improving animal husbandry in the region, by identifying the husbandry tasks that are most likely to promote healthy livestock and mitigate disease risks based on different future scenarios.
4. Collaboration: The question emphasizes the importance of stakeholder engagement and collaboration in developing a shared vision of the future of animal husbandry in the region, and in identifying the husbandry tasks that are most likely to have a positive impact on animal health.
5. Systems Thinking: The question requires a systems thinking approach to understanding the complex interactions between livestock, humans, and the environment, and to identifying the husbandry tasks that are most likely to have a positive impact on animal health and the sustainability of agriculture.

In summary, this question involves several cross-cutting sustainability competencies, including Anticipatory, Integrated Problem Solving, Strategic, Collaboration, and Systems Thinking.

Q9: In the funded proposal the project was described as “a cost-efficient model to improve the quality of animal health services and contribute to improved livelihood assets in rural populations.” You are now facing the task to operationalize these principles of cost-efficiency, quality of animal health services, and livelihood assets into concrete targets and indicators. Which of the following are important to the development of indicators for this project? Choose the best 2 answers. Include indicators focused on livestock health exclusively as that is the project’s purpose. Distinguish between success (e.g., improvement in access for women to veterinary services) and targets (e.g., gender parity in access) for each indicator. Organize focus groups or other approaches to gather input from diverse stakeholders regarding the development of indicators. Solicit only the input of experts with sufficient technical knowledge to dictate the development of the indicators. Develop indicators that can be easily measured on a cost benefit analysis with the threshold for success being “net benefits exceeding net costs”. Eliminate any indicators around human rights as these are “values” based and indicators should be value-free and “scientific”.

The best two answers to this question are:

1. Organize focus groups or other approaches to gather input from diverse stakeholders regarding the development of indicators.
2. Develop indicators that can be easily measured on a cost benefit analysis with the threshold for success being “net benefits exceeding net costs”.

Explanation: To operationalize the principles of cost-efficiency, quality of animal health services, and livelihood assets into concrete targets and indicators, it is important to engage diverse stakeholders in the development of indicators. This includes farmers, community members, local veterinarians, and other relevant actors. Their inputs and perspectives can provide valuable insights into the local context, which can be used to develop indicators that are relevant and meaningful to the target population.

Additionally, developing indicators that can be easily measured on a cost-benefit analysis is important to ensure that the project is financially sustainable and efficient. This can help to identify areas where the project is generating net benefits and areas where it may need to be improved. By setting a threshold for success as "net benefits exceeding net costs," the project can ensure that it is achieving its intended goals in a cost-effective manner.

It is important to note that eliminating any indicators around human rights because they are "values" based is not advisable. While indicators should be scientifically grounded, they should also reflect the ethical and moral values of the society they serve. It is essential to uphold human rights, especially in rural communities where individuals may already face various social and economic challenges. Moreover, indicators should focus on livestock health exclusively as the project's purpose is to improve animal husbandry practices and livestock health services.

9a. To which all SDGs and specific targets within those SDGs, this question possibly aligns with?

This question aligns with several Sustainable Development Goals (SDGs) and specific targets within those SDGs.

The SDGs that are particularly relevant to the project described in the question are:

1. SDG 1: No Poverty - Target 1.5: "By 2030, build the resilience of the poor and those in vulnerable situations and reduce their exposure and vulnerability to climate-related extreme events and other economic, social and environmental shocks and disasters."
2. SDG 2: Zero Hunger - Target 2.3: "By 2030, double the agricultural productivity and incomes of small-scale food producers, in particular women, indigenous peoples, family farmers, pastoralists and fishers, including through secure and equal access to land, other productive resources and inputs, knowledge, financial services, markets and opportunities for value addition and non-farm employment."
3. SDG 3: Good Health and Well-being - Target 3.3: "By 2030, end the epidemics of AIDS, tuberculosis, malaria and neglected tropical diseases and combat hepatitis, water-borne diseases and other communicable diseases."
4. SDG 5: Gender Equality - Target 5.a: "Undertake reforms to give women equal rights to economic resources, as well as access to ownership and control over land and other forms of property, financial services, inheritance and natural resources, in accordance with national laws."
5. SDG 8: Decent Work and Economic Growth - Target 8.5: "By 2030, achieve full and productive employment and decent work for all women and men, including for young people and persons with disabilities, and equal pay for work of equal value."
6. SDG 13: Climate Action - Target 13.1: "Strengthen resilience and adaptive capacity to climate-related hazards and natural disasters in all countries."
7. SDG 15: Life on Land - Target 15.2: "By 2020, promote the implementation of sustainable management of all types of forests, halt deforestation, restore degraded forests and substantially increase afforestation and reforestation globally."

The project aligns with these SDGs and their specific targets by addressing issues related to poverty reduction, hunger alleviation, improving animal health and well-being, promoting gender equality, creating employment opportunities, increasing resilience to climate change, and promoting sustainable land use.

9b. Which of the eight cross-cutting sustainability competencies like: Self-Awareness Collaboration Critical Thinking Anticipatory Integrated Problem Solving Normative Strategic Systems Thinking are addressed by this question?

This question addresses several of the cross-cutting sustainability competencies:

1. Collaboration: The implementation of the Community-Based Animal Health Workers (CAHW) program requires collaboration among various stakeholders, including community members, local veterinarians, and other relevant actors. This collaboration is necessary to ensure the success of the program and the sustainable improvement of animal health services in the rural region.
2. Critical thinking: To operationalize the principles of cost-efficiency, quality of animal health services, and livelihood assets into concrete targets and indicators, critical thinking skills are required. This includes the ability to analyze the available data, identify relevant indicators, and develop a plan that is both feasible and effective.
3. Anticipatory: The CAHW program is designed to address the current challenges in animal husbandry in the rural region, but it is also important to consider the future challenges that may arise. Therefore, an anticipatory approach is required to ensure that the program remains relevant and effective over time.
4. Integrated problem-solving: The CAHW program aims to address multiple challenges related to animal husbandry, including improving animal health services, promoting sustainable land use, and increasing the resilience of the rural community to climate change. Therefore, an integrated problem-solving approach is required to address these challenges comprehensively.
5. Strategic: The CAHW program requires a strategic approach to ensure that it is aligned with the goals and objectives of the project. This includes identifying the most effective strategies to improve animal health services, developing a plan for the deployment of Community-Based Animal Health Workers, and establishing a system to monitor and evaluate the program's progress.
6. Systems thinking: The CAHW program is a complex system that involves various stakeholders and multiple interconnected factors. Therefore, a systems thinking approach is required to understand the relationships between these factors and how they impact the success of the program.

Overall, this question addresses several of the cross-cutting sustainability competencies, including collaboration, critical thinking, anticipatory, integrated problem-solving, strategic, and systems thinking.

Q10: After weeks of work and coordination, you have set up a full-day workshop with a diverse set of stakeholders—local politicians, livestock farmers, religious leaders, and more. While not completely balanced, there was a reasonable proportion of women participants as well. The discussions, activities, and interactive sessions were going well. You are collecting lots of very valuable information and perspectives that you would not have been able to obtain otherwise, such as challenges to consistently delivering supplies in certain districts. During the lunch break, you and your team take a chance to review the workshop and make any adjustments. One of your female colleagues points out that not a single of the woman participants has spoken in any of the public sessions. Another colleague responds that we did our job getting them there and we can’t force the women to speak up. Imagine yourself in this situation. Which 2 statements best describe your EMOTIONS? Glad there is a woman on your team as that is their job to catch such omissions. Disappointed not to have heard from the women participants but satisfied that the workshop has enabled their physical presence. Disconcerted that among the whole team (including yourself) only one member noticed the issue and feeling a need to reflect on the implications of this. Unconcerned about who specifically has not spoken up at the workshop as they probably just didn’t have anything they felt was important enough to say. In strong agreement with your colleague that you need to change things up so as to hear the voices of the women participants. Frustrated with the women participants for not speaking up and putting the team in this bind.

The two statements that best describe my emotions in this situation are:

1. Disconcerted that among the whole team (including yourself) only one member noticed the issue and feeling a need to reflect on the implications of this.
2. In strong agreement with your colleague that you need to change things up so as to hear the voices of the women participants.

As someone working for an NGO, it is important to ensure that all voices are heard and that everyone is given an equal opportunity to participate in discussions and activities. The fact that none of the women participants have spoken up during the workshop is a cause for concern, and it is important to reflect on why this might be the case and how to address it. At the same time, I am glad that there is a woman on my team who was able to bring this issue to our attention. Working together, we can make changes to ensure that the voices of all participants are heard and that the workshop is as effective as possible in achieving its goals.

10a. To which all SDGs and specific targets within those SDGs, this question possibly aligns with?

The question relates to the following SDGs and specific targets:

1. SDG 1: No Poverty

- Target 1.4: Ensure that all men and women, in particular, the poor and the vulnerable, have equal rights to economic resources, as well as access to basic services, ownership, and control over land and other forms of property, inheritance, natural resources, appropriate new technology, and financial services including microfinance.

1. SDG 2: Zero Hunger

- Target 2.3: By 2030, double the agricultural productivity and incomes of small-scale food producers, in particular, women, indigenous peoples, family farmers, pastoralists, and fishers, including through secure and equal access to land, other productive resources and inputs, knowledge, financial services, markets, and opportunities for value addition and non-farm employment.

1. SDG 5: Gender Equality

- Target 5.5: Ensure women’s full and effective participation and equal opportunities for leadership at all levels of decision-making in political, economic, and public life.

1. SDG 8: Decent Work and Economic Growth

- Target 8.3: Promote development-oriented policies that support productive activities, decent job creation, entrepreneurship, creativity and innovation, and encourage the formalization and growth of micro-, small- and medium-sized enterprises, including through access to financial services.

1. SDG 10: Reduced Inequalities

- Target 10.2: By 2030, empower and promote the social, economic, and political inclusion of all, irrespective of age, sex, disability, race, ethnicity, origin, religion or economic or other status.

Overall, the question aligns with several SDGs and targets that aim to promote equality, empower marginalized groups, and improve access to basic resources and services for all. Specifically, it highlights the importance of ensuring that women have equal rights, opportunities, and participation in decision-making processes related to animal husbandry and agriculture.

10b. Which of the eight cross-cutting sustainability competencies like: Self-Awareness Collaboration Critical Thinking Anticipatory Integrated Problem Solving Normative Strategic Systems Thinking are addressed by this question?

The question addresses several of the eight cross-cutting sustainability competencies.

1. Collaboration: The implementation of the Community-Based Animal Health Workers (CAHW) program involves collaborating with a diverse set of stakeholders, including local politicians, livestock farmers, and religious leaders. The workshop discussed in the question also involves collaboration among team members to identify areas of improvement for the program.
2. Critical Thinking: The female colleague who noticed that none of the women participants had spoken up during the workshop demonstrates critical thinking by recognizing an issue and bringing it to the attention of the team. The team members' discussion and reflection on this issue also involve critical thinking to identify potential solutions.
3. Self-Awareness: The fact that the female colleague was able to identify the lack of participation by women during the workshop demonstrates self-awareness of her own biases and awareness of potential barriers to women's participation in discussions.
4. Strategic Systems Thinking: The implementation of the CAHW program and the workshop discussed in the question involves thinking about the larger system of animal husbandry in the rural region and identifying potential areas for improvement that can have a long-term impact.

Overall, the question addresses several of the sustainability competencies, including collaboration, critical thinking, self-awareness, and strategic systems thinking.

Q11. You and your team have developed three alternative implementation strategies based on the integrated problem-solving process you have gone through. You will need to decide which strategy the project will take in establishing a network of CAHWs. In choosing among these strategies which of the following actions would you take to assure eventual success? Choose the best 2 answers. Solicit the support of local organizations such as the national veterinary services for one or more of the strategies. Await the results of a third academic study on the original CAHW project to be absolutely sure of the science before moving forward with implementing any strategy. Assess which strategy is likely to have the greatest impact on poverty as this is the only metric in which a quantitative analysis can be conducted. Analyze how each of the strategies would play out if applied to your original understanding of the current, problematic situation. Determine which strategy would improve animal health the fastest even though that would mean less training on other improved husbandry practices. Calculate the total cost of each strategy and choose the least expensive option.

The two best answers would be to solicit the support of local organizations such as the national veterinary services for one or more of the strategies and to analyze how each of the strategies would play out if applied to your original understanding of the current, problematic situation.

Soliciting the support of local organizations such as the national veterinary services is important because they can provide valuable resources, expertise, and credibility to the project. This can help build trust with the local community, as well as ensure that the project is sustainable and integrated with existing systems.

Analyzing how each of the strategies would play out if applied to your original understanding of the current, problematic situation is also crucial. This will allow you to assess which strategy is most relevant and feasible in the local context. It is important to consider factors such as the availability of resources, the local culture, and the existing knowledge and skills of community members. By doing so, you can increase the chances of success and ensure that the project meets the needs of the community.

The other options are not as effective. Awaiting the results of a third academic study would delay the project unnecessarily and may not provide any new insights. Assessing which strategy is likely to have the greatest impact on poverty as the only metric is limited and does not take into account other important factors. Determining which strategy would improve animal health the fastest, even though that would mean less training on other improved husbandry practices, could have negative long-term consequences and reduce the sustainability of the project. Calculating the total cost of each strategy and choosing the least expensive option may not take into account the long-term benefits and impact of each strategy.

11a. To which all SDGs and specific targets within those SDGs, this question possibly aligns with?

This question aligns with several of the Sustainable Development Goals (SDGs), including:

1. SDG 1: No Poverty - This question seeks to improve animal husbandry in a struggling rural region, which can help improve the livelihoods of the local community and reduce poverty.
2. SDG 2: Zero Hunger - By improving animal husbandry practices, this project can help increase the availability and accessibility of food for the local community.
3. SDG 3: Good Health and Well-being - The project aims to train local community members in basic veterinary tasks, which can improve the health and well-being of both the animals and the community.
4. SDG 8: Decent Work and Economic Growth - By improving animal husbandry practices, this project can create new economic opportunities for the local community and support sustainable economic growth.
5. SDG 12: Responsible Consumption and Production - The project aims to promote sustainable and responsible animal husbandry practices, which can help reduce the environmental impact of animal agriculture.

Specific targets within these SDGs that this question may align with include:

- SDG 1.2: By 2030, reduce at least by half the proportion of men, women and children of all ages living in poverty in all its dimensions according to national definitions.
- SDG 2.3: By 2030, double the agricultural productivity and incomes of small-scale food producers, in particular women, indigenous peoples, family farmers, pastoralists and fishers, including through secure and equal access to land, other productive resources and inputs, knowledge, financial services, markets and opportunities for value addition and non-farm employment.
- SDG 3.3: By 2030, end the epidemics of AIDS, tuberculosis, malaria and neglected tropical diseases and combat hepatitis, water-borne diseases and other communicable diseases.
- SDG 8.5: By 2030, achieve full and productive employment and decent work for all women and men, including for young people and persons with disabilities, and equal pay for work of equal value.
- SDG 12.3: By 2030, halve per capita global food waste at the retail and consumer levels and reduce food losses along production and supply chains, including post-harvest losses.

11b. Which of the eight cross-cutting sustainability competencies like: Self-Awareness Collaboration Critical Thinking Anticipatory Integrated Problem Solving Normative Strategic Systems Thinking are addressed by this question?

This question addresses several of the eight cross-cutting sustainability competencies:

1. Collaboration: The question involves working with local organizations and community members to implement a program that addresses animal husbandry in a rural region. Collaboration is necessary to ensure that the project meets the needs of the local community and is sustainable.
2. Integrated Problem Solving: The question specifically mentions that an integrated problem-solving process was used to develop alternative implementation strategies. This competency involves taking a holistic approach to problem-solving and considering multiple perspectives and factors.
3. Normative: The question involves improving animal husbandry practices, which can have positive ethical implications for the treatment of animals and the environment.
4. Systems Thinking: The question involves improving animal husbandry practices in a rural region, which requires an understanding of the complex social, economic, and environmental systems that impact animal agriculture in that region. Systems thinking involves considering the interconnections between these different systems.

Overall, this question demonstrates the importance of applying sustainability competencies to real-world situations in order to achieve sustainable development goals.

Q12: It has been several months since your implementation plan was rolled out and things seem to be going very well. Your local implementing partners have sent back enthusiastic reports of success. On several phone calls, they have told you that they have trained more CAHWs than was anticipated and that local reception for them has been very positive. This is great news because you have a video conference with the funders early the next morning (due to differing time zones). Just then your email pings with the quarterly monitoring reports. Which of the following would you do? Choose the best 2 answers. Conduct a quick, descriptive analysis of the monitoring report data even though it gets in the way of working on your presentation. Continue to develop the presentation for the funders as the priority is to get it highly polished. Reflect on the reliability of the phone-based conversations as the exclusive basis for analysis. Base your opinion about the success of the project on the verbal reports you have as the callers are in the field and you are not. Rely on what you heard over the phone even if the monitoring reports conflict with what you were told. Work to convey your excitement about the positive reports from your implementing partners into the presentation.

The best two answers are:

1. Conduct a quick, descriptive analysis of the monitoring report data even though it gets in the way of working on your presentation.
2. Work to convey your excitement about the positive reports from your implementing partners into the presentation.

It is important to review the monitoring report data to ensure that the project is meeting its objectives and to identify any areas that may need improvement. While the presentation is important, it should not be prioritized over the need to analyze the data and make any necessary adjustments to the project.

Additionally, it is important to acknowledge the positive feedback from the implementing partners in the presentation. However, it is important to also present any data from the monitoring reports that may contradict or provide additional context to the verbal reports received over the phone. This will help provide a more comprehensive understanding of the project's progress and success.

12a. To which all SDGs and specific targets within those SDGs, this question possibly aligns with?

This question possibly aligns with the following SDGs and specific targets:

1. SDG 1: No Poverty - Target 1.5: Build resilience of the poor and those in vulnerable situations
2. SDG 2: Zero Hunger - Target 2.3: Double the agricultural productivity and incomes of small-scale food producers
3. SDG 3: Good Health and Well-being - Target 3.3: End the epidemics of AIDS, tuberculosis, malaria, and neglected tropical diseases
4. SDG 8: Decent Work and Economic Growth - Target 8.5: Achieve full and productive employment and decent work for all, including women and young people
5. SDG 10: Reduced Inequalities - Target 10.2: Empower and promote the social, economic and political inclusion of all, irrespective of age, sex, disability, race, ethnicity, origin, religion or economic or other status

The implementation of Community-Based Animal Health Workers (CAHW) can help improve animal husbandry practices, which can increase agricultural productivity and incomes of small-scale food producers, contributing to SDG 2. This can also improve the health and well-being of the local community and help build resilience among the poor and those in vulnerable situations, contributing to SDG 1 and SDG 3.

Furthermore, by training local community members in basic veterinary tasks and providing necessary supplies and equipment, the program can contribute to the achievement of full and productive employment and decent work for all, including women and young people, under SDG 8. The program can also help promote the social, economic, and political inclusion of all, irrespective of age, sex, disability, race, ethnicity, origin, religion, or economic or other status, contributing to SDG 10.

12b. Which of the eight cross-cutting sustainability competencies like: Self-Awareness Collaboration Critical Thinking Anticipatory Integrated Problem Solving Normative Strategic Systems Thinking are addressed by this question?

This question potentially addresses the following cross-cutting sustainability competencies:

1. Collaboration: The implementation of the Community-Based Animal Health Workers program involves working with local implementing partners and training local community members in basic veterinary tasks. This requires collaboration and coordination among various stakeholders.
2. Critical thinking: The question involves analyzing the monitoring report data to ensure that the project is meeting its objectives and identifying any areas that may need improvement. This requires critical thinking skills.
3. Anticipatory: The project aims to improve animal husbandry practices in a struggling rural region, which can have long-term benefits for the community's economic, social, and environmental sustainability. This requires anticipatory thinking, which involves considering the long-term impacts of the project.
4. Integrated problem solving: The project involves addressing complex issues related to animal husbandry practices, veterinary care, and community development. This requires an integrated approach to problem-solving that considers the interdependencies between different issues and stakeholders.
5. Strategic thinking: The project aims to improve animal husbandry practices in a struggling rural region, which requires a strategic approach to planning and implementation.
6. Systems thinking: The project involves addressing complex systems related to animal husbandry practices, veterinary care, and community development. This requires a systems thinking approach, which involves understanding the interconnections and interdependencies between different systems.

Overall, the implementation of the Community-Based Animal Health Workers program requires a range of sustainability competencies, including collaboration, critical thinking, anticipatory thinking, integrated problem solving, strategic thinking, and systems thinking

Several years ago, you founded a solar company that provides solar systems to businesses and households in the capital city. Recently you read about an award-winning Enhanced Rural Resilience (ERR) project that was implemented abroad and whose primary objective was to establish decentralized solar energy systems. But the program did more than just seek to make solar energy accessible and affordable, it also focused on supporting women, improving employability skills, and more as part of an integrated program. You feel that your company could spark such an initiative in your own country.

Q13. The best opportunity to fund this project is a recently launched funding mechanism for a public-private partnership that seeks to stimulate rural economic development. Your proposed ERR-inspired project is a finalist, and you are invited to give a presentation to the award panel. During your presentation, you are getting skeptical looks and finally one of the panelists says, “I can see how micro-solar power is a good thing, but I don’t see how it would stimulate economic development.” You respond by explaining the feedback loop that connects micro-solar and economic development using which of the following statements? Choose the best 2 answers. Consistency of solar electricity price as compared to the current diesel generated means that households and businesses can make long term financial plans, including the purchase of financed micro-solar which increases the ratio of electricity coming from stably priced solar in the future. The micro solar and economic development loop is an example of a balancing type of feedback loop; in other words when one increases, the other decreases, balancing out. Money in the project will go directly to economic development in the form of wages and the purchase of local supplies, thus supporting economic development. Having a project that enhances women’s livelihoods, bolsters carbon-free energy, and has shown sustained success elsewhere sufficiently demonstrates the positive feedbacks of the project. Increasing high prices of diesel fuel will force rural residents to cut back on their electricity use but solar micro-grid can help provide some of that electricity and fill the gap thanks to outside funding. Increased electricity from solar means businesses, schools, clinics and more can stay open longer hours, this means they can employ more people, therefore more money is available locally to purchase more solar systems.

The best statements to explain the feedback loop that connects micro-solar and economic development are:

1. Consistency of solar electricity price as compared to the current diesel generated means that households and businesses can make long term financial plans, including the purchase of financed micro-solar which increases the ratio of electricity coming from stably priced solar in the future. This statement highlights how the availability of solar energy at a consistent and stable price enables households and businesses to make long-term financial plans, including the purchase of financed micro-solar systems. This, in turn, increases the ratio of electricity coming from solar in the future, which has a positive impact on the overall economic development.
2. Money in the project will go directly to economic development in the form of wages and the purchase of local supplies, thus supporting economic development. This statement emphasizes how the project can directly contribute to economic development by providing wages to local residents and by purchasing supplies locally. By doing so, the project injects money into the local economy, which stimulates economic growth and development.

13a. To which all SDGs and specific targets within those SDGs, this question possibly aligns with?

The proposed ERR-inspired project aligns with several Sustainable Development Goals (SDGs) and specific targets within those SDGs, including:

1. SDG 1: No Poverty - by providing affordable and accessible solar energy, the project can help reduce poverty and promote economic development in rural areas.
2. SDG 5: Gender Equality - by focusing on enhancing women's livelihoods, the project can promote gender equality and empower women.
3. SDG 7: Affordable and Clean Energy - the project aligns directly with this goal by promoting the use of clean and sustainable energy sources.
4. SDG 8: Decent Work and Economic Growth - by stimulating economic development in rural areas, the project can help promote decent work and economic growth.
5. SDG 10: Reduced Inequalities - by promoting access to clean energy in rural areas, the project can help reduce inequalities and promote inclusive development.
6. SDG 13: Climate Action - by promoting the use of clean energy sources, the project can contribute to efforts to mitigate climate change.

Specific targets within these SDGs that the project could align with include:

- Target 1.4: By 2030, ensure that all men and women, in particular, the poor and the vulnerable, have equal rights to economic resources, as well as access to basic services, ownership and control over land and other forms of property, inheritance, natural resources, appropriate new technology and financial services, including microfinance.
- Target 5.A: Undertake reforms to give women equal rights to economic resources, as well as access to ownership and control over land and other forms of property, financial services, inheritance, and natural resources, in accordance with national laws.
- Target 7.1: By 2030, ensure universal access to affordable, reliable, and modern energy services.
- Target 8.5: By 2030, achieve full and productive employment and decent work for all women and men, including for young people and persons with disabilities, and equal pay for work of equal value.
- Target 10.2: By 2030, empower and promote the social, economic, and political inclusion of all, irrespective of age, sex, disability, race, ethnicity, origin, religion or economic or other status.
- Target 13.2: Integrate climate change measures into national policies, strategies, and planning.

13b. Which of the eight cross-cutting sustainability competencies like: Self-Awareness Collaboration Critical Thinking Anticipatory Integrated Problem Solving Normative Strategic Systems Thinking are addressed by this question?

This question addresses several cross-cutting sustainability competencies, including:

1. Anticipatory Thinking: The proposed ERR-inspired project involves anticipating future challenges and providing access to sustainable and clean energy sources in rural areas. This demonstrates an anticipatory thinking approach to address the challenges of poverty, inequality, and climate change.
2. Integrated Problem Solving: The project aims to address multiple challenges simultaneously, including access to clean energy, poverty reduction, gender inequality, and economic development. This demonstrates an integrated problem-solving approach to sustainability challenges.
3. Systems Thinking: The project considers the interconnectedness of various systems, such as energy, social, economic, and environmental systems. This demonstrates a systems thinking approach to sustainability challenges.
4. Collaboration: The project involves collaboration between multiple stakeholders, including the public and private sectors, to address sustainability challenges. This demonstrates a collaborative approach to sustainability challenges.
5. Critical Thinking: The project involves critical thinking to assess the feasibility and effectiveness of the proposed solution to address sustainability challenges.
6. Normative: The project aligns with normative sustainability principles, such as promoting access to clean and sustainable energy, reducing poverty and inequality, and empowering women.

Overall, this question demonstrates the importance of integrating various sustainability competencies to address complex sustainability challenges, such as access to clean energy, poverty reduction, and economic development in rural areas.

Q14. One complaint that you are accustomed to hearing about solar is that it costs too much upfront to install. In addition, this ERR project will be more expensive upfront between paying for trainings and employing previously marginalized beneficiaries on top of financing solar systems, and so on. This of course uses money that could otherwise be spent on projects with immediate benefits. Over decades of work, you have saved a substantial sum of money that you intended to use for charitable purposes when you retire. Your spouse suggests that you should instead spend the majority of it to get the ERR project off the ground. Imagine yourself in this situation. Which 2 statements best describe your EMOTIONS? Scared to put your money into a project that won’t yield immediate benefit given the uncertainty of the future. Confident that it is worth spending your savings on only if the financial return on investment of the solar systems is calculated to be high enough Apprehensive about using a sustainability assessment to decide where to put your own money as it includes values. Uncomfortable with the distributed nature of micro-solar which means you personally wouldn’t be able to as easily control and support its future development. Satisfied about the large if distant future benefits of this project (on multiple dimensions). Excited that you are able to make an investment that will improve the well-being of future generations.

The two statements that best describe your emotions are:

1. Scared to put your money into a project that won’t yield immediate benefit given the uncertainty of the future.
2. Excited that you are able to make an investment that will improve the well-being of future generations.

On one hand, you are hesitant to invest a substantial amount of money into a project that may not yield immediate benefits, given the uncertain future. On the other hand, you are excited about the potential long-term benefits that the ERR project could bring to future generations, which motivates you to take the risk and invest your savings.

14a. To which all SDGs and specific targets within those SDGs, this question possibly aligns with?

The question aligns with several SDGs and specific targets within those SDGs, including:

1. SDG 7 - Affordable and Clean Energy
   - Target 7.1: By 2030, ensure universal access to affordable, reliable and modern energy services
   - Target 7.2: By 2030, increase substantially the share of renewable energy in the global energy mix
2. SDG 5 - Gender Equality
   - Target 5.5: Ensure women's full and effective participation and equal opportunities for leadership at all levels of decision-making in political, economic, and public life
3. SDG 8 - Decent Work and Economic Growth
   - Target 8.3: Promote development-oriented policies that support productive activities, decent job creation, entrepreneurship, creativity, and innovation, and encourage the formalization and growth of micro-, small- and medium-sized enterprises, including through access to financial services
4. SDG 10 - Reduced Inequalities
   - Target 10.2: By 2030, empower and promote the social, economic, and political inclusion of all, irrespective of age, sex, disability, race, ethnicity, origin, religion, or economic or other status
5. SDG 13 - Climate Action
   - Target 13.2: Integrate climate change measures into national policies, strategies, and planning
6. SDG 17 - Partnerships for the Goals
   - Target 17.16: Enhance the global partnership for sustainable development, complemented by multi-stakeholder partnerships that mobilize and share knowledge, expertise, technology, and financial resources, to support the achievement of the sustainable development goals in all countries

The ERR project aligns with these SDGs and targets by promoting the use of renewable energy, supporting the inclusion of marginalized individuals, promoting job creation and entrepreneurship, reducing inequalities, integrating climate change measures, and encouraging partnerships for sustainable development.

14b. Which of the eight cross-cutting sustainability competencies like: Self-Awareness Collaboration Critical Thinking Anticipatory Integrated Problem Solving Normative Strategic Systems Thinking are addressed by this question?

The question addresses several of the eight cross-cutting sustainability competencies, including:

1. Anticipatory thinking: The question requires thinking ahead and considering the long-term impacts of the ERR project on future generations, as well as the potential risks and uncertainties that may arise.
2. Integrated problem-solving: The ERR project aims to address multiple challenges at once, including improving access to clean energy, supporting women and marginalized individuals, and promoting economic growth. This requires a holistic and integrated approach to problem-solving.
3. Normative: The question touches on normative issues related to values and ethics, as the decision to invest in the ERR project involves considering the broader social and environmental implications of the project, beyond just financial returns.
4. Collaboration: The ERR project involves working with various stakeholders, including communities, women's groups, and training providers, to ensure the project's success. Collaboration is essential to achieving the project's goals.

Overall, the question demonstrates the need for sustainability competencies such as anticipatory thinking, integrated problem-solving, normative considerations, and collaboration to address complex sustainability challenges and achieve sustainable development goals.

Q15. You and the team you have built have been on the road for over a week, going from village to village, hosting meetings and reaching out to local stakeholders to build support for the project. At the last meeting, one of the local leaders protested that they had just bought a diesel generator and needed to be able to sell the electricity in order to pay off the purchase loan, so solar would not be welcomed. You snap at them that this is their own fault and that they are destroying the earth and their community’s health. You immediately feel bad and apologize but they don’t seem mollified. What actions would you take? Choose the best 2 answers. Keep up the hard work. You have gotten a lot done, and sometimes you have to make personal sacrifices of time in order to make projects work. Propose that you get paid more for this position given all the hours and acrimony that you are facing in the field. Decide that this village is not a good target for the ERR project as there is too much opposition. Focus on working with allies in the community, acknowledging that some people like the diesel generator owner are just going to stand in the way of progress. Develop with your team a strategy to engage generator owners in the project in a constructive fashion for both them and the project. Pause your work and reflect on whether you have over-worked yourself. Identify what self-care practices might be best for you to do right now, even though it will be slowing your work schedule.

The two best actions to take in this scenario would be:

1. Develop with your team a strategy to engage generator owners in the project in a constructive fashion for both them and the project: This approach will help to address the concerns of the local leader and other generator owners in the community. It will also show them that the solar project is not meant to harm their business, but rather to offer an alternative that is cleaner, more sustainable, and cost-effective in the long run. The strategy could involve education, dialogue, and partnership building to find mutually beneficial solutions that meet the needs of everyone involved.
2. Pause your work and reflect on whether you have over-worked yourself. Identify what self-care practices might be best for you to do right now, even though it will be slowing your work schedule: It is important to take care of oneself when working on a project that involves interacting with stakeholders who may have opposing views. By taking a break and engaging in self-care practices, such as exercise, meditation, or spending time with loved ones, one can recharge and return to the work with a clear mind and renewed energy. This will ultimately lead to better decision-making and more effective project implementation.

15a. To which all SDGs and specific targets within those SDGs, this question possibly aligns with?

The question aligns with several SDGs and specific targets within those SDGs, including:

1. SDG 7 - Affordable and Clean Energy: This question aligns with SDG 7, which aims to ensure access to affordable, reliable, sustainable, and modern energy for all. Specifically, the Enhanced Rural Resilience (ERR) project, which promotes decentralized solar energy systems, aims to increase access to clean and affordable energy in rural areas, which can contribute to reducing poverty and improving health and education outcomes.
2. SDG 5 - Gender Equality: The ERR project also aligns with SDG 5, which aims to achieve gender equality and empower all women and girls. By supporting women as part of an integrated program, the project can help to improve their economic opportunities, increase their decision-making power, and promote gender equality.
3. SDG 8 - Decent Work and Economic Growth: The ERR project aligns with SDG 8, which aims to promote sustained, inclusive, and sustainable economic growth, full and productive employment, and decent work for all. By promoting the use of solar energy systems, the project can create new job opportunities and enhance the employability skills of people in rural areas.
4. SDG 13 - Climate Action: This question aligns with SDG 13, which aims to take urgent action to combat climate change and its impacts. By promoting the use of solar energy systems and reducing reliance on fossil fuels, the ERR project can contribute to reducing greenhouse gas emissions and mitigating the effects of climate change.
5. SDG 17 - Partnerships for the Goals: The question also aligns with SDG 17, which aims to strengthen the means of implementation and revitalize the global partnership for sustainable development. The ERR project involves building partnerships with local stakeholders to promote sustainable development in rural areas, which can contribute to achieving the other SDGs.

15b. Which of the eight cross-cutting sustainability competencies like: Self-Awareness Collaboration Critical Thinking Anticipatory Integrated Problem Solving Normative Strategic Systems Thinking are addressed by this question?

This question addresses several cross-cutting sustainability competencies, including:

1. Collaboration: The question highlights the importance of building support and working with local stakeholders to implement the Enhanced Rural Resilience (ERR) project. Collaboration is necessary to ensure that the project meets the needs of the community and to address concerns from local leaders and generator owners.
2. Critical thinking: The question requires critical thinking to develop a strategy that addresses the concerns of the local leader who protested the solar project. The strategy should be constructive and find mutually beneficial solutions that meet the needs of everyone involved.
3. Anticipatory: The question also requires an anticipatory approach to address the potential opposition that may arise in the implementation of the ERR project. By developing a strategy to engage generator owners in the project, the team can anticipate potential barriers and mitigate their impacts.
4. Integrated problem solving: The ERR project is an integrated program that not only seeks to make solar energy accessible and affordable but also focuses on supporting women, improving employability skills, and more. The project takes an integrated problem-solving approach that addresses multiple sustainability challenges simultaneously.
5. Normative: The question highlights the importance of norms in the community, including the need to acknowledge and work with opposing views. The approach taken should be respectful and considerate of the norms and values of the community.
6. Strategic: The question requires a strategic approach to ensure that the ERR project is implemented effectively and sustainably. This includes developing a strategy to engage generator owners, building partnerships with local stakeholders, and ensuring that the project meets the needs of the community.
7. Systems thinking: The question highlights the importance of systems thinking in addressing complex sustainability challenges. The ERR project is a complex system that involves multiple stakeholders, including women, generator owners, and the community. Systems thinking is necessary to ensure that the project is implemented effectively and that it achieves its intended outcomes.

In summary, this question requires a range of cross-cutting sustainability competencies to be addressed effectively, including collaboration, critical thinking, anticipatory, integrated problem solving, normative, strategic, and systems thinking

Q16. You know that in crafting the strategy for implementing the ERR project you will want to develop an evidence-supported set of instructions for how to achieve the project vision. What additional elements should also be included in the strategy you develop? Choose the best 2 answers. Issues that might form barriers to success (e.g., views on roles of women) and what might be enabling/supporting factors (e.g., health clinics desiring reliable electricity). Villages that your team did not have a chance to contact yet but seem on paper similar to those that you visited. A plan that demands the project be completely self-financed within a five-year timeline. Utilizing a monochromatically increasing price of diesel fuel for the ten year planning timelines. Explicit tactics for managing the power dynamics around incumbent power producers (e.g., owners of diesel generator). Step by step instructions. These are largely sufficient, considering the limited resources, a theory of change for the project is not needed.

The best two elements that should be included in the strategy for implementing the ERR project are:

1. Issues that might form barriers to success (e.g., views on roles of women) and what might be enabling/supporting factors (e.g., health clinics desiring reliable electricity): It is important to identify potential challenges or barriers to success that could hinder the achievement of the project goals, such as societal views on the roles of women, which could impact their participation and involvement in the project. Additionally, identifying enabling factors such as a demand for reliable electricity in health clinics can help to facilitate the success of the project.
2. Explicit tactics for managing the power dynamics around incumbent power producers (e.g., owners of diesel generators): As the ERR project seeks to establish decentralized solar energy systems, it is important to manage the power dynamics around incumbent power producers such as owners of diesel generators. This can involve identifying and addressing potential challenges or resistance from these producers, as well as developing strategies to work with them collaboratively to achieve project goals.

It is important to note that while step-by-step instructions are helpful, a theory of change for the project is also important in order to understand how the project is expected to achieve its goals and to guide decision-making and resource allocation. Additionally, a plan that demands the project be completely self-financed within a five-year timeline may not be realistic, particularly if the project is focused on supporting vulnerable communities or promoting social and environmental impact. Therefore, alternative financing strategies may need to be considered to ensure the sustainability of the project. Utilizing a monochromatically increasing price of diesel fuel for the ten-year planning timeline may also not accurately reflect market conditions and could be subject to significant variability. Finally, identifying and targeting specific villages based on their similarity to those that have already been visited can be a helpful approach, but it is important to also consider factors such as local community engagement and support when selecting target communities.

16a. To which all SDGs and specific targets within those SDGs, this question possibly aligns with

The question aligns with the following Sustainable Development Goals (SDGs):

1. SDG 7 - Affordable and Clean Energy: The question focuses on the implementation of decentralized solar energy systems to provide affordable and clean energy to businesses and households, which aligns with SDG 7.
2. SDG 5 - Gender Equality: The question also touches on the importance of supporting women, which aligns with SDG 5. The strategy for implementing the ERR project should identify potential barriers to success, such as societal views on the roles of women, and incorporate measures to empower women and promote gender equality.
3. SDG 8 - Decent Work and Economic Growth: The question mentions improving employability skills, which aligns with SDG 8. The ERR project could potentially create job opportunities and promote economic growth in the communities where it is implemented.
4. SDG 3 - Good Health and Well-being: The question mentions the importance of reliable electricity in health clinics, which aligns with SDG 3. Access to reliable energy sources can improve the quality of healthcare and support good health and well-being.

Specific targets within these SDGs that the question aligns with include:

- SDG 7.2 - By 2030, increase substantially the share of renewable energy in the global energy mix.
- SDG 5.5 - Ensure women's full and effective participation and equal opportunities for leadership at all levels of decision-making in political, economic and public life.
- SDG 8.5 - By 2030, achieve full and productive employment and decent work for all women and men, including for young people and persons with disabilities, and equal pay for work of equal value.
- SDG 3.8 - Achieve universal health coverage, including financial risk protection, access to quality essential health-care services and access to safe, effective, quality and affordable essential medicines and vaccines for all.

16b. Which of the eight cross-cutting sustainability competencies like: Self-Awareness Collaboration Critical Thinking Anticipatory Integrated Problem Solving Normative Strategic Systems Thinking are addressed by this question

The question addresses the following cross-cutting sustainability competencies:

1. Anticipatory: The question involves considering potential barriers to success, identifying enabling factors, and developing strategies to manage power dynamics, which all require an anticipatory approach to problem-solving.
2. Integrated Problem Solving: The question involves developing an evidence-supported set of instructions for how to achieve the project vision, which requires an integrated approach to problem-solving that considers multiple perspectives and factors.
3. Strategic: The question involves developing a strategy for implementing the ERR project, which requires a strategic approach to decision-making that considers long-term goals and sustainability.
4. Systems Thinking: The question involves implementing decentralized solar energy systems and managing power dynamics around incumbent power producers, which requires a systems thinking approach that considers the interconnectedness of various components and factors.
5. Collaboration: The question mentions the importance of supporting women, improving employability skills, and more as part of an integrated program, which requires collaboration with various stakeholders and partners.

Overall, the question demonstrates a need for a holistic and integrated approach to sustainability that requires the application of multiple competencies such as critical thinking, collaboration, and self-awareness to achieve the goals of the ERR project.

Q17. The project is up and running and the first solar micro-grids are being installed. As part of your strategy, you built-in rapid assessments of the project. Which of the following actions would you take as part of the first rapid assessment? Choose the best 2 answers. Collect the data for future analysis and since you can’t change a strategy once it is implemented you can wait to assess the project until the end. Collect the data for the assessment using only data that is objective such as the amount of electricity generated which will be a sufficient data set for an assessment. Clarify the subject of assessment and determine what indicators you will be using to monitor success for this and future evaluations. Ask the implementors to quickly report back on whether they feel like the project is going successfully or not. Collect data on indicators that are outside the project’s direct influence such as changes in opening hours of health clinics, or changes in local students’ grades. Collect data on how many micro-grids have been installed and comparing that to your original goal will be a sufficient data set for an assessment.

The two best actions to take as part of the first rapid assessment are:

1. Clarify the subject of assessment and determine what indicators you will be using to monitor success for this and future evaluations. This action is crucial as it will help define the scope and objectives of the assessment, as well as identify the indicators that will be used to measure the success of the project. By doing this, the assessment will be more targeted and focused on the key areas that need to be evaluated.
2. Collect data on indicators that are outside the project’s direct influence such as changes in opening hours of health clinics, or changes in local students’ grades. This action is important because it will help to determine the impact of the project on the broader community beyond just the installation of micro-grids. By collecting data on indicators that are outside the direct influence of the project, the assessment will provide a more comprehensive understanding of the project's impact on the community.

Therefore, the two best actions to take as part of the first rapid assessment are to clarify the subject of assessment and determine the indicators to monitor success, and to collect data on indicators that are outside the project’s direct influence.

17a. To which all SDGs and specific targets within those SDGs, this question possibly aligns with

The question is about establishing a decentralized solar energy system that supports women and improves employability skills, among other objectives. Therefore, this initiative aligns with the following SDGs and specific targets within those SDGs:

1. SDG 7: Affordable and Clean Energy
   - Target 7.1: By 2030, ensure universal access to affordable, reliable and modern energy services
   - Target 7.2: By 2030, increase substantially the share of renewable energy in the global energy mix
2. SDG 5: Gender Equality
   - Target 5.5: Ensure women’s full and effective participation and equal opportunities for leadership at all levels of decision-making in political, economic, and public life.
3. SDG 8: Decent Work and Economic Growth
   - Target 8.3: Promote development-oriented policies that support productive activities, decent job creation, entrepreneurship, creativity, and innovation, and encourage the formalization and growth of micro-, small- and medium-sized enterprises, including through access to financial services.
4. SDG 4: Quality Education
   - Target 4.4: By 2030, substantially increase the number of youth and adults who have relevant skills, including technical and vocational skills, for employment, decent jobs, and entrepreneurship.

Overall, the initiative aligns with several SDGs and specific targets within those SDGs, including SDG 7, 5, 8, and 4.

17b. Which of the eight cross-cutting sustainability competencies like: Self-Awareness Collaboration Critical Thinking Anticipatory Integrated Problem Solving Normative Strategic Systems Thinking are addressed by this question?

The question addresses the following cross-cutting sustainability competencies:

1. Collaboration: The question involves establishing a solar energy system that supports women, improves employability skills, and more. Such a project would require collaboration among various stakeholders, such as the solar company, local communities, government agencies, and NGOs, to achieve the desired outcomes.
2. Anticipatory: The question also involves rapid assessments of the project, which would require anticipating potential challenges or problems that may arise during the implementation process.
3. Systems Thinking: The question requires considering the broader social, economic, and environmental systems within which the solar energy system will operate, and how the system will impact these systems.
4. Normative: The question also involves considering ethical and social responsibility issues related to the project, such as ensuring equal access to energy for all members of the community and promoting gender equality.

Therefore, the cross-cutting sustainability competencies addressed by this question include Collaboration, Anticipatory, Systems Thinking, and Normative

Q18. Your initial assessment of the project roll-out is a mixed bag. One, particularly concerning finding, was that in several villages the solar micro-grids were being sabotaged and in other cases installation was even being blocked by groups of angry young men. What would you do with this information and the rest of the results of the assessment? Choose the best 2 answers. Avoid making changes until you have collected more data and can make statistically robust assessments of the situation. Re-evaluate your community engagement strategies and ask the expected women beneficiaries in the impacted communities, to wait with rolling out their micro-grids and prioritize male led groups. Do nothing for the moment. You very carefully developed your implementation plan, and it is too late to change now. Re-evaluate your community engagement strategies and identify new approaches which would enhance community acceptance. Ask what worked in the communities without issues. Continue the project with its focus on women and other disadvantaged populations, despite the additional challenges. Re-evaluate your community engagement strategies and identify the communities where roll-out has struggled and no longer continue the project in those places.

The best answers are:

1. Re-evaluate your community engagement strategies and identify new approaches which would enhance community acceptance. Ask what worked in the communities without issues.
2. Continue the project with its focus on women and other disadvantaged populations, despite the additional challenges.

It is important to address the issue of sabotage and resistance to installation in some villages. However, it is premature to avoid making changes until more data is collected since this issue may continue to escalate and cause more damage. Similarly, waiting to roll out micro-grids to women beneficiaries impacted by the situation may not address the underlying issue and may result in further delays in the project's implementation.

Doing nothing and assuming that the implementation plan is perfect is also not a viable option. It is crucial to re-evaluate the community engagement strategies and identify new approaches that can enhance community acceptance. This can involve understanding what worked well in the communities without issues and using that as a model for implementation in communities where the project has struggled.

It is also important to continue with the project's focus on women and other disadvantaged populations, despite the additional challenges. The project's objectives are not just limited to providing solar energy but also include supporting women and improving employability skills, and other areas. Stopping the project in areas where roll-out has struggled would mean abandoning the communities that most need the support of the project.

18a. To which all SDGs and specific targets within those SDGs, this question possibly aligns with?

The question aligns with several SDGs and specific targets within those SDGs.

1. SDG 7: Affordable and Clean Energy - The project aims to provide solar energy to households and businesses, thereby promoting affordable and clean energy.
2. SDG 5: Gender Equality - The project also focuses on supporting women, improving employability skills, and more as part of an integrated program, promoting gender equality.
3. SDG 8: Decent Work and Economic Growth - The project can contribute to creating job opportunities in the renewable energy sector.
4. SDG 10: Reduced Inequalities - By focusing on women and other disadvantaged populations, the project can help reduce inequalities.
5. SDG 11: Sustainable Cities and Communities - The project can contribute to making cities and communities more sustainable by promoting the use of renewable energy.
6. SDG 13: Climate Action - The project promotes the use of renewable energy, which can help mitigate climate change.

The specific targets within these SDGs that the project aligns with will depend on the specific objectives and outcomes of the project, as well as the context in which it is implemented.

18b. Which of the eight cross-cutting sustainability competencies like: Self-Awareness Collaboration Critical Thinking Anticipatory Integrated Problem Solving Normative Strategic Systems Thinking are addressed by this question?

This question addresses several cross-cutting sustainability competencies, including:

1. Critical Thinking - The question requires critical thinking to evaluate the results of the assessment and determine the best course of action to address the issue of sabotage and resistance to installation in some villages.
2. Collaboration - The question highlights the importance of community engagement strategies and identifying new approaches that can enhance community acceptance, which requires collaboration between different stakeholders involved in the project.
3. Anticipatory - The question requires an anticipatory approach to identify potential challenges and risks that may arise during the implementation of the project and develop strategies to mitigate them.
4. Integrated Problem Solving - The question requires an integrated problem-solving approach to address not only the issue of sabotage and resistance to installation but also the broader objectives of the project, including promoting gender equality, reducing inequalities, and promoting sustainable communities.
5. Strategic - The question requires a strategic approach to identify the communities where roll-out has struggled and develop strategies to improve community acceptance.
6. Systems Thinking - The question requires a systems thinking approach to consider the broader social, economic, and environmental systems that are impacted by the implementation of the project.

Overall, this question highlights the importance of sustainability competencies in addressing complex sustainability challenges and promoting sustainable development

On a recent visit with your aunt, she told you about this new organization she had joined called an Inter-generational Self-help Club (ISHC). She described how this inter-generational organization is empowered to design, conduct, and manage community development activities in partnership with NGOs and government agencies. You are particularly excited by how they focus on being inclusive. She has put you in contact with the sponsoring NGO and their representative told you that they would be very interested in supporting the creation of an ISHC in your community if you would be willing to take the lead.

Q19. When you tell your friend about this opportunity, they ask if this is really your job as a random community member to organize? Shouldn’t the formation of an ISHC be left to the professionals? Imagine yourself in this situation. Which 2 statements best describe your EMOTIONS? Comfortable leading this initiative only if the supporting NGO tells me how it should be implemented based on their experiences elsewhere. Confident that you have all the knowledge, skills, and abilities to design and implement an ISHC on your own if you are given financial support. Excited by the idea of bringing together a diverse and inclusive group of people to tackle our community’s problems. Confident that despite the challenges, you can lead this project by building a strong team. Intimidated by the prospect of leading a complex sustainability project such as an ISHC and by all its participants and stakeholders. Apprehensive because complex sustainability projects should be led by professionals, not me.

The two statements that best describe your EMOTIONS in this situation are:

1. Excited by the idea of bringing together a diverse and inclusive group of people to tackle our community’s problems.
2. Confident that despite the challenges, you can lead this project by building a strong team.

19a. To which all SDGs and specific targets within those SDGs, this question possibly aligns with?

The question is related to several of the United Nations' Sustainable Development Goals (SDGs), which are a universal call to action to end poverty, protect the planet, and ensure that all people enjoy peace and prosperity. Specifically, the question aligns with the following SDGs and specific targets:

1. SDG 1: No Poverty - Target 1.4: Ensure that all men and women, in particular, the poor and the vulnerable, have equal rights to economic resources, as well as access to basic services, ownership, and control over land and other forms of property, inheritance, natural resources, appropriate new technology, and financial services including microfinance.
2. SDG 5: Gender Equality - Target 5.5: Ensure women’s full and effective participation and equal opportunities for leadership at all levels of decision-making in political, economic, and public life.
3. SDG 10: Reduced Inequalities - Target 10.2: By 2030, empower and promote the social, economic, and political inclusion of all, irrespective of age, sex, disability, race, ethnicity, origin, religion, or economic or other status.
4. SDG 17: Partnerships for the Goals - Target 17.16: Enhance the global partnership for sustainable development, complemented by multi-stakeholder partnerships that mobilize and share knowledge, expertise, technology, and financial resources, to support the achievement of the sustainable development goals in all countries, particularly developing countries.

The Inter-generational Self-help Club (ISHC) described in the question aims to empower people of all ages and backgrounds to work together to address community development issues, which aligns with the above SDGs and targets. The focus on inclusivity, partnership, and community development is crucial for achieving sustainable development, which is the main objective of the SDGs.

19b. Which of the eight cross-cutting sustainability competencies like: Self-Awareness Collaboration Critical Thinking Anticipatory Integrated Problem Solving Normative Strategic Systems Thinking are addressed by this question?

The following cross-cutting sustainability competencies are addressed by this question:

1. Collaboration: The question describes an inter-generational organization that works in partnership with NGOs and government agencies to design, conduct, and manage community development activities. Collaboration is essential for achieving sustainable development and addressing complex issues that require a collective effort.
2. Critical Thinking: The question asks whether the formation of an ISHC should be left to professionals or whether a random community member can organize it. This question requires critical thinking to assess the feasibility of organizing an ISHC, the available resources, and the potential benefits and drawbacks.
3. Normative: The question raises ethical considerations related to community development, such as inclusivity, social justice, and empowerment. These normative considerations are essential for ensuring that sustainability initiatives align with ethical principles and promote the common good.
4. Strategic: The question involves strategic thinking about how to design and implement an ISHC effectively, considering factors such as available resources, stakeholder engagement, and sustainability outcomes.
5. Systems Thinking: The question addresses the complexity of sustainability challenges and the need to approach them from a systems perspective, considering the interconnections and interdependencies between social, economic, and environmental factors. The ISHC described in the question aims to address community development issues by bringing together people from different backgrounds and age groups and working in partnership with multiple stakeholders, which requires a systems thinking approach.

Q20. You and the NGO representative have a series of discussions about how to go about the process of implementing an ISHC. They share with you some of the different ways in which past communities have implemented their ISHCs. You really want the ISHC to be both successful and maximally transformative for your community and thus are looking to design the process carefully. Given this which of the following advice should you follow? Choose the best 2 answers. The first step of the process should be to clearly articulate the strategy for setting up the ISHC based on the successes that others have had. Integrated problem-solving is just about planning and developing strategies, the implementation is a separate and independent process. There is only one correct order of steps that will assure a successful outcome, it’s outlined in the ISHC guidebook. Including diverse voices in the process must be done even when it will slow down and generate conflicts during the process. You need to be an expert at each step of the process in order to be able to integrate and facilitate the process. It is important to develop a vision and an understanding of the current state before designing strategies to bridge the gap between them.

The two best pieces of advice to follow for creating a successful and transformative ISHC in your community are:

1. Including diverse voices in the process must be done even when it will slow down and generate conflicts during the process. This is important because an ISHC is designed to be inter-generational and inclusive, and it will be more successful if it reflects the diverse perspectives, experiences, and needs of the community it serves. It may take more time and effort to include diverse voices, but the end result will be a more robust and effective organization that can truly serve the community.
2. It is important to develop a vision and an understanding of the current state before designing strategies to bridge the gap between them. This is because an ISHC is designed to be a community-driven and community-led organization. The community's needs, strengths, and challenges should inform the vision and direction of the organization. By starting with a clear understanding of the current state and the community's vision for the future, the ISHC can develop strategies and activities that are relevant, effective, and sustainable.

The other pieces of advice given are not necessarily accurate or helpful. There is no one correct order of steps that will assure a successful outcome, as each community is unique and requires a tailored approach. Integrated problem-solving involves both planning and implementation, and these processes are interconnected. It is not necessary to be an expert at each step of the process to facilitate the process, as there are resources and support available from the sponsoring NGO and other community partners. While it may be tempting to rely on the ISHC guidebook, it is important to adapt strategies and approaches to the unique needs and context of the community.

20a. To which all SDGs and specific targets within those SDGs, this question possibly aligns with?

The question aligns with several SDGs and specific targets within those SDGs, including:

1. SDG 1: No Poverty - the focus on community development activities and partnership with NGOs and government agencies can contribute to reducing poverty in the community.
2. SDG 3: Good Health and Well-being - an inter-generational self-help club can promote healthy living and well-being among the members of the community.
3. SDG 4: Quality Education - an inter-generational self-help club can provide opportunities for education and skill-building, particularly for younger and older members of the community.
4. SDG 5: Gender Equality - the focus on inclusion and diverse voices aligns with the goal of promoting gender equality.
5. SDG 8: Decent Work and Economic Growth - community development activities and partnerships with NGOs and government agencies can contribute to economic growth and job creation in the community.
6. SDG 10: Reduced Inequalities - the focus on inclusion and diverse voices aligns with the goal of reducing inequalities within the community.
7. SDG 16: Peace, Justice, and Strong Institutions - the focus on community-driven and community-led organizations can contribute to building strong and inclusive institutions that promote peace and justice in the community.

Some specific targets that align with the question include:

- Target 1.1: By 2030, eradicate extreme poverty for all people everywhere, currently measured as people living on less than $1.25 a day.
- Target 3.4: By 2030, reduce by one third premature mortality from non-communicable diseases through prevention and treatment and promote mental health and well-being.
- Target 4.4: By 2030, substantially increase the number of youth and adults who have relevant skills, including technical and vocational skills, for employment, decent jobs and entrepreneurship.
- Target 5.5: Ensure women’s full and effective participation and equal opportunities for leadership at all levels of decision-making in political, economic and public life.
- Target 8.3: Promote development-oriented policies that support productive activities, decent job creation, entrepreneurship, creativity and innovation, and encourage the formalization and growth of micro-, small- and medium-sized enterprises, including through access to financial services.
- Target 10.2: By 2030, empower and promote the social, economic and political inclusion of all, irrespective of age, sex, disability, race, ethnicity, origin, religion or economic or other status.
- Target 16.7: Ensure responsive, inclusive, participatory and representative decision-making at all levels.

20b. Which of the eight cross-cutting sustainability competencies like: Self-Awareness Collaboration Critical Thinking Anticipatory Integrated Problem Solving Normative Strategic Systems Thinking are addressed by this question?

The following cross-cutting sustainability competencies are addressed by this question:

1. Collaboration: The question highlights the importance of partnership with NGOs and government agencies in designing, conducting, and managing community development activities. The focus on inclusion and diverse voices also emphasizes the need for collaboration and working together to create an effective and inclusive organization.
2. Integrated Problem Solving: The question acknowledges that implementing an ISHC involves both planning and implementation, and that these processes are interconnected. This requires an integrated problem-solving approach that considers the unique needs and context of the community.
3. Normative: The question highlights the importance of inclusivity and diversity in the process of creating an ISHC. This aligns with the normative competency of sustainability, which emphasizes the importance of social justice, equity, and human rights.
4. Strategic: The question emphasizes the need for a clear strategy and vision for setting up the ISHC based on past successes, the current state, and the needs of the community. This requires a strategic approach that considers long-term goals and the resources needed to achieve them.
5. Self-awareness: Although not explicitly mentioned, creating an ISHC requires self-awareness in terms of understanding one's own biases and assumptions, as well as recognizing the needs and perspectives of others in the community. This is important for creating an inclusive and effective organization.
6. Critical Thinking: Creating an ISHC requires critical thinking skills to analyze the needs and challenges of the community, evaluate different strategies and approaches, and make informed decisions based on available evidence.
7. Anticipatory: The question implies a forward-thinking approach to community development, in which the ISHC is designed to address current needs and anticipate future challenges and opportunities.
8. Systems Thinking: Creating an ISHC requires a systems thinking approach, in which the organization is designed to be integrated into the larger social, economic, and environmental systems of the community. This requires an understanding of the interrelationships between different components of the system and how they affect each other

Q21. In one of your conversations with a local health care provider, you explain that the ISHC intends to address multiple, complex socio-economic and environmental problems concurrently. The healthcare provider representative wonders why a multifunctional organization would produce better outcomes than a specialty-focused one such as themselves. How would you respond? Choose the best 2 answers. You agree with them that multiple specializations in each domain (such as health care clinics) offer the best solution to addressing sustainability problems. You explain that making durable, and effective change requires finding root causes and specifying these intervention points, an analysis which can rarely be done within the confines of a single domain. You assure them that your analysis will not include input from non-experts. You explain that taking a systems approach to problems is best, but it requires sophisticated modeling techniques which is why it is not done much. You agree that a systems analysis is too challenging because you have to identify every possible element and connection between them within the community no matter how trivial. You explain that there are feedback loops between different domains such as health and poverty and they can be more effectively addressed concurrently.

The best two answers are:

1. You explain that making durable, and effective change requires finding root causes and specifying these intervention points, an analysis which can rarely be done within the confines of a single domain.
2. You explain that there are feedback loops between different domains such as health and poverty and they can be more effectively addressed concurrently.

These answers highlight the benefits of taking a holistic approach to community development and problem-solving. By addressing multiple socio-economic and environmental problems concurrently, the ISHC can leverage feedback loops and address root causes, rather than just treating symptoms. This approach allows for more durable and effective change to be made, rather than just addressing a single issue in isolation. Additionally, by including a diverse range of stakeholders and experts, the ISHC can benefit from a broader range of perspectives and expertise, leading to more innovative and effective solutions.

21a. To which all SDGs and specific targets within those SDGs, this question possibly aligns with

The creation of an Inter-generational Self-help Club (ISHC) and its focus on community development activities in partnership with NGOs and government agencies aligns with multiple Sustainable Development Goals (SDGs) and specific targets within those SDGs.

Some of the SDGs and specific targets that this initiative may align with include:

1. SDG 1 - No Poverty: specifically target 1.4 which aims to ensure that all men and women, particularly the poor and vulnerable, have equal rights to economic resources, as well as access to basic services, ownership and control over land and other forms of property, inheritance, natural resources, appropriate new technology and financial services including microfinance.
2. SDG 3 - Good Health and Well-being: particularly targets 3.8 which focuses on achieving universal health coverage, including financial risk protection, access to quality essential health care services and access to safe, effective, quality and affordable essential medicines and vaccines for all.
3. SDG 4 - Quality Education: particularly target 4.7 which aims to ensure that all learners acquire the knowledge and skills needed to promote sustainable development, including, among others, through education for sustainable development and sustainable lifestyles, human rights, gender equality, promotion of a culture of peace and non-violence, global citizenship, and appreciation of cultural diversity and of culture's contribution to sustainable development.
4. SDG 10 - Reduced Inequalities: particularly target 10.3 which aims to ensure that equal opportunity and reduce inequalities of outcome, including by eliminating discriminatory laws, policies, and practices and promoting appropriate legislation, policies, and action in this regard.
5. SDG 11 - Sustainable Cities and Communities: particularly target 11.3 which focuses on enhancing inclusive and sustainable urbanization and capacity for participatory, integrated and sustainable human settlement planning and management in all countries.
6. SDG 17 - Partnerships for the Goals: specifically target 17.16 which aims to enhance the global partnership for sustainable development, complemented by multi-stakeholder partnerships that mobilize and share knowledge, expertise, technology, and financial resources, to support the achievement of the sustainable development goals in all countries.

These are just a few examples of the possible SDGs and specific targets that this initiative may align with. The ISHC's focus on being inclusive and addressing multiple socio-economic and environmental problems concurrently demonstrates a commitment to promoting sustainable development and contributing to the achievement of the SDGs.

21b. Which of the eight cross-cutting sustainability competencies like: Self-Awareness Collaboration Critical Thinking Anticipatory Integrated Problem Solving Normative Strategic Systems Thinking are addressed by this question?

The following cross-cutting sustainability competencies are addressed by this question:

1. Collaboration: The question highlights the importance of partnerships and collaboration between different stakeholders such as NGOs, government agencies, and community members to address socio-economic and environmental problems.
2. Critical Thinking: The question emphasizes the need for a holistic approach to community development that addresses root causes and leverages feedback loops between different domains. This requires critical thinking skills to analyze complex systems and identify key intervention points.
3. Integrated Problem Solving: The creation of an Inter-generational Self-help Club (ISHC) and its focus on addressing multiple socio-economic and environmental problems concurrently demonstrates an integrated problem-solving approach.
4. Normative: The question implies a normative approach to sustainability, where community development activities aim to promote sustainable development and contribute to the achievement of the SDGs.
5. Strategic: The question highlights the need for a strategic approach to community development that takes into account the interconnections between different domains and leverages partnerships to achieve sustainable outcomes.
6. Systems Thinking: The question emphasizes the need for a systems thinking approach to community development that considers the interconnections between different domains and addresses feedback loops to achieve sustainable outcomes.

Therefore, the question addresses multiple cross-cutting sustainability competencies, including Collaboration, Critical Thinking, Integrated Problem Solving, Normative, Strategic, and Systems Thinking.

Q22. You and your team have gathered for a planning session for an upcoming workshop. The workshop will be attended by local partners, government representatives, and community members. The primary objective of the workshop is to develop a vision for your community in 2030. With that in hand, you will be able to design the role that your ISCH can play in making it happen. What are some things that you and your team should keep in mind when planning the visioning workshop? Choose the best 2 answers. To build a vision, most of the time should be given for participants to discuss all the problems they see with the way things are right now. You must prepare to elicit participants’ values and work with them to prioritize and negotiate the values as a core part of the vision. Don’t restrain the participant’s ideas, visions are meant for inspiration and do not have to have any grounding in reality. The main purpose of such a session is to extract from participants what they want in the future so whether they leave inspired or not is immaterial. You should either rigorously structure the workshop ahead of time or be prepared for substantial work afterwards to create a vision that is coherent out of all the ideas from participants. Sustainability visions should be based exclusively on scientific facts (e.g., climate change) not the values of individuals or groups.

To plan the visioning workshop, the team should keep in mind the following:

1. To build a vision, most of the time should be given for participants to discuss all the problems they see with the way things are right now. It is important to allow participants to share their concerns and identify the current challenges faced by the community. This will provide a starting point for developing a vision that addresses the issues faced by the community.
2. You must prepare to elicit participants’ values and work with them to prioritize and negotiate the values as a core part of the vision. It is crucial to understand the values of the community members and ensure that they are reflected in the vision. By involving the community in the visioning process, it becomes a shared vision that everyone can work towards.

Therefore, the best answers are:

- To build a vision, most of the time should be given for participants to discuss all the problems they see with the way things are right now.
- You must prepare to elicit participants’ values and work with them to prioritize and negotiate the values as a core part of the vision.

22a. To which all SDGs and specific targets within those SDGs, this question possibly aligns with?

This question aligns with several SDGs, primarily SDG 16: Peace, Justice and Strong Institutions, which focuses on promoting inclusive and participatory decision-making at all levels. More specifically, the visioning workshop aims to engage local partners, government representatives, and community members in the development of a shared vision for the community, which aligns with SDG 16.7: "Ensure responsive, inclusive, participatory, and representative decision-making at all levels".

The workshop also aligns with SDG 17: Partnerships for the Goals, which emphasizes the importance of collaborative efforts among different stakeholders to achieve sustainable development. The workshop involves partnership between the ISHC, NGOs, and government agencies to design and manage community development activities.

Moreover, the visioning workshop aligns with the specific targets of SDG 11: Sustainable Cities and Communities, which aims to make cities and human settlements inclusive, safe, resilient, and sustainable. The development of a shared vision for the community in 2030, which involves local partners, government representatives, and community members, can help ensure that the community development activities are inclusive, safe, resilient, and sustainable, in line with SDG 11.7: "By 2030, provide universal access to safe, inclusive, and accessible, green and public spaces, particularly for women and children, older persons, and persons with disabilities".

22b. Which of the eight cross-cutting sustainability competencies like: Self-Awareness Collaboration Critical Thinking Anticipatory Integrated Problem Solving Normative Strategic Systems Thinking are addressed by this question?

The question mainly addresses two of the eight cross-cutting sustainability competencies, which are collaboration and systems thinking.

Collaboration is essential in this question as it involves working with different stakeholders, including local partners, government representatives, community members, and NGOs to develop a shared vision for the community. Collaboration requires individuals to work together, listen to different perspectives, and negotiate priorities to achieve a common goal.

Systems thinking is also important in this question as it involves considering the interconnectedness of different factors and their impacts on sustainability. Developing a shared vision for the community requires understanding the complex social, economic, and environmental systems that influence the community's development. Systems thinking helps individuals identify the interrelationships and trade-offs among different factors and consider the long-term consequences of their decisions.

While self-awareness, critical thinking, anticipatory, integrated problem solving, normative, and strategic competencies may be useful in planning and implementing community development activities, they are not directly addressed in this question

Q23. Almost everyone came away from the visioning workshop inspired. After some work you have crafted a wonderful narrative that brings together themes of intergenerational solidarity, healthy families, and prospering communities. You have already had several local partners, who were on the fence about participating, contact you to say that after the workshop they are excited to be fully onboard. What would be the key next steps to take? Choose the best 2 answers. Develop and solidify the new local partners as well as those who had previously committed into a supportive network which is dedicated to success. Revisit the current state analysis your team crafted and develop some strategies which would enable the ISHC to get the community from the present to the articulated future vision. Work to develop a foolproof implementation plan that will definitely achieve the vision. Develop your strategy based exclusively on your understanding of the current state; the vision served its purpose in inspiring stakeholders and getting them on board. If possible, avoid any discussions of the local politics and power dynamics in the strategic plans as it might threaten some potential partners. Take advantage of the good momentum to announce the launch of the ISHC in the community and open it up as soon as possible.

The best next steps to take would be to:

1. Develop and solidify the new local partners as well as those who had previously committed into a supportive network which is dedicated to success. This involves building relationships, fostering trust, and creating a sense of ownership and shared responsibility among the partners. By doing so, the ISHC can ensure that all partners are invested in the success of the initiative and are working together towards a common goal.
2. Revisit the current state analysis your team crafted and develop some strategies which would enable the ISHC to get the community from the present to the articulated future vision. This involves analyzing the current state of the community, identifying gaps and challenges, and developing strategies to address them. By doing so, the ISHC can create a roadmap for achieving its vision, ensuring that its efforts are focused, targeted, and effective.

It is important to note that while developing the implementation plan, it is essential to consider local politics and power dynamics. It is critical to engage with all stakeholders, including those who may be opposed or hesitant about the initiative, to understand their concerns and perspectives. By doing so, the ISHC can develop a plan that is sensitive to the community's needs and aspirations and is more likely to succeed.

Announcing the launch of the ISHC and opening it up as soon as possible is also important. However, it should not be done at the expense of developing a solid strategy and network of committed partners. Rushing into implementation without a clear plan and strong partnerships may lead to failures and setbacks, undermining the credibility and effectiveness of the ISHC.

23a. To which all SDGs and specific targets within those SDGs, this question possibly aligns with?

The question is related to the following Sustainable Development Goals (SDGs):

1. SDG 17 - Partnerships for the Goals: The question highlights the importance of partnerships between NGOs, government agencies, and community members to achieve community development goals.
2. SDG 3 - Good Health and Well-being: The question mentions the importance of healthy families and prospering communities in the narrative developed for the ISHC.
3. SDG 10 - Reduced Inequalities: The question mentions the ISHC's focus on being inclusive and inter-generational, which aligns with the goal of reducing inequalities.
4. SDG 11 - Sustainable Cities and Communities: The ISHC's community development activities are aimed at creating prosperous and sustainable communities.
5. SDG 16 - Peace, Justice, and Strong Institutions: The question mentions the need to be aware of local politics and power dynamics when developing a strategy for the ISHC, which aligns with the goal of promoting peace, justice, and strong institutions.

While there are no specific targets mentioned in the question, the ISHC's focus on community development and partnership aligns with several targets under the above-mentioned SDGs.

23b. Which of the eight cross-cutting sustainability competencies like: Self-Awareness Collaboration Critical Thinking Anticipatory Integrated Problem Solving Normative Strategic Systems Thinking are addressed by this question?

The following cross-cutting sustainability competencies are addressed by this question:

1. Collaboration: The question highlights the importance of collaboration between NGOs, government agencies, and community members to achieve community development goals.
2. Anticipatory: The question mentions the need to develop strategies to address current gaps and challenges and enable the ISHC to get the community from the present to the future vision, which aligns with the anticipatory competency of sustainability.
3. Integrated problem solving: The question mentions the need to develop a foolproof implementation plan that will achieve the vision. This requires integrated problem-solving skills to identify potential challenges and develop solutions that are holistic and systemic.
4. Strategic: The question mentions the need to develop a strategy based on the current state analysis and take into account local politics and power dynamics, which requires strategic thinking to achieve the vision.
5. Systems thinking: The question mentions the need to analyze the current state of the community and develop strategies to address gaps and challenges, which requires systems thinking to understand how different components of the system are interdependent and how they can be leveraged to achieve the desired outcomes.

Q24. You and your team have gathered sufficient funding to launch your community’s ISHC and to your surprise, you were asked to be hired on as its interim director (and only full-time employee) until the formal hiring process of the director position starts (to which you could apply, too). You have a lot of confidence in the implementation plan which you have developed but its success depends on the collaborative coordination of numerous local partners, government agencies, and community members, thus on their full buy-in of the plan. What would be some actions or practices that you would put in place to ensure successful implementation? Choose the best 2 answers. Avoid wasting resources on monitoring progress and interim objectives; rather invest resources to support meeting the end goals as articulated in the project vision. Keep all the various entities working on implementation separate from each other as much as possible so as to avoid any disagreements and assure that everyone is following the original plan. Don’t invest your limited time in community events as you have already elicited input during planning and don’t need to include stakeholders in the implementation. Take the needed time to resolve conflicts and disputes among team members even if it slows progress in the short term. Work to assure effective and compassionate communication both among team members and continuously with relevant stakeholders. Remind yourself that as long as the project team stays true to the original sustainability vision, how the plan is implemented is not materially important for its success.

The two best answers would be:

1. Take the needed time to resolve conflicts and disputes among team members even if it slows progress in the short term. It's essential to have a cohesive team to ensure successful implementation. Addressing and resolving conflicts early on will help to prevent misunderstandings and foster a collaborative environment. Even though it may slow down progress in the short term, it will likely save time and resources in the long run.
2. Work to assure effective and compassionate communication both among team members and continuously with relevant stakeholders. Clear and consistent communication is key to ensuring everyone is on the same page and working towards the same goals. Maintaining open and respectful communication with stakeholders, community members, and team members can help to build trust, gather feedback, and address concerns. It is important to listen actively, be transparent, and keep everyone informed throughout the implementation process.

24a. To which all SDGs and specific targets within those SDGs, this question possibly aligns with?

This question aligns with multiple Sustainable Development Goals (SDGs) and their specific targets.

1. SDG 1: No Poverty - The Inter-generational Self-help Club (ISHC) is designed to empower the community to conduct and manage development activities in partnership with NGOs and government agencies. By focusing on community development, ISHC can help reduce poverty and promote economic growth in the community.
2. SDG 3: Good Health and Well-being - ISHC's community development activities can include health initiatives that aim to improve the well-being of community members.
3. SDG 4: Quality Education - ISHC's community development activities can also include education initiatives that aim to improve access to quality education for community members.
4. SDG 5: Gender Equality - ISHC's inclusive approach to community development can help promote gender equality and empower women and girls.
5. SDG 10: Reduced Inequalities - ISHC's inter-generational approach to community development can help reduce inequalities by promoting the active participation of people from all age groups and backgrounds.
6. SDG 17: Partnerships for the Goals - ISHC's partnership with NGOs and government agencies aligns with SDG 17, which focuses on the importance of building partnerships to achieve sustainable development goals.

24b. Which of the eight cross-cutting sustainability competencies like: Self-Awareness Collaboration Critical Thinking Anticipatory Integrated Problem Solving Normative Strategic Systems Thinking are addressed by this question?

This question addresses several cross-cutting sustainability competencies, including:

1. Collaboration - The implementation plan developed by the team in this question requires the collaborative coordination of numerous local partners, government agencies, and community members to ensure successful implementation.
2. Critical Thinking - The interim director in this question will need to use critical thinking skills to ensure that the implementation plan is successful. This includes assessing the plan, identifying any potential challenges or issues, and developing strategies to address them.
3. Anticipatory - The interim director will also need to be anticipatory, meaning they must anticipate potential challenges and issues that may arise during implementation and plan accordingly.
4. Integrated Problem Solving - The implementation plan developed by the team in this question requires an integrated problem-solving approach to address the complex and interconnected issues that may arise during implementation.
5. Strategic - The interim director will need to use strategic thinking to ensure that the implementation plan is successful. This includes setting clear goals and objectives, developing strategies to achieve them, and monitoring progress towards these goals.
6. Systems Thinking - The implementation plan developed by the team in this question requires systems thinking, meaning they must consider the interconnectedness of various systems, organizations, and stakeholders involved in the implementation process.

In summary, this question addresses several sustainability competencies, including collaboration, critical thinking, anticipatory, integrated problem-solving, strategic, and systems thinking.
